# Detoxification of conifer antimicrobial defenses promotes entomopathogenic fungus infection of bark beetles

**DOI:** 10.1073/pnas.2525513122

**Published:** 2025-12-29

**Authors:** Ruo Sun, Baoyu Hu, Yoko Nakamura, Michael Reichelt, Xingcong Jiang, Katrin Luck, Christian Paetz, Jonathan Gershenzon

**Affiliations:** ^a^Department of Biochemistry, Max Planck Institute for Chemical Ecology, Jena 07745, Germany; ^b^Department of Natural Product Biosynthesis, Max Planck Institute for Chemical Ecology, Jena 07745, Germany; ^c^Research Group Biosynthesis/NMR, Max Planck Institute for Chemical Ecology, Jena 07745, Germany; ^d^Department of Evolutionary Neuroethology, Max Planck Institute for Chemical Ecology, Jena 07745, Germany

**Keywords:** plant–insect–microbe interactions, plant secondary metabolite, phenolic metabolism, detoxification, entomopathogenic fungus

## Abstract

After consumption by herbivores, plant antimicrobial defense compounds may enhance herbivore immunity to pathogenic microbes. In conifer-bark beetle interactions, beetles ingest large quantities of phloem tissue containing high concentrations of antimicrobial phenolic glucosides, such as stilbenes and flavonoids. It is not known, however, if these compounds increase bark beetle resistance to pathogens. We showed that Eurasian spruce bark beetles (*Ips typographus*) attacking Norway spruce (*Picea abies*) hydrolyze phenolic glucosides to their corresponding aglucones increasing their antifungal activity. However, the entomopathogen *Beauveria bassiana*, a natural fungal parasite of these beetles, detoxifies stilbene and flavonoid aglucones by forming methylglucoside derivatives. A two-step pathway involving a UDP-glycosyltransferase and an *O*-methyltransferase produces phenolic *O*-methylglucosides that are no longer toxic to *B. bassiana* and are stable to β-glucosidase action. Compared to wild-type strains of *B. bassiana*, mutant strains knocked out in the genes of this pathway exhibited decreased methylglucoside formation, slower growth on medium containing phenolic compounds, and reduced virulence toward bark beetles. Hence, methylglucosylation of plant-derived phenolics is a detoxification process that significantly increases the ability of *B. bassiana* to parasitize host insects consuming plant tissue high in phenolics, such as conifer phloem. This is one of the few examples of an entomopathogen that is able to resist the plant-derived defenses of an insect host.

## Significance

Plants produce antimicrobial compounds to defend themselves against pathogens, and herbivorous insects may gain protection from their own pathogens by consuming these compounds. We found that bark beetles enzymatically convert some antimicrobial phenolic compounds of spruce trees into more potent antimicrobial derivatives. However, an insect-killing fungus counters these phenolic compounds with a two-step detoxification pathway to produce methylglucoside derivatives. Knocking out this fungal pathway by genetic transformation reduces the virulence of the fungus on bark beetles, proving the pathway’s importance for successful fungal infection.

Plants produce numerous metabolites that act as defenses to herbivores and pathogens (1). Once ingested by herbivores, these compounds can impact other organisms at higher trophic levels. For instance, some insect herbivores are well known to sequester plant defense compounds as protection against their enemies (2). However, even nonsequestering herbivores that feed on plant defenses can negatively affect the performance of predators and parasitoids depending on how they metabolize the ingested compounds (3, 4). With the realization that some animals ingest plant chemicals to self-medicate against disease (5), interest has grown in whether insects feeding on plant diets high in antimicrobial defenses are prophylactically protected against pathogenic microbes (6). For instance, many years ago the larvae of the invasive spongy moth (*Lymantria dispar*, formerly known as the gypsy moth), which may feed on tree foliage high in certain phenolic compounds in their adventive range in North America, were shown to be less susceptible to a virus than larvae with a diet lacking in such phenolic compounds (7). However, results from later studies were inconsistent, and work on other experimental systems on whether plant antimicrobials ingested by herbivores decrease pathogen susceptibility has shown no strong trends (6, 8). Moreover, researchers have not often checked whether herbivore digestive processes alter the antimicrobial activity of ingested defenses, or whether pathogenic microbes possess any resistance to these substances.

A good example of insects that feed on high concentrations of antimicrobial defenses is conifer bark beetles, whose outbreaks in temperate forests have increased dramatically in recent years due to rising global temperatures (9). Conifer bark beetles colonize phloem tissue containing high amounts of phenolic compounds. For instance, the Eurasian spruce bark beetle (*Ips typographus*) feeds on the phloem of Norway spruce (*Picea abies*), which contains stilbenoid and flavonoid glucosides at concentrations up to 5% of dry weight (10). Stilbenes and flavonoids are known to have antibacterial and antifungal activity (11, 12), and are well established to defend plants against pathogenic microbes (13–15). Yet we know little about whether these abundant phenolic compounds are metabolized by bark beetles and whether they affect bark beetle-associated pathogens.

The entomopathogenic fungus *Beauveria bassiana* is a cosmopolitan soil-borne pathogen that infects a wide range of insect hosts, including bark beetles (16–21). It has been widely used as a biological control agent instead of traditional insecticides and many different strains have been isolated. Due to its natural occurrence in *Ips typographus* bark beetles in spruce forests, *B. bassiana* has been evaluated for its potential in managing bark beetle population outbreaks (20–22), but most field applications have not been successful. It is not yet studied whether this entomopathogenic fungus has a mechanism to resist the toxic effects of the antimicrobial phenolic compounds originating from the host tree of the bark beetle.

Fungi are known to detoxify a wide range of plant defense compounds, including phenolics such as stilbenes, pterocarpans, isoflavones, and other flavonoids (23). For example, stilbenes have been reported to be oxidized by a laccase from *Botrytis cinerea* (24), and to be cleaved by a catechol dioxygenase activity from *Endoconidiophora polonica* to form ring-opened, muconoid-type products that are further degraded (25). While glycosylation of toxins is not as common in fungi as in other organisms, the flavonoid sakuranetin is conjugated with xylose by *Rhizoctonia solani* to form a nontoxic derivative (26). Several fungi of the order Hypocreales, including *B. bassiana*, convert various phenolic compounds to methylglucoside derivatives (27, 28). Anthraquinones, benzenediol lactones, flavonoids, and stilbenoids are subject to glucosylation and subsequent *O*-methylation on the glucose moiety. However, it is not yet known if *B. bassiana* can detoxify the specific phenolic substances in bark beetles derived originally from spruce phloem and whether this affects its virulence in bark beetle infection.

In this study, we first investigated the metabolic fate of spruce phenolic compounds in *I. typographus* bark beetles, and then determined the role of these substances in bark beetle susceptibility to *B. bassiana*. We isolated strains of this entomopathogen from the carcasses of fungal-killed beetles and demonstrated their detoxification of spruce and bark beetle-derived phenolic compounds. We then identified candidate genes involved in phenolic metabolism, heterologously expressed the recombinant proteins for assay, and confirmed their in vivo role by using gene knockout mutants. The availability of these mutants allowed us to establish the contribution of this detoxification process to fungal virulence in bark beetles.

## Results

### Bark Beetles Deglycosylate the Phenolic Glucosides of Their Host Tree.

**Fig. 1.**
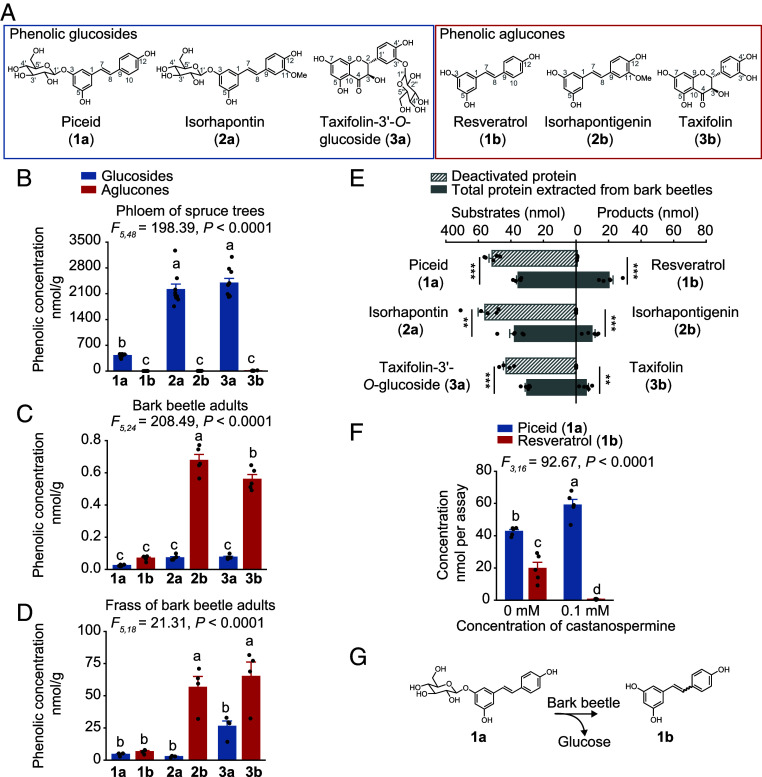
Bark beetles hydrolyze spruce phenolic glucosides to their corresponding aglucones. (*A*) Structural representations of the major phenolic glucosides and corresponding aglucones. (*B*–*D*) Major phenolic compounds in the phloem of Norway spruce trees (*n* = 8) (*B*), in the bodies of adult bark beetles (*n* = 5) (*C*), and in bark beetle frass (*n* = 4) (*D*). (*E*) Deglycosylation of phenolic compounds supplied to crude protein extracts of bark beetles incubated in vitro (*n* = 5). Boiled extracts were used as negative controls (*n* = 5). (*F*) Inhibition of glucosidase activity in the crude protein extracts by the α/β-glucosidase inhibitor, castanospermine (*n* = 5). (*G*) Pathway for the hydrolysis of the phenolic glucoside piceid, resulting in the formation of resveratrol. Significant differences between means (±SE) were determined using one-way ANOVA followed by Tukey’s HSD tests in *B*–*D* and *F*, and two-tailed *t* tests in *E*. Different lowercase letters (*P* < 0.05) or asterisks (**P < 0.01; ***P < 0.001) denote statistically significant differences.

We first assessed the abundance of phenolic compounds in the bark of bark beetle host trees and investigated their metabolism by beetles. In the phloem of Norway spruce (*Picea abies*) (the tissue fed upon by bark beetles), phenolic glucosides were present in high concentrations, whereas the corresponding phenolic aglucones were significantly less abundant. Specifically, the levels of the major compounds, the stilbenes piceid (**1a**) and isorhapontin (**2a**), and the flavonoid taxifolin-3’-*O*-glucoside (**3a**), were significantly higher than those of their corresponding aglucones, resveratrol (**1b**), isorhapontigenin (**2b**), and taxifolin (**3b**) (Fig. 1 *A* and *B*). *Ips typographus* bark beetles hydrolyzed the phenolic glucosides to the aglucones. There were significantly greater amounts of aglucones compared to phenolic glucosides in the bodies and frass of adult bark beetles (Fig. 1 *C* and *D*). This was also true for bark beetle larvae, larval frass, and newly emerged adults called callow adults (*SI Appendix*, Fig. S1 *A*–*C*). To confirm the hydrolysis of the spruce-derived phenolic glucosides by bark beetles, a crude protein extract from beetles was incubated with the phenolic glucosides, **1a**, **2a**, and **3a**, and found to catalyze the hydrolysis of these glucosides into their corresponding aglucones, **1b**, **2b**, and **3b**, whereas no such activity was observed with heat-denatured (boiled) protein extracts (Fig. 1*E*). When castanospermine, an α/β-glucosidase inhibitor, was added to the protein extract in the presence of **1a**, the deglycosylation activity of bark beetle proteins toward the compound was suppressed (Fig. 1*F*). These results confirm that bark beetles possess a glucosidase activity capable of hydrolyzing spruce phenolic glucosides, leading to the release of the corresponding aglucones (Fig. 1*G*).

In addition to hydrolysis, the stilbenes were also isomerized. These diarylethenes occur in intact trees as glucosides with the ethene double bond having almost exclusively (>98%) an *E*-configuration (*SI Appendix*, Fig. S1*D*). However, both the *E*- and Z-isomers of the stilbene glucosides and their derived aglucones were detected in bark beetles and their frass with the *Z*-form present at approximately 5 to 10% of the *E*-form (*SI Appendix*, Fig. S1*D*). This suggests that bark beetle metabolism not only hydrolyzes the stilbene glucosides, but also drives their isomerization.

### An Entomopathogenic Fungus Metabolizes the Phenolic Aglucones to Methylglucoside Derivatives.

**Fig. 2.**
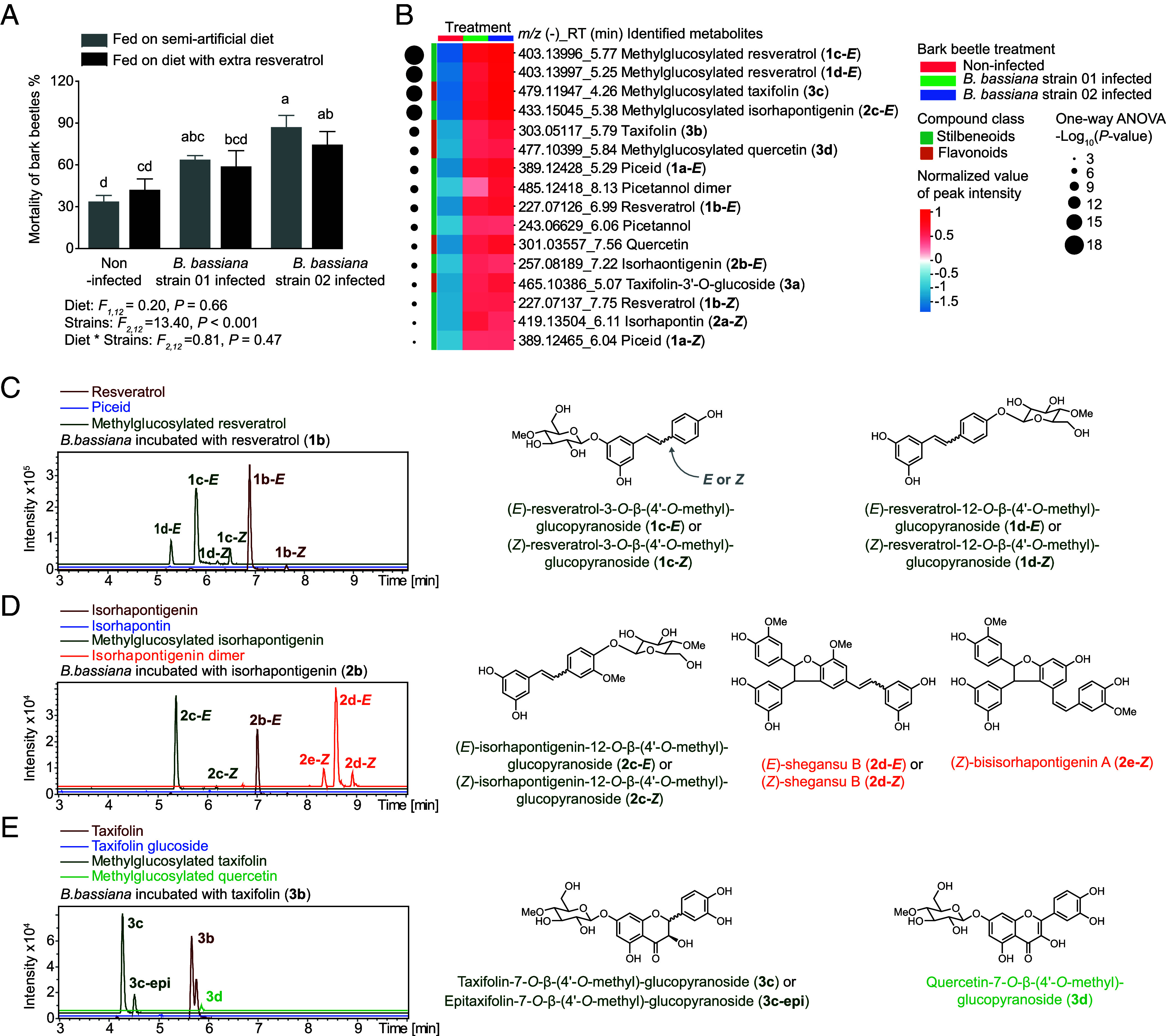
*Beauveria bassiana* metabolizes phenolic aglucones into methylglucosylated products. (*A*) Mortality of bark beetles inoculated with naturally isolated *B. bassiana* strains 01 and 02 compared to an uninfected control fed on a semiartificial diet containing a spruce bark phenolic mixture or diet supplemented with extra resveratrol (20 µmol/g), a stilbene aglucone, for 10 d (*n* = 3). (*B*) Heatmap of LC–MS/MS features from nontargeted UHPLC-qTOF-MS analyses of bark beetles infected with *B. bassiana* strains 01 and 02 compared to an uninfected control fed on a semiartificial diet (*n* = 8). Data are based on peak intensity, normalized by sample weight and log_10_-transformed. Metabolomic analyses and significantly different metabolites formed by *B. bassiana*–infected bark beetles compared to uninfected bark beetles are listed in the Dataset S1. (*C*–*E*) *B. bassiana* metabolism of the phenolic aglucones, resveratrol (*C*), isorhapontigenin (*D*), and taxifolin (*E*), growing on PDA plates. Depicted are extracted ion chromatograms from HPLC-qTOF-MS measurements of fungal extracts in the negative ionization mode. NMR analyses of products are presented in *SI Appendix*, Fig. S3 and Table S2; mass spectra of fungal products and phenolic standards are available in Edmond. **1b-*E*** indicates the *E*-isomer and **1b-*Z*** indicates the *Z*-isomer. Statistically significant differences between means (±SE) were determined using two-way ANOVA followed by Fisher’s LSD tests in *A*, and one-way ANOVA with Fisher’s LSD tests in *B*. Different lowercase letters denote statistically significant differences (*P* < 0.05).

The major phenolic compounds of Norway spruce bark, both stilbenes and flavonoids, are known to have antifungal activity (10, 29). To determine how ingestion of these compounds by bark beetles might impact fungal pathogens of the beetles, we isolated two fungi that had killed beetles in galleries of attacked trees and in our laboratory colony. These were both identified as strains of *Beauveria bassiana*, the well-known entomopathogen (*SI Appendix*, Fig. S2*A*). We then inoculated callow adult bark beetles with either *B. bassiana* strain and fed them on a semiartificial diet containing a natural mixture of phenolic substances from spruce bark. The content of phenolic compounds in this diet is listed in *SI Appendix*, Table S1. Inoculation with either *B. bassiana* strain led to increased mortality in bark beetles (Fig. 2*A*), even when an extra 20 µmol/g of the stilbene aglucone resveratrol (**1b**, approximately ten times the amount in the natural mixture) was added to the diet (Fig. 2*A*). Immunostaining of bark beetles infected with a *B. bassiana eGFP* strain, engineered from wild-type (WT) *B. bassiana* by overexpression of *eGFP*, revealed systemic fungal infection throughout the beetle body (*SI Appendix*, Fig. S2*B*).

Untargeted metabolite analyses were carried out on *B. bassiana*–infected and uninfected bark beetles using ultra-high-performance liquid chromatography coupled to quadrupole time-of-flight mass spectrometry (UHPLC-qTOF-MS). Infection altered the metabolite patterns in bark beetles compared to those of uninfected bark beetles, while there was no difference in metabolites between the beetles infected by either *B. bassiana* strain (*SI Appendix*, Fig. S2*C*). Infected bark beetles that fed on the semiartificial diet accumulated significantly higher amounts of phenolic compounds than uninfected beetles, including both stilbenes and flavonoids (Fig. 2*B* and S*I Appendix*, Fig. S2*D*).

The most abundant phenolic features in bark beetles after *B. bassiana* infection were identified as 4’-*O*-methylglucoside derivatives of the major phenolic compounds by mass spectrometry and NMR (Fig. 2 *C*–*E* and *SI Appendix*, Fig. S3 and Table S2). The same products were found after incubation of *B. bassiana* grown on a potato dextrose agar (PDA) plate with stilbene and flavonoid precursors. (*E*)-Resveratrol was methylglucosylated on either the 3- or 12- hydroxyl group, and the 3-*O*- and 12-*O*-methylglucosides of the corresponding *Z*-isomer were also detectable (Fig. 2*C* and *SI Appendix*, Fig. S4*A*). These transformations were confirmed by incubating *B. bassiana* with [^13^C_6_]resveratrol, which was converted to [^13^C_6_]resveratrol methylglucosides (*SI Appendix*, Fig. S4*B*). Among the other phenolic aglucones produced by *I. typographus* from spruce glucosides, (*E*)- and (*Z*)-isorhapontigenin (**2b**) were converted to 12-*O*-methylglucosides (**2c**) as well as to various dimeric derivatives (**2d, 2e**) (Fig. 2*D* and *SI Appendix*, Fig. S4*C*). The flavonoid taxifolin (**3b**) was metabolized by *B. bassiana* to the 7-*O*-methylglucoside (**3c**), its epimer (**3c-epi**), and quercetin-7-*O*-methylglucoside (**3d**) (Fig. 2*E* and *SI Appendix*, Fig. S4*D*).

**Fig. 3.**
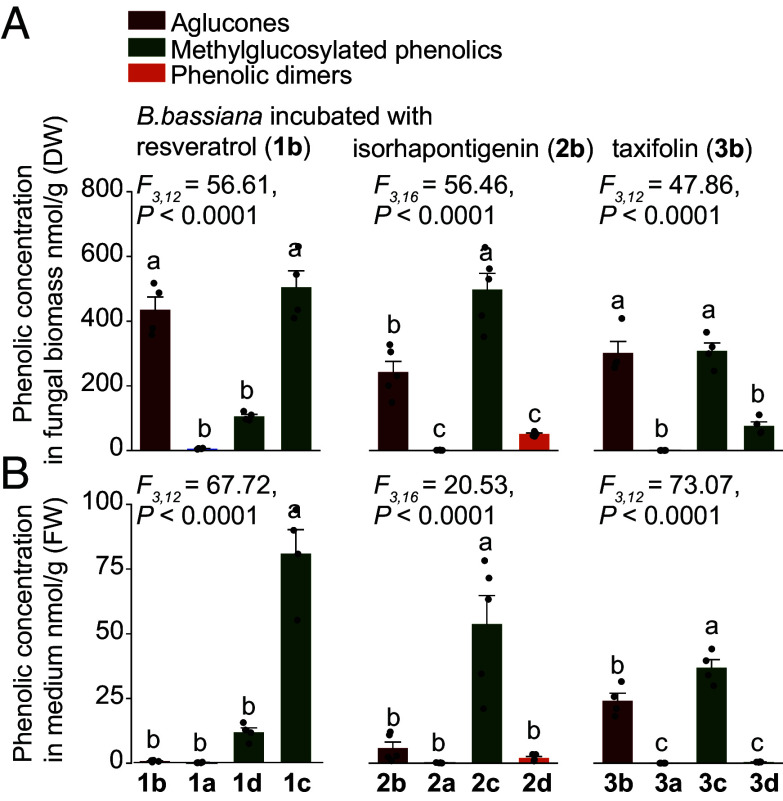
Methylglucosylated derivatives are the major metabolites produced by *B. bassiana* from the phenolic aglucones. Targeted analyses of phenolic aglucones and their metabolites in *B. bassiana* biomass (*A*) and incubation medium (*B*) after fungal incubation with the aglucones on PDA plates (*n* = 5). In all cases, the amounts of *E*- and *Z*-isomers were combined, e.g., compound 1a represents the combined quantity of 1a-*E* and 1a-*Z*. Statistically significant differences between means (±SE) were determined using one-way ANOVA with Tukey’s HSD tests in *A* and *B*. Different lowercase letters denote statistically significant differences (*P* < 0.05).

Purification of the *B. bassiana* metabolites allowed targeted liquid chromatography-mass spectrometry (LC–MS/MS), which revealed that the methylglucosylated resveratrols (**1c** and **1d**), isorhapontigenin (**2c**), and taxifolin (**3c**) were the major metabolites produced by the fungus from the phenolic aglucones, **1b**, **2b**, and **3b**, respectively (Fig. 3*A* and *SI Appendix*, Fig. S4*E*). The methylglucosides also predominated in the medium of *B. bassiana* after incubation with the aglucones (Fig. 3*B* and *SI Appendix*, Fig. S4*F*). These results indicate that *B. bassiana* can absorb phenolic aglucones from its growth medium, convert them to their corresponding methylglucosides in high yield, and then excrete these metabolites back into the medium.

### Phenolic Methylglucosides Are Stable and Nontoxic to *B. bassiana*.

**Fig. 4.**
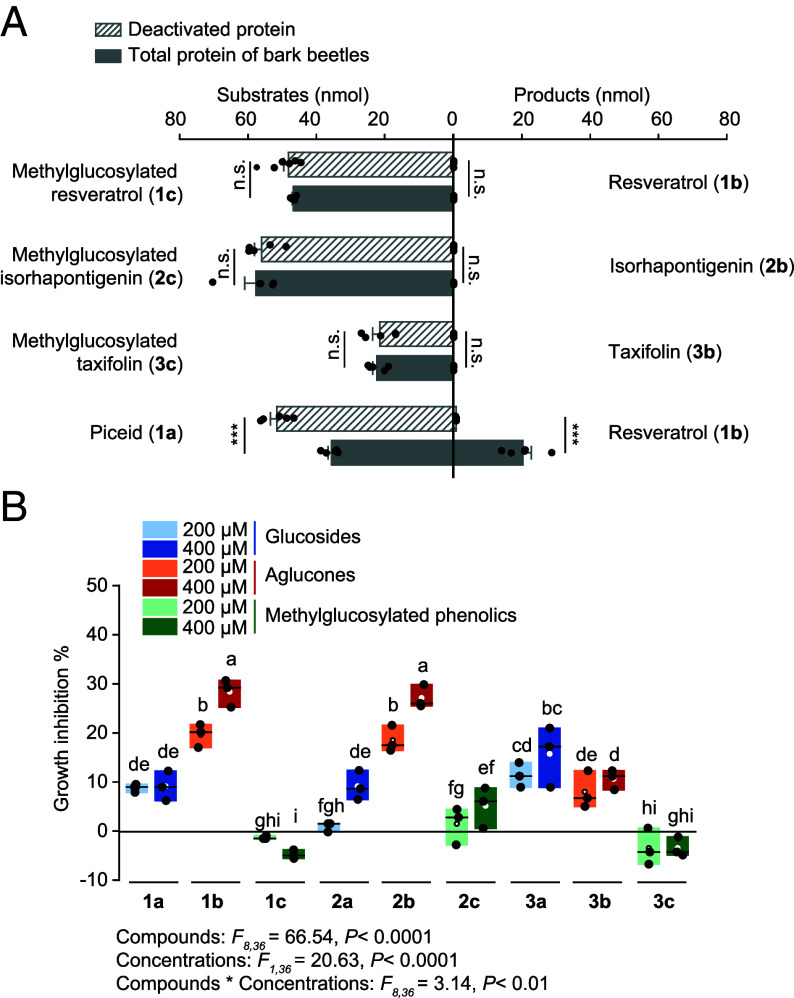
Methylglucosylated phenolics are not hydrolyzed by bark beetle enzymes and not toxic to the fungus *B. bassiana*. (*A*) Incubation of crude protein extract of bark beetles with methylglucosylated phenolic substrates in vitro, showing the lack of production of the corresponding aglucones. Boiled extracts served as negative controls, while the simple *O*-glucoside piceid was used as a positive control (*n* = 5). (*B*) Growth inhibition (%) of *B. bassiana* grown on PDA plates with additions of varying concentrations of glucosides, aglucones, and methylglucosylated phenolics, normalized to control treatments without phenolics (*n* = 3). Box plots represent the range of 25 to 75%, the middle lines indicate the median values, and the whiskers indicate the range of data points up to 1 time the interquartile range. Significant differences between means (±SE) were determined using two-tailed *t* tests in *A*, and two-way ANOVA followed by Fisher’s LSD tests in *B*. Different lowercase letters (*P* < 0.05) or asterisks (n.s., *P* ≥ 0.01; ***P < 0.001) denote statistically significant differences.

To assess the metabolic stability of the *B. bassiana*-derived phenolic methylglucosides for their bark beetle hosts, these were incubated in vitro with an *I. typographus* protein extract. However, methylglucosides were not hydrolyzed like the glycosylated phenolics (Fig. 4*A*), and were also not hydrolyzed by commercial β-glucosidases (*SI Appendix*, Fig. S5), indicating the increased metabolic stability of the *B. bassiana*-derived methylglucosides.

To determine whether the formation of methylglucosides by *B. bassiana* is a genuine detoxification process, we measured the growth of the fungus in the presence of the original spruce phenolic glucosides, the bark-beetle produced aglucones, and the fungal-produced methylglucosylated derivatives, relative to a control group. Phenolic glucosides caused only mild inhibition of *B. bassiana* growth, with approximately 10% reduction observed at 400 µM. This amount is in the natural range of concentration in the bark, and thus may also be an accurate concentration for the beetle (Fig. 4*B* and *SI Appendix*, Fig. S6). In contrast, the phenolic aglucones significantly suppressed fungal development, particularly the stilbenes **1b** and **2b**, resulting in nearly 30% growth inhibition at 400 µM. However, the methylglucosylated phenolic products formed by *B. bassiana* had no inhibitory effect on fungal growth. In fact, *B. bassiana* even displayed enhanced growth in the presence of **1c** at 400 µM (Fig. 4*B*). These findings indicate that the methylglucosylated phenolics are nontoxic to *B. bassiana* and that methylglucosylation enables the fungus to neutralize these plant-derived toxins, which are made even more toxic by bark beetle metabolism.

### A Specific UDP-Glycosyltransferase and *O*-Methyltransferase Are Involved in Methylglucoside formation by *B. bassiana*.

**Fig. 5.**
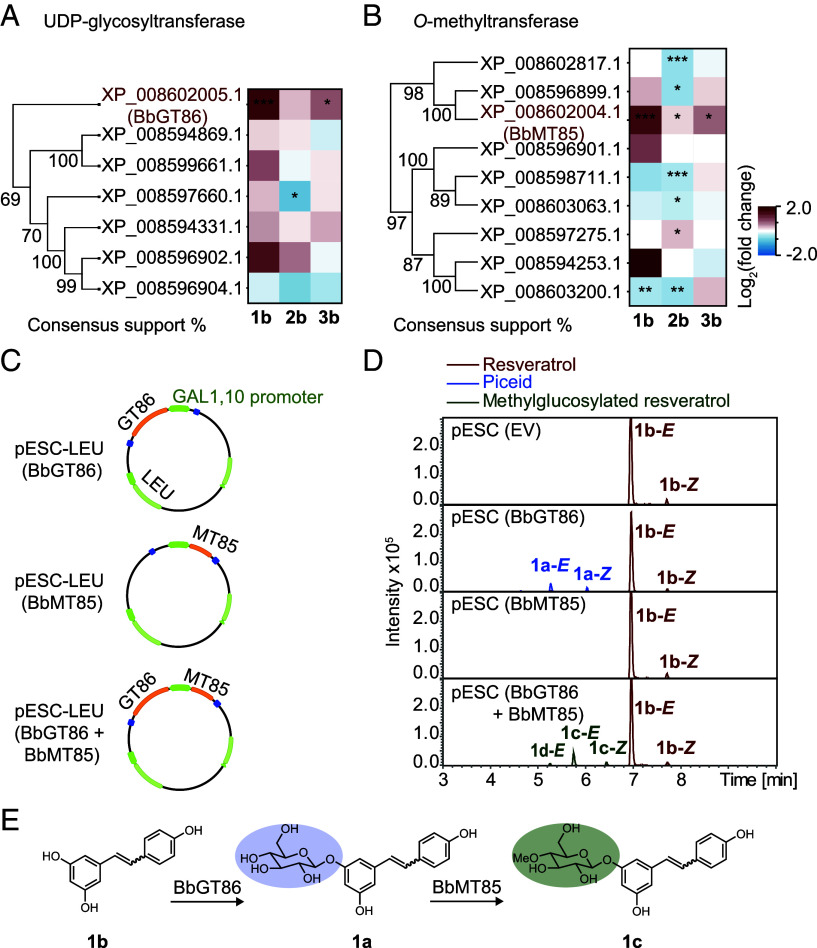
Identification of *B. bassiana* UDP-glycosyltransferase and *O*-methyltransferase genes involved in methylglucosylation by their induction with phenolic aglucones and their enzymatic activities. (*A* and *B*) Phylogenetic analysis of seven *B. bassiana* GTs (*A*) and nine *B. bassiana* MTs (*B*). Amino acid sequences were aligned and UPGMA trees were generated. Branch labels represent the consensus support (%), and protein accession numbers are listed. Heatmaps show the expression of genes in *B. bassiana* (relative to actin gene, *n* = 5) upon phenolic aglucone treatment. Data represent log_2_ fold-change in gene expression levels of *B. bassiana* incubated with aglucones, compared to controls. (*C*) Recombinant BbGT86, BbMT85, and the combination of BbGT86 and BbMT85 were expressed in a yeast system. (*D*) Metabolites of the phenolic aglucone resveratrol produced by the recombinant proteins. Depicted are extracted ion chromatograms in negative ionization mode of metabolites from yeast cells expressing empty vector (EV), recombinant BbGT86, BbMT85, or both BbGT86 and BbMT85 measured by UHPLC-qTOF-MS. Mass spectra of fungal products and standards are available in Edmond. **1b-*****E*** indicates the *E*-isomer and **1b-*Z*** indicates the *Z*-isomer. (*E*) An overview of the methylglucosylation pathway by which phenolic aglucones are metabolized by *B. bassiana*. Statistically significant differences between means (±SE) were determined by two-tailed *t* tests in *A* and *B*, indicated by asterisks (*P < 0.05; **P < 0.01; ***P < 0.001).

The *B. bassiana* genes encoding the enzymes involved in methylglucosylation were sought in analogy with previous research on this fungus (27). For the seven annotated UDP-glycosyltransferases and the nine annotated *O*-methyltransferases in the genome, expression was analyzed by qPCR with the fungus incubated on PDA plates supplemented with the phenolic aglucones **1b**, **2b,** and **3b**. Transcript levels of several of these genes were upregulated, especially by the stilbene aglucone resveratrol (**1b**) (Fig. 5 *A* and *B*). Our attention focused on two of these genes, the glycosyltransferase *Bbgt86* and the *O*-methyltransferase *Bbmt85*, which are located on the same genomic scaffold and thus might belong to the same pathway (*SI Appendix*, Fig. S7*A*). Structural modeling using AlphaFold 3 predicted a potential protein–protein interaction between BbGT86 and BbMT85, and so we continued with our analyses on these candidates to learn if they were part of the methylglucosylation pathway for phenolics (*SI Appendix*, Fig. S7*B*).

To determine the enzymatic functions of BbGT86 and BbMT85, the proteins were heterologously expressed in yeast both alone and together (Fig. 5*C*). Upon incubation with resveratrol (**1b**), methylglucosylated resveratrol conjugated at both positions 3 and 12 (**1c, 1d**, and both *E*- and *Z*-isomers) was detected in yeast cells coexpressing BbGT86 and BbMT85, but not in cells expressing either enzyme alone (Fig. 5*D*). Yeast cells expressing BbGT86 alone formed the phenolic glucoside **1a** when incubated with resveratrol, confirming its glycosyltransferase activity (Fig. 5*D*). Similarly, methylglucosylated isorhapontigenin (**2c**) and methylglucosylated taxifolin (**3c**) were produced in yeast expressing both BbGT86 and BbMT85 following incubation with **2b** or **3b**, respectively (*SI Appendix*, Fig. S7 *C* and *D*). In contrast, no methylglucosylated phenolics were detected when yeast expressing only BbMT85 was incubated with aglucones (Fig. 5*D* and *SI Appendix*, Fig. S7 *C* and *D*). However, methylglucosylated resveratrol (**1c**) was detectable when BbMT85-expressing yeast was incubated with the glucoside **1a** (*SI Appendix*, Fig. S7*E*). These results indicate that BbGT86 catalyzes the *O*-glycosylation of phenolic aglucones while BbMT85 subsequently methylates the hydroxyl group at the 4-position of the attached glucose moiety (Fig. 5*E* and *SI Appendix*, Fig. S7 *F*–*H*).

**Fig. 6.**
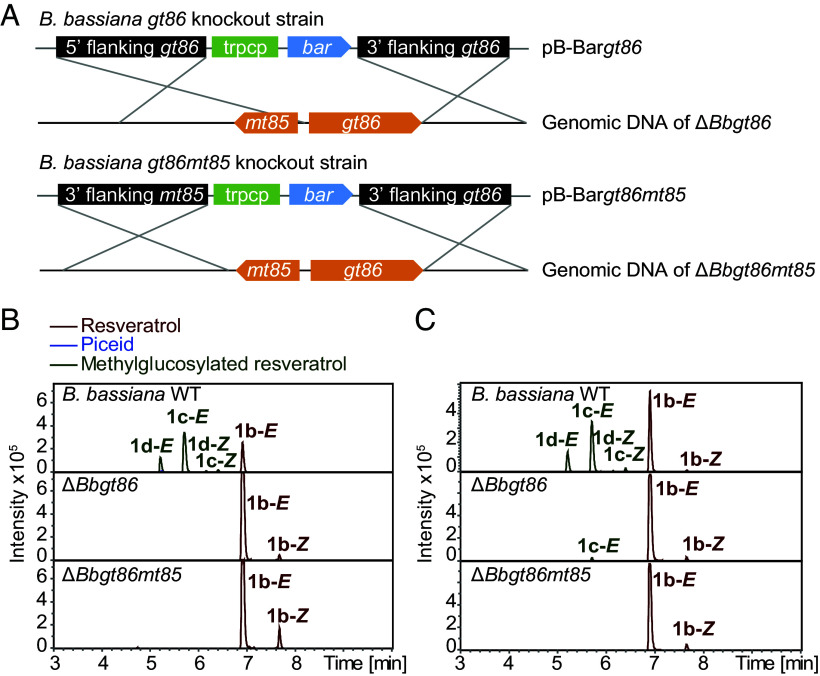
Knockout of the *B. bassiana gt86* gene and double knockout of the *gt86mt85* genes block the pathway of phenolic methylglucosylation. (*A*) Schematic of the constructed binary vectors used for targeted knockout of the *gt86* and *gt86mt85* genes from *B. bassiana* via homologous recombination. The binary vectors included cassettes with the 5′ flanking region, trpC promoter, *bar* resistance gene, and 3′ flanking region. (*B* and *C*) Metabolites of the knockout and control strains incubated with a stilbene aglucone and glucoside. Depicted are extracted ion chromatograms in negative ionization mode measured by UHPLC-qTOF-MS. The *B. bassiana* wild-type strain (WT), as well as Δ*Bbgt86* and Δ*Bbgt86mt85* mutants were incubated with the aglucone resveratrol (*B*) and the glucoside piceid (*C*). No piceid was detectable in samples. Mass spectra of fungal products and standards are available in Edmond. **1b-*E*** indicates the *E*-isomer and **1b-*Z*** indicates the *Z*-isomer.

To determine the in vivo function of BbGT86 and BbMT85 in *B. bassiana*, the genes were disrupted by homologous recombination using *Agrobacterium*-mediated transformation to generate loss-of-function mutants (Fig. 6*A* and *SI Appendix*, Fig. S8 *A* and *B*). The Δ*Bbgt86* and Δ*Bbgt86mt85* mutants plus the wild-type strain were then incubated with resveratrol (**1b**) or its glucoside piceid (**1a**). The methylglucosylated resveratrols **1c** and **1d** (both *E*- and *Z*-isomers) were detected in the wild-type strain but were absent in the Δ*Bbgt86mt85* mutant (Fig. 6 *B* and *C*). In the Δ*Bbgt86* mutant, **1c** was detected after incubation with the glucoside **1a**, reflecting the retained methyltransferase activity of BbMT85, but its abundance was markedly lower than in the wild-type strain (Fig. 6*C*). The Δ*Bbgt86* and Δ*Bbgt86mt85* mutants were also unable to convert the phenolic aglucones **2b** and **3b** into the corresponding methylglucosylated products **2c** and **3c** (*SI Appendix*, Fig. S8 *C* and *D*). These results demonstrate that disruption of either *gt86* or *gt86mt85* effectively suppresses the methylglucosylation pathway in *B. bassiana*, highlighting the roles of the encoded enzymes in the biosynthesis of methylglucosylated phenolic metabolites.

### Knockout of UDP-Glycosyltransferase and *O*-Methyltransferase Genes Reduces *B. bassiana* Growth.

**Fig. 7.**
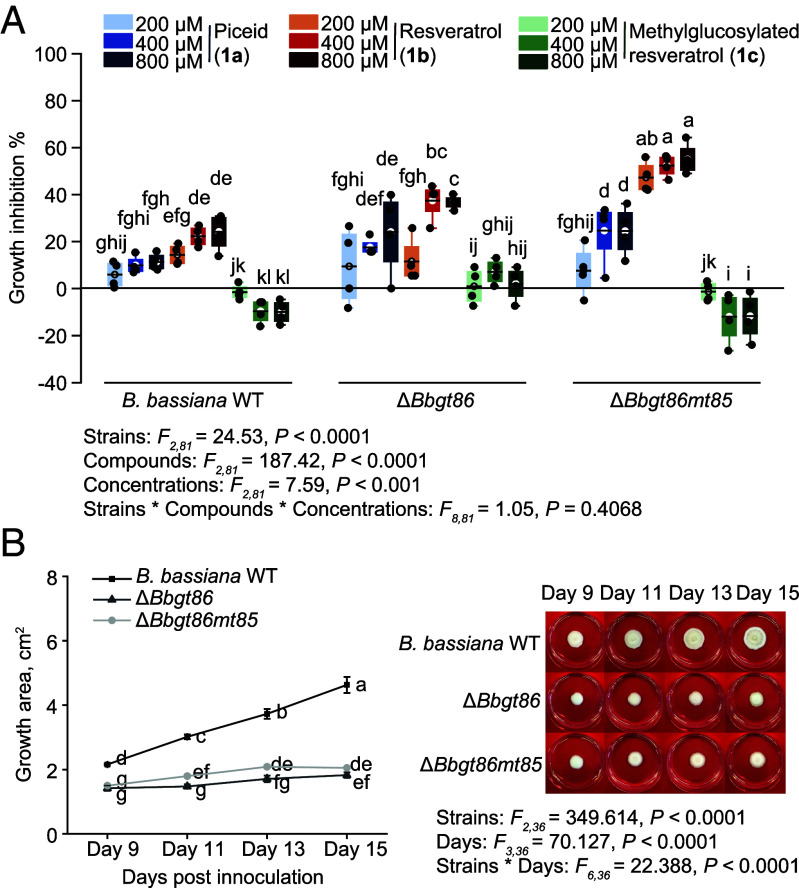
Knockout of the *B. bassiana gt86* gene and double knockout of the *gt86mt85* genes impact fungal growth on medium with stilbenes. (*A*) Growth inhibition (%) of Δ*Bbgt86* and Δ*Bbgt86mt85* mutants plus wild-type (WT) control on medium with added resveratrol, piceid, and methylglucosylated resveratrol at the concentrations of 200 µM, 400 µM, and 800 µM measured on day 9 postinoculation, relative to the control (*n* = 4). Box plots represent the range of 25 to 75%, the middle lines indicate the median values, and the whiskers indicate the range of data points up to 1 time the interquartile range. (*B*) Comparison of the growth of Δ*Bbgt86* and Δ*Bbgt86mt85* mutants plus wild-type (WT) control on PDA medium without stilbenes, measured from day 9 to day 15 postinoculation (*n* = 4). Significant differences between means (±SE) were determined using three-way ANOVA followed by Fisher’s LSD tests in *A*, and two-way ANOVA followed by Fisher’s LSD tests in *B*. Different lowercase letters denote statistically significant differences (*P* < 0.05).

The role of BbGT86 and BbMT85 in the resistance of *B. bassiana* to growth inhibition by stilbenes was assessed by culture on PDA plates. The presence of the stilbene aglucone resveratrol (**1b**) in the growth medium inhibited the development of the Δ*Bbgt86* and Δ*Bbgt86mt85* mutants and the wild-type strain, but this inhibition was significantly more pronounced in the mutants, particularly at the 400 µM and 800 µM concentrations (Fig. 7*A*). The stilbene glucoside piceid (**1a**) also suppressed the growth of the knockout mutants, though to a lesser extent. Importantly, the methylglucosylated resveratrol **1c** had no negative effect on the growth of either the *B. bassiana* WT or the mutant strains (Fig. 7*A*). Curiously, on medium without stilbenes the Δ*Bbgt86* and Δ*Bbgt86mt85* mutants exhibited markedly slower growth compared to the wild-type strain (Fig. 7*B*), suggesting that the encoded enzymes may have important roles besides phenolic metabolism. Based on metabolic profiling, the mutants exhibited reduced formation of other metabolites compared to the wild-type strain (*SI Appendix*, Fig. S9 *A* and *B*). Collectively, these results demonstrate that loss of BbGT86 function disrupts the methylglucosylation-mediated detoxification pathway in *B. bassiana*, leading to reduced growth in the presence of phenolics, as well as other changes in metabolism.

### Knockout of the UDP-Glycosyltransferase and *O*-Methyltransferase Genes Reduces *B. bassiana* Virulence to Bark Beetles.

**Fig. 8.**
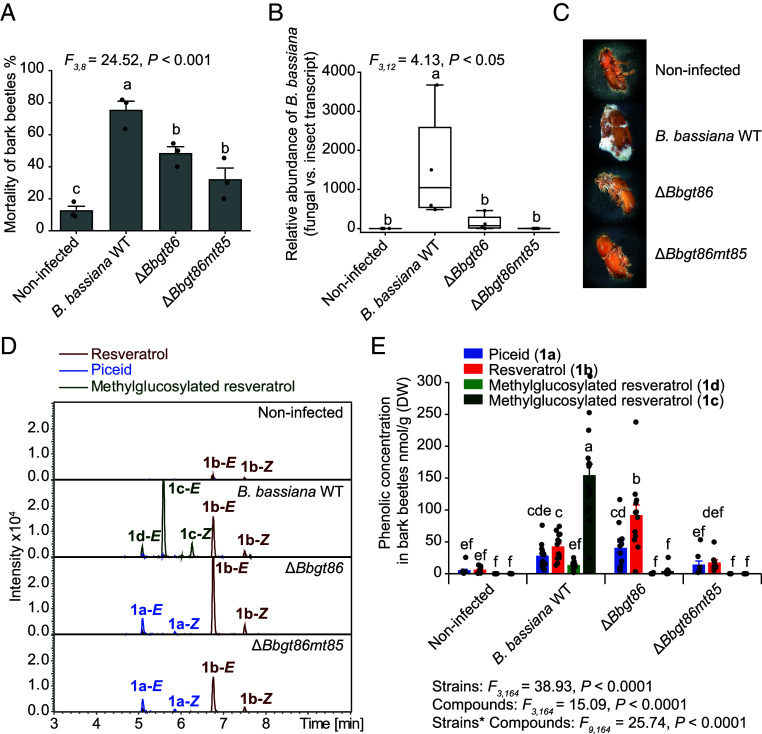
Knockout of the *B. bassiana gt86* gene and double knockout of the *gt86mt85* genes reduce bark beetle mortality. (*A*) Mortality rates of *B. bassiana*–infected Δ*Bbgt86* and Δ*Bbgt86mt85* mutants, and beetles infected with the WT, compared to an uninfected control fed on a semiartificial diet with Norway spruce phenolics (*n* = 3). (*B*) Relative abundance of *B. bassiana* in infected and uninfected bark beetles, measured as the transcript level of the *B. bassiana actin* gene relative to the bark beetle *rps3-α* gene (*n* = 4). Box plots represent the range of 25 to 75%, the middle lines indicate the median values, and the whiskers indicate the range of data points up to 1 time the interquartile range. (*C*) Photographs of uninfected beetles and those infected with Δ*Bbgt86*, and Δ*Bbgt86mt85* mutants and WT fungus, taken on the 10th day post fungal inoculation. (*D*) Metabolism of uninfected bark beetles and those infected with knockout and control strains. Depicted are extracted ion chromatograms in negative ionization mode measured by UHPLC-qTOF-MS. **1b-*E*** indicates the *E*-isomer and **1b-*Z*** indicates the *Z*-isomer. (*E*) Targeted analyses of the stilbene glucoside piceid and its aglucone and methylglucosylated derivatives in noninfected beetles and those infected with Δ*Bbgt86*, and Δ*Bbgt86mt85* mutants and the WT fungus fed on semiartificial diet (*n* = 10, 12, 8, and 15, respectively). Statistically significant differences between means (±SE) were determined using one-way ANOVA followed by Fisher’s LSD tests in *A* and *B*, and two-way ANOVA followed by Fisher’s LSD tests in *E*. Different lowercase letters denote statistically significant differences (*P* < 0.05).

To explore the ecological significance of the methylglucosylation pathway in allowing *B. bassiana* to infect host beetles feeding on spruce phloem containing phenolic compounds, we inoculated callow adult *I. typographus* bark beetles with the Δ*Bbgt86* and Δ*Bbgt86mt85* mutants plus the wild-type (WT) control. Mortality of bark beetles increased significantly following infection with *B. bassiana* compared to uninfected controls (Fig. 8*A*). However, loss of methylglucosylation capacity in the Δ*Bbgt86* and Δ*Bbgt86mt85* mutants resulted in approximately 30% lower mortality among infected beetles relative to those infected with the *B. bassiana* wild-type strain (Fig. 8*A*). Furthermore, the relative fungal abundance within beetles infected by the Δ*Bbgt86* and Δ*Bbgt86mt85* mutant strains was significantly lower than in those infected by the *B. bassiana* wild-type (Fig. 8*B*). Visual inspection confirmed reduced fungal colonization on the surface of beetles infected with Δ*Bbgt86* and Δ*Bbgt86mt85* compared to individuals infected with the wild-type fungus (Fig. 8*C*). Nontargeted and targeted metabolomic analyses revealed that the methylglucosylated resveratrols, **1c** and **1d,** were present in beetles infected with the *B. bassiana* wild-type strain, but undetectable in those infected with the Δ*Bbgt86* and Δ*Bbgt86mt85* mutant strains (Fig. 8 *D* and *E*). Similarly, methylglucosylated taxifolin **3c** was detected in wild-type-infected beetles but absent in those infected with the mutants (*SI Appendix*, Fig. S10 *A* and *C*). Interestingly, although **2a** is present in higher abundance than **1a** in intact spruce bark (Fig. 1*B*), its derivatives were nearly undetectable in all infected beetles (*SI Appendix*, Fig. S10*B*). Additionally, metabolomic profiling showed significant differences between beetles infected with the Δ*Bbgt86* and Δ*Bbgt86mt85* mutants and the wild-type *B. bassiana* (*SI Appendix*, Fig. S9*C*). The loss of glycosylation and methylation functions not only abolished the formation of methylglucosylated phenolics but also reduced levels of other phenolic glucosides in both the mutant fungi and their beetle hosts (*SI Appendix*, Fig. S9 *B* and *D*). These findings demonstrate that disruption of the methylglucosylation pathway through deletion of *gt86* or both *gt86* and *mt85*, compromises the ability of *B. bassiana* to metabolize host-derived phenolics, resulting in reduced fungal virulence and increased bark beetle survival.

## Discussion

Once plant chemical defense compounds have been ingested by herbivores, they may have a myriad of effects on other organisms. Compounds with antimicrobial activity can influence insect susceptibility to microbial pathogens, including viruses, bacteria, protozoa, and fungi. This area has been well reviewed recently, but the trends noted are often system-specific and the mechanistic bases are not always clear (6, 8). Here we demonstrated that the abundant antimicrobial phenolic glucosides present in the phloem of Norway spruce (*Picea abies*) are ingested by Eurasian spruce bark beetles (*Ips typographus*) and hydrolyzed (Fig. 1). These glucosides include stilbenes, such as piceid and isorhapontin, and flavonoids like taxifolin-3’-*O*-glucoside. The resulting aglucones were found to have even greater antimicrobial activity than their glucoside precursors (Fig. 4*B*). There are other examples where insect herbivores activate plant antimicrobial defenses obtained from their diet. For example, the cabbage aphid sequesters glucosinolates from their Brassicales host plants that it activates by hydrolysis to toxic isothiocyanates when attacked by *B. bassiana* (30). The western corn rootworm ingests benzoxazinoid glucosides from its maize host plant and hydrolyzes them upon attack. The products are toxic to nematodes and their symbiotic entomopathogenic bacteria (31). Although many more examples need to be studied, insect herbivores may indeed benefit from plant metabolites in resistance to pathogens, but the process depends strongly on diet choice and subsequent metabolism.

In this study, the phenolic glucosides and aglucones ingested by the bark beetle *I. typographus* and their metabolites did not inhibit the growth of the entomopathogenic fungus *B. bassiana*. This well-known arthropod parasite has been investigated for its potential to reduce bark beetle population outbreaks. We found that the fungus converts phenolic aglucones derived from spruce glucosides to methylglucosylated derivatives that are not toxic to the fungus (Fig. 4*B*) and are resistant to rehydrolysis by the bark beetles and other standard β-glucosidases (Fig. 4*A* and *SI Appendix*, Fig. S5). For this conversion, *B. bassiana* employs a two-step pathway consisting of a UDP-glycosyltransferase followed by an *O*-methyltransferase (Fig. 5). Mutant strains knocked out in the genes encoding these enzymes cannot produce methylglucosides (Fig. 6), and so grew more slowly on medium with stilbene aglucones than the wild-type strain (Fig. 7) and were less pathogenic to bark beetles (Fig. 8).

Methylglucosylation is known in other fungi that, like *B. bassiana*, are also parasites of insects and also from the order Hypocreales. Glycosyltransferase–methyltransferase pairs were characterized from *Metarhizium robertsii*, *Isaria fumosorosea,* and *Cordyceps militaris* that produce methylglucose conjugates of stilbenes, flavonoids, and other phenolic natural products including anthraquinones and benzenediol lactones (27). The occurrence of methylglucosylation in other entomopathogenic fungi suggests that this conjugation may be a common way to allow growth while infecting insects that feed on a phenolic-rich diet. Like methylglucose, fungi produce other phenolic conjugates with less typical sugars, and so gain the advantages of glycosylation while resisting rehydrolysis by ubiquitous β-glucosidases. For instance, the rice sheath blight fungus, *Rhizoctonia solani*, detoxifies the rice flavonoid sakuranetin via xylosylation (26), while the bracket fungus *Daldinia eschscholzii* detoxifies the stilbene resveratrol by ribosylation (32). There are also many other fungal UDP-glycosyltransferases described that conjugate phenolics with a simple glucose residue. These typically accept many different acceptors as substrates, but have high regio- and stereo specificity (33, 34). Among the substrates glycosylated are other plant defense compounds, such as indole derivatives (35, 36).

The *B. bassiana* UDP-glycosyltransferase (BbGT86) involved in methylglucosylation of stilbenes and flavonoids in *B. bassiana* was also found to accept other natural products as substrates in in vitro assays (27). If this enzyme also glycosylates other acceptors in vivo, this could explain why the BbGT86 knockout strain grew more slowly than the wild-type even on medium lacking phenolic compounds to detoxify. Glycosyltransferases are involved in a wide range of basic processes in fungi, such as synthesis of the cell wall and glycosylation of proteins, lipids, and sterols (37–39), which if knocked out could impair growth. Interestingly, the genes encoding the glycosyltransferase and *O*-methyltransferase pair in *B. bassiana* and other Hypocreales are typically found in gene clusters (27) as are other genes involved in phenolic degradation (40). Such clusters may promote the evolutionary transmission of their constituent genes as well as their coordinated transcription so that the methylglucoside product is formed rather than the initial, more readily cleaved glucoside. Besides direct detoxification, fungi such as *B. bassiana* could have other mechanisms of resisting plant defense compounds. Efflux transporters from the ABC family are known to excrete compounds like resveratrol from fungal cells, and so could also play a role in increasing fungal growth and virulence on substrates containing toxic compounds (41, 42).

The capacity of the entomopathogen *B. bassiana* to metabolize the phenolic aglucones released by *I. typographus* bark beetles from Norway spruce phenolic glucosides may be crucial for its ability to infect host insects with a diet like that of *I. typographus*. Knocking out the methylglucosylation pathway of this fungus reduced its growth in bark beetles and decreased its virulence. Entomopathogenic fungi infecting herbivorous insects had not previously been described to detoxify the plant defense compounds ingested by their hosts. Earlier work on *B. bassiana* showed that the infectivity of this fungus on insect herbivores varies with the plant that the insect is feeding on, implicating plant chemistry as a factor in its success (30). For example, the glucosinolate hydrolysis product, allyl isothiocyanate, from wasabi leaves (43) and the glycoalkaloids solanine and tomatine from tomato, potato, and other Solanaceae (44) inhibit the growth of *B. bassiana*. Thus, the fungus may have difficulties in infecting insect herbivores on these host plants. In contrast, *B. bassiana*’s ability to metabolize the phenolic compounds of spruce, as shown here, enables it to successfully infect bark beetles attacking spruce trees. Given the arsenal of glycosyltransferases, glutathione-*S*-transferases and other detoxification genes in its genome (28, 30, 45), *B. bassiana* can be expected to effectively metabolize other plant defenses as well. Reports of detoxification genes from other entomopathogenic fungi (27) suggest the broad ability of these insect parasites to handle plant defenses, which may be crucial to their virulence on herbivorous insects.

Insect herbivores have long been known to accumulate plant defense metabolites from their diet as defenses against their own enemies (2). Moreover, recent research has documented examples of insects ingesting specific antimicrobial compounds to minimize pathogen infection (6). However, as shown here for *B. bassiana*, fungal pathogens are able to circumvent the toxicity of these dietary defenses and cause disease. Similar abilities are found in entomopathogenic nematodes, which have developed, for example, both behavioral and metabolic resistance to the benzoxazinoid defenses sequestered by a lepidopteran larva feeding on maize (46).

Concerning *B. bassiana*, our results provide a biochemical basis for its use in controlling bark beetle attacks on Norway spruce and other conifer hosts (47, 48), whose bark is typically rich in phenolic compounds (49, 50). Surveys of *B. bassiana* strains show that these often vary in their virulence to bark beetles (16, 51, 52), which may be due to strain-specific differences in the rate of phenolic detoxification. Strains of *B. bassiana* are also reported to vary in their susceptibility to another class of conifer chemical defenses, the monoterpenes of the resin (16, 53). Future efforts to improve *B. bassiana* for the control of conifer bark beetles should select strains for their ability to methylglucosylate host tree phenolic compounds, as well as to resist monoterpenes. Such differences among strains are reminiscent of the geographic mosaic theory of coevolution where, within a species, local populations may be differentially selected for resistance to a pathogen or for virulence on a host depending on particular biotic and abiotic factors of the environment (54). Populations of *B. bassiana* that rarely encounter bark beetles or other insects that feed on high doses of antimicrobial phenolic substances may not be under strong selection pressure for the ability to detoxify these substances, which may incur substantial metabolic and ecological costs. Further research is needed to correlate *B. bassiana* resistance and phenolic detoxication across a broad geographical range to learn more about the evolution of detoxification in this entomopathogen.

## Methods

### Bark Beetle Rearing and Entomopathogenic Fungi Isolation.

A continuous rearing of Eurasian spruce bark beetles (*Ips typographus*) was established in the laboratory in the summer of 2021 (55), with detailed procedures described in the *SI Appendix, Methods*.

*Beauveria bassiana* strains 01 and 02 were isolated from bark beetles infected with the entomopathogen that were caught in the wild and inadvertently added to the laboratory colony. The fungal isolates were incubated on PDA plates at 25 °C. Genomic DNA was extracted using the DNeasy Blood & Tissue Kit (Qiagen, Hilden, Germany). To identify the fungal strains, the internal transcribed spacer (ITS) regions were cloned and sequenced (56). The primer pairs used for ITS cloning are listed in *SI Appendix*, Table S3. Phylogenetic analysis of the ITS regions from the isolated *B. bassiana* strains were compared to seven additional strains, as detailed in the *SI Appendix, Methods*. The ITS sequences for isolated *B. bassiana* strains are listed in the *SI Appendix*, Table S4.

### Culture of *B. bassiana*–Infected Bark Beetles Fed on a Spruce Bark Diet.

*B. bassiana* conidia were collected and adjusted to 10^8^ conidia per mL for the experiment, and spruce bark diet was freshly prepared as described in the *SI Appendix, Methods*. For supplementation with resveratrol, 20 µmol/g resveratrol was added to the spruce bark diet. To infect bark beetles, callow adults were washed sequentially with MilliQ water for 30 s, 70% ethanol for 20 s, and MilliQ water for another 30 s, and then completely immersed in conidial suspensions of *B. bassiana* for 30 s to ensure that their bodies were thoroughly covered with fungal conidia. Callow adults washed without exposure to fungal conidia served as the uninfected control group. Each beetle was placed individually in a tube containing the spruce bark diet and sealed with parafilm. All beetles were reared at 25 °C for 10 d. The mortality of the bark beetles was recorded for each treatment. Bark beetles and semiartificial diet from each treatment were collected. The metabolites of bark beetles and semiartificial diet from each treatment were extracted and further analyzed by UHPLC-qTOF-MS.

### Phenolic Content in Spruce Bark and Bark Beetles.

To quantify the content of phenolic compounds in Norway spruce bark and bark beetles, chemical analyses were performed on bark powder, different life stages of bark beetles, and beetle frass. The sample collections and compound extractions are described in detail in the *SI Appendix, Methods*. Phenolic compounds were analyzed using LC–MS/MS.

### Deglycosylation Assays.

For the deglycosylation enzyme assay, protein from each sample was incubated with phenolic glucosides or methylglucosylated phenolic compounds in phosphate buffer (50 mM, pH 6.4) at 28 °C for 2 h. A boiled protein sample was used as a negative control. The protein extraction procedures and detailed enzyme assays are described in the *SI Appendix, Methods*. Additionally, the α/β-glucosidase inhibitor, castanospermine, was used to test whether bark beetle pupal protein activity on phenolic glucoside was inhibited. Reactions were terminated by pure MeOH and later analyzed by LC–MS/MS for hydrolysis products.

### Metabolism of Phenolic Aglucones by *B. bassiana*.

*B. bassiana* strains were inoculated on PDA plates supplemented with 200 µM of phenolic hydrolysis products for 7 d at 25 °C. The stilbene aglucones were added as commercially available mixtures of the *E-* and *Z-* isomers with approximately 10 to 15% of the *Z*-form (The approximate proportion of *Z*-isomers found after bark beetle metabolism ranged from 5 to 25%). *B. bassiana* incubated in PDA with solvent was used as a negative control. The sample collections and extractions are detailed in the *SI Appendix, Methods*. Subsequently, nontargeted metabolomics analyses were performed using UHPLC-qTOF-MS and targeted analyses using LC–MS/MS.

### Nontargeted Metabolomics Analyses by UHPLC-qTOF-MS.

The extracts were analyzed via UHPLC (Thermo Dionex Ultimate 3000, MA, USA) coupled to an qTOF-MS system (Bruker timsTOF, Bremen, Germany) following previously described methods (57), with a detailed description given in the *SI Appendix, Methods*. HRMS analyses were performed in negative ionization mode and automatic MS2 scans (“autoMS”) enabled. Data were analyzed using the MetaboScape 5.3 software (Bruker, Bremen, Germany) and MetaboAnalyst 5.0 and 6.0 (https://dev.metaboanalyst.ca/). Additionally, features were analyzed using SIRIUS (version 5.8.6) to predict molecular formulas, chemical structures, and chemical taxonomy. The results of the analyses are listed in the Dataset S1.

### Targeted Chemical Analyses Using LC–MS/MS.

Targeted analyses of phenolic compounds were performed on an Agilent HP1260 series HPLC instrument (Agilent Technologies, Böblingen, Germany) coupled to an API5000 tandem mass spectrometer (Applied Biosystems, Darmstadt, Germany) or a Triple Quad 6500+ system (SCIEX, MA, USA) following previously described methods (57), with a detailed description given in the *SI Appendix, Methods*. Parameters are described in *SI Appendix*, Table S5. Analyst 1.5 software (Applied Biosystems) and MultiQuant 3.0.3 software (SCIEX) were used for data acquisition and processing. Quantification of individual compounds was achieved by external calibration curves, with the origins of the external standards listed in *SI Appendix*, Table S6.

### Identification of Phenolic Compounds by Purification and NMR Analyses.

The phenolic metabolites of spruce bark, bark beetles, and *B. bassiana* were identified by chromatographic and MS/MS comparison with commercially available standards (*SI Appendix*, Table S6) via UHPLC-qTOF-MS. When commercial standards were not available, the compounds were isolated from spruce bark or *B. bassiana* fungal cultures, as mentioned in the *SI Appendix, Methods*.

NMR measurements were carried out on a 700 MHz Bruker Avance III HD spectrometer (Bruker Biospin GmbH, Rheinstetten, Germany) and a 500 MHz Bruker Avance III HD spectrometer (Bruker Biospin GmbH, Rheinstetten, Germany) using standard pulse sequences as implemented in Bruker Topspin ver. 3.6.1, with a detailed description in the *SI Appendix, Methods*.

### Transcript Abundance of UDP-Glycosyltransferase and *O*-Methyltransferase Genes in *B. bassiana*.

To examine the inducibility of UDP-glycosyltransferase and *O*-methyltransferase encoding genes by phenolic aglucones, *B. bassiana* cultures were incubated in PDA with 200 µM or 400 µM of phenolic aglucones for 4 d at 25 °C. *B. bassiana* incubated in PDA with solvent alone was used as a negative control. Total RNA isolation and cDNA syntheses are described in the *SI Appendix, Methods*. Subsequently, qPCR was performed to determine the relative transcript abundance of *B. bassiana actin* using Biozym Blue S’Green qPCR Mix Separate ROX (Biozym, Steinbrinksweg, Germany). Primer pairs (designed using Genious prime version 2023.2.1) and gene accession numbers are listed in *SI Appendix*, Tables S3 and S4, respectively. All seven GT and nine MT protein sequences were searched in NCBI, based on the previously annotated *B. bassiana* ARSEF 2860 strain sequence (assembly accession: ASM28067v1). Phylogenetic analysis of GT and MT proteins was performed on the amino acid sequences using Clustal Omega 1.2.2, and a UPGMA tree was generated using Geneious Prime.

### Recombinant BbGT86 and BbMT85 Expression in Yeast.

To determine the specific activities of BbGT86 and BbMT85 with phenolic compounds, these proteins were heterologously expressed in yeast cells. The BbGT86 and BbMT85 encoding genes were cloned from the isolated *B. bassiana* strain and inserted in the pESC-Leu vector, resulting in pESC (BbGT86), pESC (BbMT85), and pESC (BbGT86 + BbMT85) vectors, with detailed descriptions in the *SI Appendix, Methods*. pESC (EV) without protein inserts was used as a negative control. The vectors were then transformed into **Saccharomyces cerevisiae* strain* INV*Sc*1 using the S.c. EasyComp Transformation Kit (Invitrogen). The procedures for protein induction in yeast cells are described in the *SI Appendix, Methods*. Subsequently, 10 mL yeast cultures were supplemented with 100 µM phenolic compounds for 24 h at 25 °C and 160 rpm for the enzyme assay. Reactions were terminated by pure MeOH and later analyzed by UHPLC-qTOF-MS.

### *B. bassiana* Gene Modification via *Agrobacterium tumefaciens*-Mediated Transformation (ATMT).

To construct homologous recombination cassettes targeting the replacement of *gt86* or both *mt85* and *gt86*, approximately 1 to 2 kb 5′ and 3′ flanking regions of the targeted genes were amplified from *B. bassiana* strain 01 genomic DNA. TrpC promoter and the Basta resistance gene (*bar*) were coloned from the pBarGPE1 (Bioactiva Diagnostica, Höhe, Germany) plasmid. pB-*Bargt86* and pB-*Bargt86mt85* vectors were constructed using binary pCAMBIA^XmnI^ vector as backbone containing cassettes of the 5′ flanking region:TrpC promoter: *bar* gene: 3′ flanking region (Fig. 6*A*). The detailed methods are described in *SI Appendix, Methods*.

The *B. bassiana mt85* and *gt86* homologous recombination vectors (pB-*Bargt86* and pB-*Bargt86mt85*) were electroporated into AGL-1 *Agrobacterium* electrocompetent cells (GoldBio, St Louis MO, USA) using a Gene Pulser (Bio-Rad, Hercules, CA) under standard conditions. ATMT was performed following a modified method from Moon et al. (58), detailed in the *SI Appendix, Methods*. To confirm successful gene knockout in the strains Δ*Bbgt86* and Δ*Bbgt86mt85*, genotyping and metabolomic analyzing were conducted as described in the *SI Appendix, Methods*. All experiments involving genetically modified *B. bassiana* strains were conducted in a biosafety level 2 (S2) laboratory at the Max Planck Institute for Chemical Ecology.

### Effects of Phenolic Compounds on *B. bassiana* Growth and Metabolites.

The growth of wild-type *B. bassiana* and mutants Δ*Bbgt86* and Δ*Bbgt86mt85* was monitored as described in the *SI Appendix, Methods*. Meanwhile, to assess the effect of the *gt86* or *gt86mt85* gene knockouts on *B. bassiana* development and phenolic metabolites, the wild-type strain and mutant strains were grown on PDA plates with varying concentrations of piceid, resveratrol, or methylglucosylated resveratrol, as described in the *SI Appendix, Methods*. Additionally, wild-type *B. bassiana*, Δ*Bbgt86,* and Δ*Bbgt86mt85* mutants were incubated on PDA plates supplemented with 400 µM resveratrol or a solvent control for 9 d at 25 °C. The metabolites of harvested fungal biomass were extracted and further analyzed using UHPLC-qTOF-MS.

### Effects of Phenolic Metabolism by *B. bassiana* on Successful Bark Beetle Infection.

To quantify the infection efficiency, callow bark beetles were infected with the isolated wild-type *B. bassiana* and mutant strains. The detailed procedures were described in the *SI Appendix, Methods*. Additionally, transcript abundance analysis was performed to assess the virulence of *B. bassiana* on bark beetle. Genomic DNA was extracted, and qPCR was conducted as described in the *SI Appendix, Methods*. The bark beetle *RPS3-a* (40S ribosomal protein S3-A) gene (59) was used as an internal control to normalize the abundance of *B. bassiana actin* gene transcripts. Primer pairs and gene accession numbers are listed in *SI Appendix*, Tables S3 and S4, respectively. Furthermore, bark beetles infected with wild-type *B. bassiana*, Δ*Bbgt86*, and Δ*Bbgt86mt85* mutants, as well as uninfected bark beetles were collected on the 10th day postinfection. Meanwhile, the diet without bark beetle rearing, and diet on which bark beetles were reared, as well as wild-type *B. bassiana* infected bark beetles were collected. Metabolites were extracted and analyzed using a UHPLC-qTOF-MS system and an LC–MS/MS system.

### Statistical Analyses.

Data were analyzed using Origin 2023, with figures generated in both Origin 2023 and Adobe Illustrator CS5. Statistical prerequisites, including homogeneity of variances and normality, were determined using Origin 2023. Heatmap figures were prepared using ChiPlot (https://www.chiplot.online). The detailed statistical analyses are given in the figure legends. In graphs, data were analyzed as mean ± SE. All data presented are representative of at least three independent experiments, as indicated in the figure legends, along with the number of biological replicates. Different letters on the graphs represent differences at *P* < 0.05, while asterisks denote significant difference levels: n.s, *P* ≥ 0.05; *, *P* < 0.05; **, *P* < 0.01; ***, *P* < 0.001.

## Supplementary Material

Appendix 01 (PDF)

Dataset S01 (XLSX)

## Data Availability

All raw data supporting the findings of this study are available through Edmond, 10.17617/3.8AHMHE (60) and Dataset S1. The accession numbers of rRNA or mRNA reported in this paper are available in the NCBI database [PV798168 (61), PV799956 (62), PV799292 (63), and PV799293 (64)].
