# Supplementary material for: Detoxification of conifer antimicrobial defenses promotes entomopathogenic fungus infection of bark beetles
Source: Proc Natl Acad Sci U S A. 2025 Dec 29;123(1):e2525513122. doi: 10.1073/pnas.2525513122 (PMC12773783; doi:10.1073/pnas.2525513122)
Supplement: Supplementary file 1 — Appendix 01 (PDF) [file pnas.2525513122.sapp.pdf]

Supporting Information for

Detoxification of conifer antimicrobial defenses promotes entomopathogenic fungus infection of bark beetles

Ruo Sun, Baoyu Hu, Yoko Nakamura, Michael Reichelt, Xingcong Jiang, Katrin Luck, Christian Paetz, Jonathan Gershenzon

Corresponding authors: Ruo Sun, Jonathan Gershenzon

Email: rsun@ice.mpg.de, gershenzon@ice.mpg.de

This PDF file includes:

Supporting text: Methods

Figures S1 to S10

Tables S1 to S6

SI References

Other supporting materials for this manuscript include the following:

Dataset S1 (separate file). the source data behind all graphs and tables in the paper

Supporting Information Text

Methods

Bark beetle rearing

A continuous rearing of Eurasian spruce bark beetles (*Ips typographus*, Coleoptera: Curculionidae), was established in the laboratory in the summer of 2021 (1). The initial beetle population was started with adult beetles captured from a state-owned forest near Jena, Germany (50°54'28.3"N 11°39'28.9"E) and supplemented with freshly-caught beetles once per year. The bark beetles were continuously reared on freshly cut Norway spruce (*Picea abies*) logs (approximately 28 cm in diameter and 25 cm in height) within a controlled environmental chamber set at 25°C, 60% relative humidity, and a 16:8 h light: dark photoperiod. A healthy Norway spruce tree was felled monthly from the location mentioned above, and the logs cut into 25 cm sections. The ends of the logs were coated in paraffin to prevent desiccation. Each log was placed individually in a plastic box with a metallic mesh on the lid for ventilation. A group of 40 adult beetles was introduced to each log, which was then kept in the controlled environmental chamber.

Phylogenetic analysis of the internal transcribed spacer (ITS) regions

Phylogenetic analysis of the ITS regions from *Beauveria bassiana* strain 01 and *B. bassiana* strain 02 were compared to seven additional strains. The nucleic acid sequences were aligned using Clustal Omega 1.2.2, and a UPGMA tree was generated using Geneious Prime, with branch labels indicating the consensus support (%). The ITS sequences for isolated *B. bassiana* strains are listed in the *SI Appendix*, Table S4.

Culture of *B. bassiana*-infected bark beetles fed on a spruce bark diet

B. bassiana conidia were collected from fungal cultures grown on PDA plates for 2 weeks at 25°C. The conidia were gently scraped off from the plates into sterile water (25 mL) in a Falcon tube. The conidial suspension was filtered through a 10 µm pore-size Versapor membrane filter (Pall Corporation, NY, USA) to obtain pure conidia. The concentration of conidia was calculated using a Neubauer hemocytometer (Paul Marienfeld, Lauda-Königshofen, Germany), and adjusted to 10^8 conidia per mL with milliQ water for the experiment.

To prepare the spruce bark diet, fresh bark from *Picea abies* was collected and processed (1). The bark was cut into small pieces, ground into a fine powder using a vibratory micro mill Pulverisette 0 (Fritsch GmbH, Idar-Oberstein, Germany) with liquid nitrogen, and then freeze-dried using an ALPHA 1-4 LDplus freeze dryer (Martin Christ, Osterode am Harz, Germany) for 4 days. The semi-artificial diet consisted of 30 g freeze-dried bark powder, 6.4 g yeast, 0.5 g sodium benzoate, 0.1g streptomycin, and 63 mL sterilized milliQ water for every 100 g of diet. For supplementation with resveratrol, 136.8 mg of resveratrol (equivalent to 20 µmol/g) was added to the same mixture. Approximately 15 g of the diet mixture was put into 5 mL tubes (57 X 15.3 mm), forming a 2 cm high layer. The diet was prepared under sterilized conditions. To infect bark beetles, callow adults (collected 25 days after infesting logs) were washed sequentially with

MilliQ water, 70% ethanol, and MilliQ water, and then exposed to conidial suspensions of *B. bassiana* (10^8 conidia per mL). Callow adults washed only with water and 70% ethanol, without exposure to fungal conidia served as the uninfected control group. Each beetle was placed individually in a tube containing the spruce bark diet and sealed with parafilm. Three replicates (around ten tubes in each replicate) were prepared for each treatment group, and all beetles were reared at 25°C for 10 days. The mortality of the bark beetles was recorded for each treatment. Bark beetles and semi-artificial diet from each treatment were collected and frozen in liquid nitrogen. The freeze-dried samples were weighted and extracted in 200 µL of 80% MeOH and analyzed by non-targeted metabolomics with an UHPLC-qTOF-MS system.

Phenolic content in spruce bark and bark beetles

To quantify the content of phenolic compounds in Norway spruce bark and in bark beetles, chemical analyses were performed on bark powder, different life stages of bark beetles (larvae, pupae, callow adults, and mature adults), and their frass. Bark powder was collected from freshly cut spruce logs as described above. Bark beetle larvae, pupae, and callow adults were collected at 18-, 20-, and 25-days post-infestation of logs, respectively, while mature adults were collected 30 days after infestation, following their emergence from the logs. For frass collection, approximately 10 bark beetles were washed in MilliQ water and then placed in Petri dishes for 24 hours. The collected frass was pooled into one tube as one sample. Each treatment had 4-5 biological replicates. All materials (bark powder, beetles, and frass) were immediately weighed and frozen in liquid nitrogen, and stored at -80°C until extraction. Samples were extracted by adding 200 µL of an 80% MeOH as the extraction solvent. Each sample was homogenized by shaking with 5-6 glass beads (2.85-3.3 mm diameter) for 10 minutes on a horizontal shaker. The supernatant was collected after centrifugation at 13,000 x g for 30 minutes at 4°C. The supernatant was then transferred into 0.3 mL vials, and analyzed for phenolic compounds using targeted liquid chromatography-mass spectrometry (LC-MS/MS).

To determine the isomer structures of the phenolic compounds in the spruce bark and bark beetles, non-targeted metabolomic analyses were performed using UHPLC-qTOF-MS. Analyses were conducted on intact spruce bark, bark beetle tunneled spruce bark, and adult bark beetles collected from infested logs. Spruce bark samples were collected from logs, immediately flash-frozen in liquid nitrogen, and wrapped in aluminum foil to protect them from light. Adult bark beetles and their frass were collected in Falcon tubes, also wrapped in aluminum foil. All samples were freeze-dried for three days prior to extraction. Approximately 500 mg of freeze-dried bark and 20 mg of beetles were extracted with 5 mL and 1 mL of pure MeOH, respectively. The extracts were analyzed immediately using UHPLC-qTOF-MS.

Protein extractions and deglycosylation assays

Bark beetle pupae, which have a high protein content and low levels of phenolic compounds, were collected 20 days post-infestation of spruce logs. The pupae were cleaned with sterilized MilliQ water and immediately frozen in liquid nitrogen. For each biological replicate (n = 5), five pupae were pooled into a 1.5 mL Eppendorf tube. Proteins from *B. bassiana* strain 01 were extracted from 2-week-old fungal cultures

(*n* = 5). Samples were extracted in 200 µL of ice-cold phosphate buffer (50 mM, 10% glycerol, pH 6.4) with ceramic beads using a homogenizer for 3 minutes. The homogenized samples were centrifuged at 13,000 x *g* at 4°C for 20 minutes, after which the clean supernatant was transferred to 1.5 ml Eppendorf tubes. Protein concentrations were determined using 5 µL of each sample with Quick Start Bradford 1× Dye Reagent (Bio-Rad, Hercules, CA) and bovine serum albumin (Thermo Fisher Scientific, Waltham, USA) as the standard.

For the deglycosylation enzyme assay, protein from each sample (10 µg of bark beetle pupae or 10 µg of *B. bassiana* protein) was incubated with 250 µM of phenolic glucosides or methylglucosylated phenolic compounds in 200 µL phosphate buffer (50 mM, pH 6.4) at 28°C for 2 hours. A boiled protein sample (95°C for 5 min) was used as a negative control to confirm the loss of enzyme activity. Additionally, the α/β-glucosidase inhibitor, castanospermine, was used at concentrations of 0 mM and 0.1 mM to test if bark beetle pupal protein activity on phenolic glucoside was inhibited. Enzyme assays were also conducted using commercial β-glucosidases from almonds and *Aspergillus niger* on piceid or methylglucosylated resveratrol. Each reaction contained 0.01 U of commercial glucosidase enzyme in a 200 µL reaction volume. Reactions were terminated by transferring 10 µL of the mixture into 190 µL of pure MeOH and freezing it at -20°C. After centrifugation (13,000 x g, 4°C, 30 min), the supernatants were transferred into 0.3 mL vials and analyzed by LC-MS/MS for hydrolysis products.

Metabolism of phenolic aglucones by *B. bassiana*

B. bassiana strains were inoculated on PDA plates supplemented with 200 µM of phenolic hydrolysis products dissolved in 0.025% aqueous DMSO and incubated for 7 days at 25°C, respectively. The stilbene aglucones were added as commercially available mixtures of the *E*- and *Z*- isomers with approximately 10-15% of the *Z*-form (The approximate proportion of *Z*-isomers found after bark beetle metabolism ranged from 5-25%.). *B. bassiana* incubated in PDA with 0.025% DMSO was used as a negative control. 4-5 replicates were used for each treatment. The harvested *B. bassiana* was freeze-dried, weighed, and homogenized. Meanwhile, the media that supported fungal growth were collected and weighed. *B. bassiana* and the medium metabolites were extracted with 200-500 µL of 80% MeOH. The extracted samples were subjected to non-targeted metabolomics analyses using a UHPLC-qTOF-MS system and targeted analyses using a LC-MS/MS system.

To investigate the metabolic fate of resveratrol in *B. bassiana*, the fungus was cultured on PDA medium supplemented with 100 µM resveratrol and 100 µM $^{13}C_6$-labelled resveratrol (*n* = 5). Cultures were incubated at 25 °C for 7 days. Subsequently, fungal mycelia were harvested and freeze-dried. Metabolites were extracted using 80% methanol (v/v). Extracts were subjected to non-targeted analysis by UHPLC-qTOF-MS to detect and profile resveratrol-derived metabolites.

Non-targeted metabolomics analyses by UHPLC-qTOF-MS

The extracts were analyzed via UHPLC (Thermo Dionex Ultimate 3000, MA, USA) coupled to an qTOF-MS system (Bruker timsTOF, Bremen, Germany). The UHPLC was equipped with a C18 reversed phase column (Zorbax Eclipse XDB-C18, 1.8 µm, 2.1 mm × 100 mm; Agilent Technologies, Böblingen, Germany) maintained at 25 °C and operated at 0.3 mL/min with a gradient flow of 0.1% aqueous formic acid (solvent A) and acetonitrile (solvent B) with the following profile: 5% B from 0–0.5 min, 5–60% B from 0.5 to 11 min, 60–100% B from 11–11.1 min and kept at 100% B until 12 min, then re-equilibrated at 5% B from 12.1–15 min. HRMS analyses were performed in negative ionization mode and automatic MS2 scans ("autoMS") enabled. The source end plate offset was kept at 500 V and the capillary voltage at 4500 V, with the nebulizer gas at 2.8 bar, dry gas at 8 L/min and the drying temperature at 280°C. Ion transfer was performed with a funnel 1 RF of 150 Vpp, funnel 2 RF of 200 Vpp, multipole RF of 50 Vpp, and a deflection delta of 70 V, with the quadrupole ion energy maintained at 4 eV (low mass 90 m/z). The mass scan range was 50–1500 m/z at an acquisition rate of 12 Hz. Collision energies were stepped in a 50:50 timing between a collision energy of 20 eV and 50 eV, respectively. At the beginning of each chromatographic analysis, 10 µL of a sodium formate-isopropanol solution (10 mM solution of NaOH in 50/50 (v/v) isopropanol-water containing 0.2% formic acid) was injected into the dead volume of the sample injection for recalibration of the mass spectrometer using the expected cluster ion *m/z* values. Data were analyzed using the MetaboScape 5.3 software (Bruker, Bremen, Germany) and MetaboAnalyst 5.0 and 6.0 (https://dev.metaboanalyst.ca/). Automated peak picking and alignment were done within a retention time between 0.4 and 11 minutes, signal intensity ≥ 1000, maximum deviation of 2 ppm, and minimum occurrence in at least 4 samples. Feature groups, potentially representing single metabolites, were reduced to one bucket by the MetaboScape software to represent the respective metabolite in later analysis. Peaks deemed not present in specific samples were assigned an intensity value via k-nearest neighbors based on similar features for significant calculations. The data were normalized by sample weight and by $\log_{10}$ transformation. Additionally, features were analyzed using SIRIUS (version 5.8.6) to predict molecular formulas, chemical structures, and chemical taxonomy. The Sirius import module (.mgf) file from MetaboScape software was utilized for this purpose. SIRIUS was applied to the .mgf file to generate predicted molecular formulas for each feature, which were then reranked using ZODIAC (2). Predicted molecular fingerprints were generated using fragmentation trees via CSI:FingerID (3). The chemical taxonomy of predicted metabolite structures was obtained using CANOPUS (superclass, class, and subclass) (4-6). The results of the analyses are listed in the Dataset S1.

Targeted chemical analyses using LC-MS/MS

Targeted analyses of phenolic compounds were performed on an Agilent HP1260 series HPLC instrument (Agilent Technologies, Böblingen, Germany) coupled to an API5000 tandem mass spectrometer (Applied Biosystems, Darmstadt, Germany) or a Triple Quad 6500+ system (SCIEX, MA, USA). Phenolic compounds were separated on an Agilent Zorbax Eclipse XDB-C18 column (50 × 4.6 mm× 1.8 µm, Agilent Technologies, Wilmington, DE, USA) with mobile phase A (0.05% formic acid in milliQ water) and mobile phase B (acetonitrile). The elution profile was: 0–7 min, 10-52% B; 7– 7.01 min, 52-100% B; 7.01– 8 min,

100% B; 8– 8.01 min, 100-10% B; 8.01– 10 min, 10% B at a flow rate of 1.1 mL/min. Detection of each compound was achieved by multiple reaction monitoring (MRM) of specific parent to product ion conversions for each compound. Parameters are described in *SI Appendix*, Table S5. Analyst 1.5 software (Applied Biosystems) and MultiQuant 3.0.3 software (SCIEX) were used for data acquisition and processing. Quantification of individual compounds was achieved by external calibration curves, with the origins of the external standards listed in *SI Appendix*, Table S6.

Identification of phenolic compounds by purification and NMR analyses

Taxifolin-3'-*O*-glucoside was isolated from spruce bark which was collected and freeze-dried as mentioned above. Methylglucosylated phenolics and isorhapontigenin dimers produced by *B. bassiana* were isolated from fungal cultures as follows: over fifty PDA plates containing 1 mM resveratrol, isorhapontigenin, or taxifolin were inoculated with the *B. bassiana* strain 01 and incubated at 25°C for 20 days. Subsequently, the fungi, along with medium, were collected and freeze-dried. The bark and fungal samples used for compound isolation were extracted with pure methanol. The methanol extracts were concentrated using a rotary evaporator R-114 (Büchi, Essen, Germany) and mixed with 5 g of C18 material LiChroprep RP-18 (40-63 µm) (Merck, Darmstadt, Germany). The mixture together with C18 material, was loaded into a prepared C18 column (40 g LiChroprep RP-18) that had been preconditioned with methanol, followed by MilliQ water. Subsequently, the C18 column was washed with a stepwise gradient of aqueous methanol solutions (ranging from 10%, 20%, 30%, 40%, 50% to 60 % MeOH). The elution of targeted compounds was determined using an HPLC (Agilent HP1100 series, Agilent Technologies) ion trap mass spectrometer (ESQUIRE-6000 system, Bruker) equipped with a Nucleodur Sphinx RP column (250 × 4.6 mm × 5 µm, Macherey-Nagel, Düren, Germany). Aqueous formic acid (0.2%, solvent A) and acetonitrile were employed as mobile phases A and B, respectively. The elution profile was as follows: 0-20 min, 10-60% B; 20-20.10 min, 60-100% B; 20.10-22 min, 100% B; 22-22.1 min, 100-10% B; and 22.1-26 min, 10% B at a flow rate of 1.0 mL/min. MS analyses were performed separately with positive and negative ionization, with automatic MS2 scans ("autoMS") enabled(7).

Taxifolin-3'-*O*-glucoside (**3a**) was collected from the 30% MeOH fraction; methylglucosylated phenolic products ((methylglucosylated resveratrol (**1c-*E***, **1c-*Z***, **1d-*E*** or **1d-*Z***), methylglucosylated isorhapontigenin (**2c-*E*** or **2c-*Z***), or methylglucosylated taxifolin (**3c** or **3c-epi**), methylglucosylated quercetin (**3d**)) were collected from the 40% MeOH fraction; and isorhapontigenin dimers (**2d-*E***, **1d-*Z*** or **2e-*Z***) were collected from the 60% MeOH fraction eluted from the SPE C18 cartridge. These solutions were concentrated using a rotary evaporator before further purification by repeated injection into an HPLC-UV system (Agilent Technologies 1100 Series HPLC) equipped with a diode-array detector, using a Nucleodur Sphinx RP column (250 × 4.6 mm × 5 µm, Macherey-Nagel). Phenolic products were detected at 320 nm and collected. Aqueous formic acid (0.1%, solvent A) and acetonitrile were used as mobile phases A and B, respectively. The elution profile was as follows: 0-12 min, 25-50% B; 12-12.10 min, 50-100% B; 12.10-14 min, 100% B; 14-14.1 min, 100-25% B and 14.1-18 min, 25% B at a flow rate of 1.0 mL/min. The system

was equipped with a fraction collector (Advantec SF-2120), and the collected compounds were concentrated using a rotary evaporator.

NMR measurements were carried out on a 700 MHz Bruker Avance III HD spectrometer (Bruker Biospin GmbH, Rheinstetten, Germany) and a 500 MHz Bruker Avance III HD spectrometer (Bruker Biospin GmbH, Rheinstetten, Germany) using standard pulse sequences as implemented in Bruker Topspin ver. 3.6.1. The spectrometer was equipped with a TCI cryoprobe. Chemical shifts were referenced to the residual solvent signals of MeOH-d_3 (δH 3.31/δC 49.0), MeCN-d_3 (δH 1.94/δC 1.32). All spectra were recorded at 298 K. Compounds with a stilbene skeleton can easily be isomerized by light (including a UV detector in HPLC), which makes handling them as pure stereoisomers difficult. Therefore, they were measured and structurally determined as mixtures. Additionally, **3c-epi** underwent isomerization during the measurement so that ^{13}C measurement, which requires long measurement time, was not performed. Compounds for which ^{13}C data could not be obtained due to stability or quantity issues were structurally determined using HSQC and HMBC.

Total RNA isolation and cDNA syntheses

Total RNA was isolated from *B. bassiana* using the RNeasy plant mini kit (Qiagen, Hilden, Germany) and genomic DNA contamination was eliminated by RNase-free DNase (Qiagen). The quantity and quality of RNA in each sample were determined using a NanoDrop 2000c instrument (Thermo Fisher Scientific). cDNA was synthesized using SuperScript III Reverse transcriptase kits (Invitrogen, Waltham, MA, USA).

Recombinant BbGT86 and BbMT85 expression in yeast

To determine the specific activities of BbGT86 and BbMT85 with phenolic compounds, these proteins were heterologously expressed in yeast cells. The BbGT86 and BbMT85 encoding genes were cloned from the isolated *B. bassiana* strain. The complete open reading frames of *gt86* and *mt85* mRNA sequences were cloned from the synthesized cDNA pool obtained from primer pairs FLF and FLR (*SI Appendix,* Table S3), and their sequences have been submitted to NCBI (accession codes are listed in *SI Appendix*, Table S4). Restriction enzyme cutting sites were added to the ends of the cloned full-length *gt86* or *mt85* using the primer pairs VF and VR (*SI Appendix*, Table S3), and the fragments were further digested by restriction enzymes (Thermo Fisher Scientific). The pESC-Leu vector, which contains two protein expression sites, was used to express the target proteins. After the pESC-Leu vector was restriction digested accordingly, the restriction-digested *gt86 and mt85* fragments were inserted into the restriction-cut pESC-Leu cloning site by T4 DNA ligase (Invitrogen), either separately or simultaneously, to form the pESC (BbGT86), pESC (BbMT85), and pESC (BbGT86 + BbMT85) vectors. pESC (EV) without protein inserts was used as negative control. The vectors were then transformed into *Saccharomyces cerevisiae strain* INV*Sc*1 using the S.c. EasyComp Transformation Kit (Invitrogen) following the manufacturer's instructions. Subsequently, 30 mL Sc-Leu minimal medium (6.7 g/L yeast nitrogen base without amino acids, with ammonium sulfate; 1.6 g/L synthetic drop-out medium without leucine; 75mg adenine; 20 g/L D-glucose) was inoculated with single yeast colonies and grown overnight at 28°C and 180 rpm. For main

cultures, 100mL YPDA (Glc) full medium (10 g/L yeast extract, 20 g/L bactopeptone, 74 mg/L adenine hemisulfate, 20 g/L D-glucose) was inoculated with one OD_{600} unit of the overnight cultures and incubated under the same conditions for 30–35 hours. After centrifugation (4,500 rpm,16°C, 5 min), protein expression was induced by resuspension of the cells in 100mL YPGA (Gal) medium (same as YPDA but with 20 g/L galactose instead of D-glucose) and grown for another 15–18 hours at 25°C and 160 rpm. Subsequently, 10 mL YPGA culture was supplemented with 20 g/L D-glucose and 100 µM phenolic compounds for 24 hours at 25°C and 160 rpm**.** 200 µL of the cells and medium were added to 800 µL of pure methanol, which was subsequently used for untargeted analysis by UHPLC-qTOF-MS to detect enzyme activity.

Crystal structures of BbGT86 and BbMT85 were acquired from the AlphaFold server (https://alphafoldserver.com/) based on the protein sequences. The docking result and 3D geometries were observed using the PyMOL Molecular Graphics system (V 3.1.4.1, Schrödinger, LLC).

B. bassiana gene modification via _Agrobacterium tumefaciens_-mediated transformation (ATMT)

To construct homologous recombination cassettes targeting the replacement of *mt85*, *gt86* or both *mt85* and *gt86*, approximately 1-2 kb 5' and 3' flanking regions of the targeted genes were amplified from *B. bassiana* strain 01 genomic DNA. The pBarGPE1 (Bioactiva Diagnostica, Höhe, Germany) plasmid, containing the TrpC promoter and the Basta resistance gene (*bar*), was linearized using XbaI or SpeI restriction enzymes (Thermo Fisher Scientific). The 5' flanking regions of *gt86* or *gt86mt85* were inserted into the linearized pBarGPE1 predigested with XbaI via Gibson ligation using Gibson assembly master mix (New England Biolabs, Ipswich, USA). Subsequently, the plasmid was further linearized using SpeI, followed by the insertion of 3' flanking regions of *gt86* or *gt86mt85* via Gibson ligation. The constructed pBar*gt86mt85* and pBar*gt86* plasmids contained cassettes of the 5' flanking region:TrpC promotor: *bar* gene: 3' flanking region (Fig. 6A). The $pCAMBIA^{XmnI}$ vector was generated by eliminating the bacterial hygromycin B resistance gene from the binary pCAMBIA1300 plasmid (Abcam, Cambridge, England) using XmnI digestion (8). The homologous recombination cassettes were cloned from the p*Bargt86* and p*Bargt86mt85* plasmids and inserted into the SmaI-linearized binary $pCAMBIA^{XmnI}$ vector via Gibson ligation, forming the pB-*Bargt86* and pB-*Bargt86mt85* vectors for ATMT.

To construct the *B. bassiana* strains for whole-mount immunostaining in infected bark beetles, the pB-*BareGFP* vector was constructed for eGFP overexpression in *B. bassiana*. The Basta resistance gene and *eGFP* genes were cloned from plasmid of pBarGPE1-EGFP (Bioactiva Diagnostica, Höhe, Germany). The cloned fragment was inserted into the SmaI-linearized binary $pCAMBIA^{XmnI}$ vector via Gibson ligation, forming the pB-*BareGFP.* All primer pairs are listed in *SI Appendix*, Table S3.

The *B. bassiana mt85* and *gt86* homologous recombination vectors (pB-*Bargt86* and pB-*Bargt86mt85*), as well as *B. bassiana Bar-eGFP* overexpression vector (pB-*BareGFP*) were electroporated into AGL-1 *Agrobacterium* electrocompetent cells (GoldBio, St Louis MO, USA) using a Gene Pulser (Bio-Rad, Hercules, CA) under standard conditions. ATMT was performed following a modified method from Moon et al (9). The pB-*Bargt86*, pB-*Bargt86mt85*, and pB-*BareGFP* transfected GAL-1 cells were grown in

50 mL of LB medium with kanamycin (50 µg/mL) and gentamicin (30 µg/mL) for 2 days at 29°C until reaching an optical density (OD_{600}) of 0.6-0.8. The cells were washed with induction medium (containing 10 mM K_2HPO_4,10 mM KH_2PO_4, 2.5 mM NaCl, 2 mM $MgSO_4$, 0.7 mM $CaCl_2$,9 µM $FeSO_4$,4 mM $(NH_4)_2SO_4$, 10 mM glucose, 40 mM 2-[N-morpholino] ethanesulfonic acid (MES, pH 5.3), 0.5% glycerol (w/v), and 200 µM acetosyringone) and diluted with induction medium to an OD_{600} of 0.15. They were then grown overnight at 29°C until reaching an OD_{600} of 0.5. *B. bassiana* strain 01 conidia were collected from cultures grown on PDA for 2 weeks at 25°C as described above. The pB-*Bargt86*, pB-*Bargt86mt85*, and pB-*BareGFP* transfected GAL-1 cells were mixed 1:1 with *B. bassiana* conidia (10^5 spores/ mL), and 200 µL of this mixture was plated on nitrocellulose filters (0.45 µm pore size, 47 mm diameter, Carl Roth) on co-cultivation medium (same as induction medium but containing 5 mM glucose instead of 10 mM). The plates were incubated for 2 days in the dark at 28°C. After incubation, the membranes were transferred to Czapek-Dox Agar (CDA) plates containing glufosinate ammonium (200 µg/mL) as the selection agent for fungal transformants and mefoxin (300 µg/mL) to inhibit the growth of *A. tumefaciens*. Plates were incubated for 5 to 7 days in the dark at 25°C until transformants had grown.

Each primary transformant was transferred to a single CDA plate containing glufosinate ammonium (200 µg/mL) and mefoxin (300 µg/mL) and incubated at 25°C. Meanwhile each transformant was shaken in a 5 mL Czapek-Dox Broth (CDB) with mefoxin (300 µg/mL) at 25°C and 200 rpm for 5 to 7 days. Fungal mycelium was collected for genomic DNA extraction using the MyTaq Extract-PCR Kit (Biocat, Heidelberg, Germany). The miniprep genomic DNA was screened by PCR to detect homologous recombination events, where the gene disruption construct containing the *bar* cassette was inserted into the wild-type gene, and to detect the insertation of *eGFP* fragment. DNA was amplified using 2× MyTaq HS Red Mix (Biocat) with the primer pairs listed in *SI Appendix*, Table S3 which used to detect the construct for *B. bassiana* mutants. The selected Δ*Bbgt86*, Δ*Bbgt86mt85*, and *B. bassiana eGFP* mutants were maintained on selective plates for 5 generations. All experiments involving genetically modified *B. bassiana* strains were conducted in a biosafety level 2 (S2) laboratory at the Max Planck Institute for Chemical Ecology.

To confirm successful gene knockout in the strains Δ*Bbgt86* and Δ*Bbgt86mt85*, these mutant fungi together with *B. bassiana* WT (as positive control) were incubated with 400 µM resveratrol, isorhapontigenin, and taxifolin, respectively for 9 days at 25°C. The harvested fungal biomass was freeze-dried, weighed, homogenized, and extracted in 80% MeOH. The metabolites of phenolic compounds were detected via non-targeted metabolomics analyses using a UHPLC-qTOF-MS system.

Whole-mount immunostaining

The procedure for immunostaining of bark beetles was adapted from an established protocol with slight modifications (10). Newly emerged bark beetles were fed with artificial diet containing either *B. bassiana* WT fungi or genetically engineered *B. bassiana eGFP* fungi for 7 days. Then, the infected bark beetles were temporarily stored in the freezer for one day before the following whole-mount immunostaining treatment. The beetles were dissected longitudinally, rinsed in PBS buffer for one hour, and fixed in ZnFA

at room temperature for 20 h. The ZnFA fixative contained 0.25% (18.4 mM) $ZnCl_2$, 0.788% (135 mM) NaCl, 1.2% (35 mM) sucrose, 1% formaldehyde and 0.1% DMSO. Fixed beetles were rinsed in saline buffer for 3x 20 min followed by a standard dehydration-rehydration process with an ethanol series of 30%, 50%, 70%, 90%, 96% and 100%, with 10 min each. The beetles were then treated with blocking buffer (5% NGS, 1% DMSO, 0.005% NaN_3 in 0.1 M PBS) for 1h at room temperature, which was then replaced with primary antibodies (mouse anti-GFP) diluted in the blocking buffer at 1:1000, lasting for 4 days at room temperature. The beetles were rinsed in PBS-DMSO for 3x 45 min and treated with secondary antibodies (goat anti-mouse, Alexa fluor 488, diluted in blocking buffer at 1:1000) for 4 days at room temperature. Finally, the stained beetles were washed in PBSD for 3x 45 min and were permanently mounted with Vectashield (VectorLabs).

Effects of phenolic compounds on *B. bassiana* growth and metabolites

The growth of wild-type *B. bassiana* strain 01 and mutants Δ*Bbgt86* and Δ*Bbgt86mt85* was monitored on PDA plates. A 1 µL drop containing 10^8 conidia per mL was placed at center of each plate. The fungal colony growth was measured at 9-, 11-, 13-, and 15-days post-inoculation at 25°C. Meanwhile, to assess the effect of the *gt86* or *gt86mt85* gene knockouts on *B. bassiana* development and phenolic metabolites, the wild-type strain and mutant strains were grown on PDA plates with varying concentrations (0–800 µM) of piceid, resveratrol, or methylglucosylated resveratrol. A 1 µL drop containing 10^8 conidia per mL was used, and colony growth was measured 9 days post-inoculation at 25°C. Each treatment had 4 replicates. The diameters of the fungal colonies were measured in two perpendicular directions, and the average was used to calculate the colony area.

Additionally, wild-type *B. bassiana*, Δ*Bbgt86* and Δ*Bbgt86mt85* mutants were incubated on PDA plates supplemented with 400 µM resveratrol or a solvent control for 9 days at 25°C (n = 5 for each treatment). The harvested fungal biomass was freeze-dried, weighed, homogenized, and extracted in 80% MeOH. The extracted samples were subjected to non-targeted metabolomics analyses using a UHPLC-qTOF-MS system and targeted analyses using a LC-MS/MS system.

Effects of phenolic metabolism by *B. bassiana* on successful bark beetle infection

To quantify the infection efficiency of wild-type *B. bassiana*, Δ*Bbgt86*, and Δ*Bbgt86mt85* mutants on bark beetle hosts, callow bark beetles were infected with the isolated wild-type *B. bassana* and mutant strains. The infection of callow adults was carried out as described above. There were 3-6 replicates for each treatment and around ten samples taken as one replicate. Both infected and uninfected bark beetles were reared in tubes of semi-artificial diet for 10 days at 25°C. Bark beetle mortality was recorded on the 10th day post-infection, with mortality calculated by comparing the number of surviving adults to the initial number of callow adults introduced.

Additionally, transcript abundance analysis was performed to assess the virulence of *B. bassiana* on bark beetle. Wild-type *B. bassiana*, Δ*Bbgt86*, and Δ*Bbgt86mt85* infected bark beetles and uninfected

bark beetles were immediately frozen in liquid nitrogen on the 10th day post-infection. Genomic DNA was extracted, and qPCR was conducted as previously described. The bark beetle *RPS3-a* (40S ribosomal protein S3-A) gene (11) was used as an internal control to normalize the abundance of *B. bassiana actin* gene transcripts (n = 4 for each treatment). Primer pairs and gene accession numbers are listed in *SI Appendix*, Table S3 and Table S4, respectively.

Furthermore, bark beetles infected with wild-type *B. bassiana*, Δ*Bbgt86*, and Δ*Bbgt86mt85* mutants, as well as uninfected bark beetles were collected on the 10th day post-infection. Meanwhile, the diet without bark beetle rearing, and diet on which bark beetles were reared, as well as wild-type *B. bassiana* infected bark beetles were collected. Metabolites were extracted from the bark beetles and from the semi-artificial diet as described above. The extracted samples were subjected to non-targeted metabolomics analyses using a UHPLC-qTOF-MS system and targeted analyses using an LC-MS/MS system.

Figures

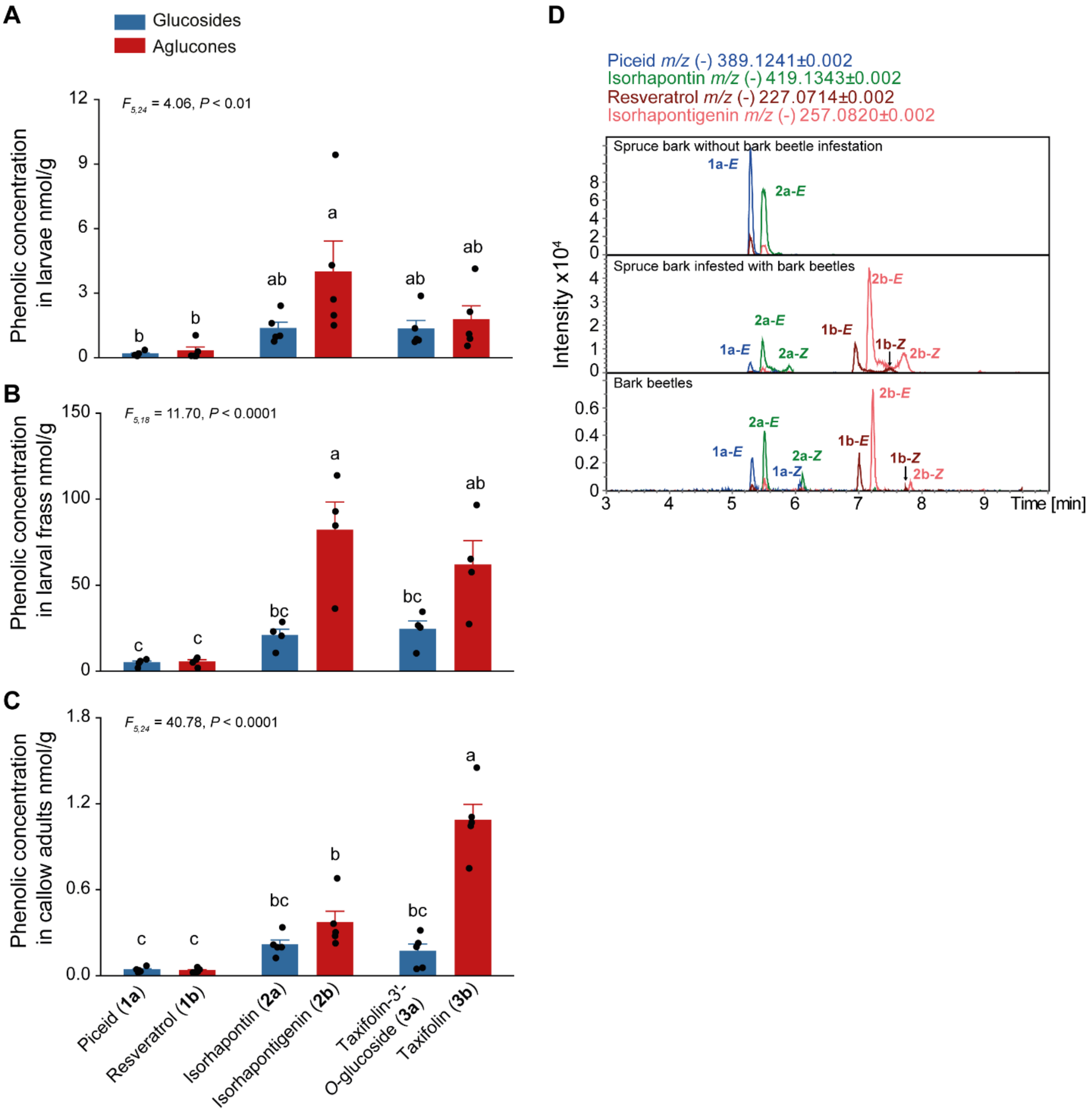

Fig. S1 Metabolism of phenolic glucosides by bark beetles resulting in the formation of phenolic aglucones. (**A-C**) Phenolic content measured in bark beetle larvae (*n* = 5) (**A**), larval frass (*n* = 4) (**B**), and callow adults (*n* = 5) (**C**). The compound **1a** represents the combined quantity of its trans- and cis-isomers (**1a-*E*** and **1a-*Z***), similar to compounds **1b**, **2a**, and **2b**. (**D**) Extracted ion chromatograms in negative ionization mode of phenolic compounds in the intact bark of spruce trees, in the bark tunneled by bark beetles, and in bark beetle adults measured by UHPLC-qTOF-MS. Mass spectra of phenolic metabolites and phenolic standards are available in Edmond. Significant differences between means (±SE) were determined using one-way ANOVA followed by Tukey's HSD tests in **A-C.** Different lowercase letters indicate statistically significant differences ($p < 0.05$).

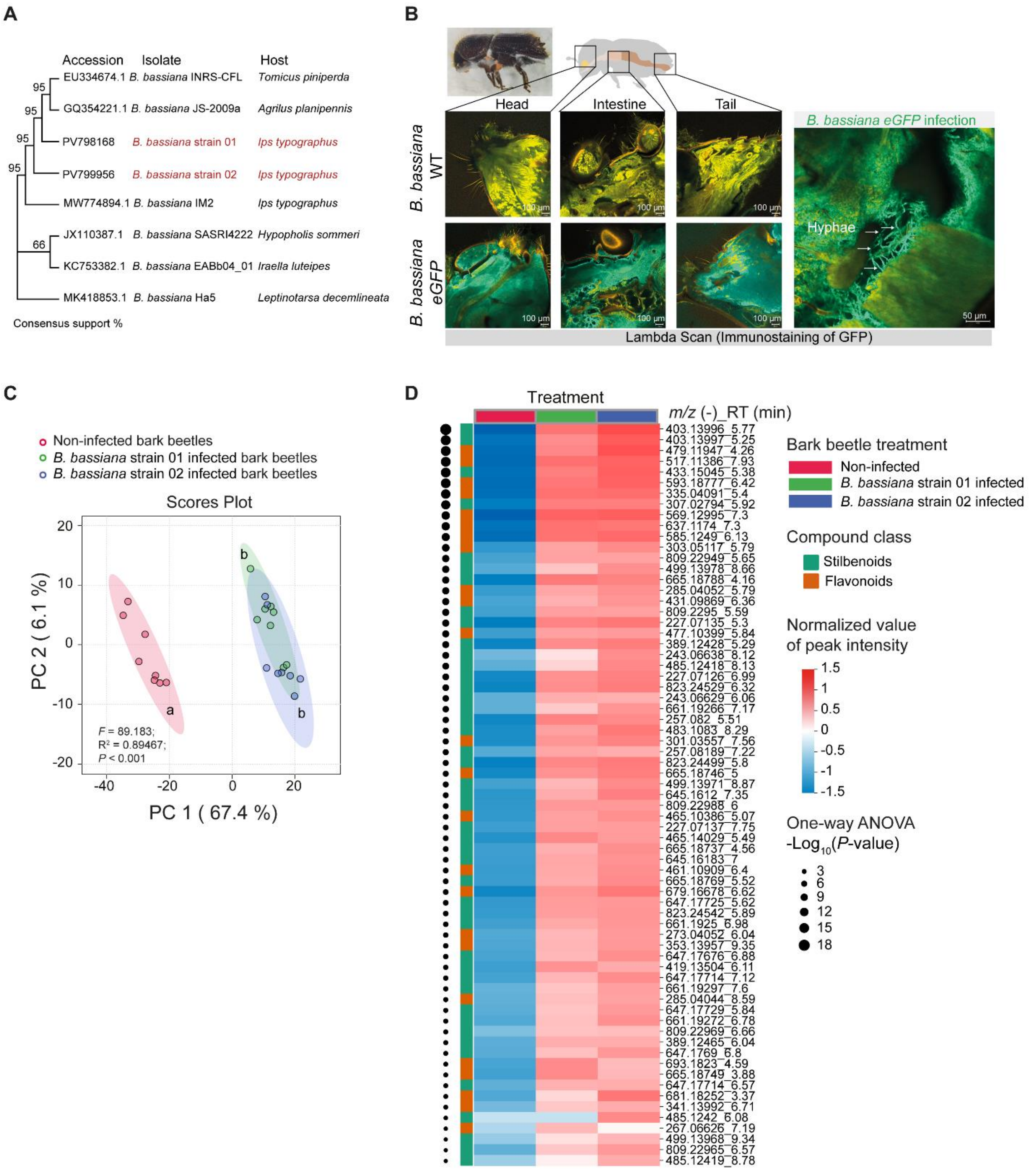

Fig. S2 *B. bassiana* infections change the metabolic profiles of bark beetles. (**A**) Phylogenetic analysis of the *B. bassiana* internal transcribed spacer (ITS) region, showing strain diversity. *B. bassiana* strains 01 and 02 were isolated from infected bark beetles. The ITS gene of *B. bassiana* sequences were aligned, and a UPGMA tree was generated. Branch labels indicate consensus support (%). Accession numbers are listed in the figure. (**B**) Immunostaining of the bark beetles infected with *B. bassiana* wild-type (WT) and *B. bassiana eGFP* knock in mutant strains. (**C**) Principal component analysis (PCA) plot showing the variation in metabolic profiles among uninfected bark beetles and those infected with *B. bassiana* strains 01 or *B. bassiana* 02. The circles indicate 95% confidence regions. (**D**) Heatmap of features of flavonoids and stilbenoids from non-targeted UHPLC-qTOFMS analyses (in negative mode) of *B. bassiana* 01 and 02

infected bark beetles fed on a semi-artificial diet compared to uninfected bark beetles (n = 8 per treatment). Data are based on peak intensity, normalized by sample weight and $\log_{10}$-transformed in panels **C** and **D**. Significant metabolites formed by *B. bassiana*-infected bark beetles compared to uninfected bark beetles are listed in Supplementary Data file. Statistically significant differences between means (± SE) were determined using pair-wise PERMANOVA in **C**, and one-way ANOVA followed by Fisher's LSD tests in **D**. Different lowercase letters denote statistically significant differences ($p < 0.05$).

^{1}H NMR with water presaturation full range in MeOH-d_3

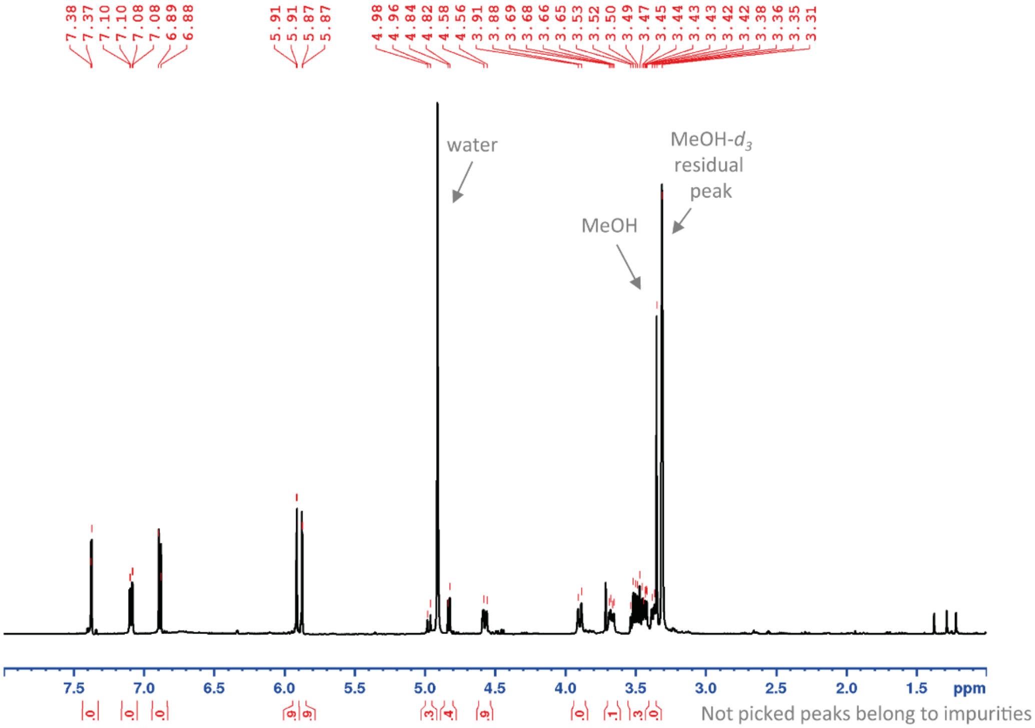

DEPTQ full range in MeOH-d_3

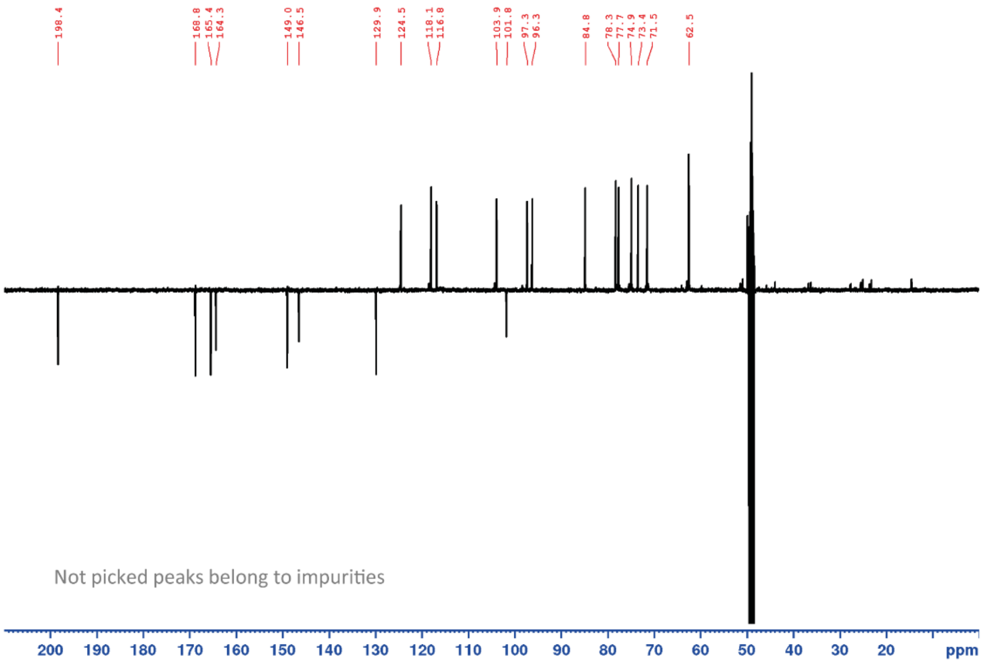

Fig. S3A NMR spectra for taxifolin-3'-*O*-β-glucopyranoside (3a).

^{1}H NMR with water presaturation full range in MeOH-d_3

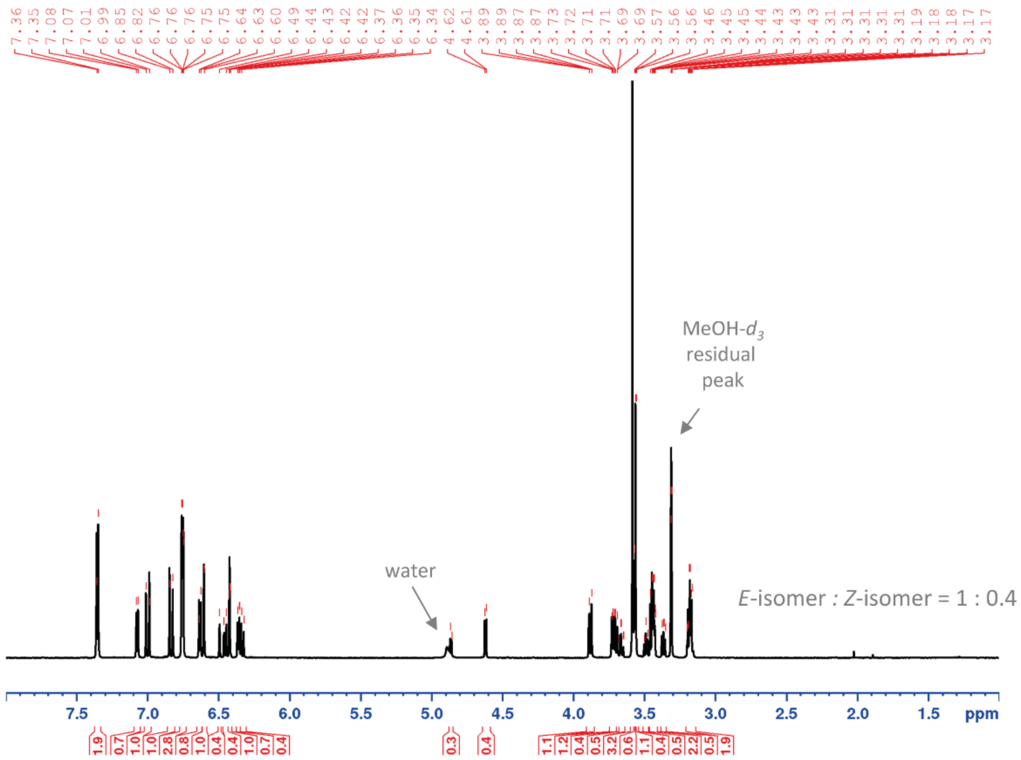

DEPTQ full range in MeOH-d_3

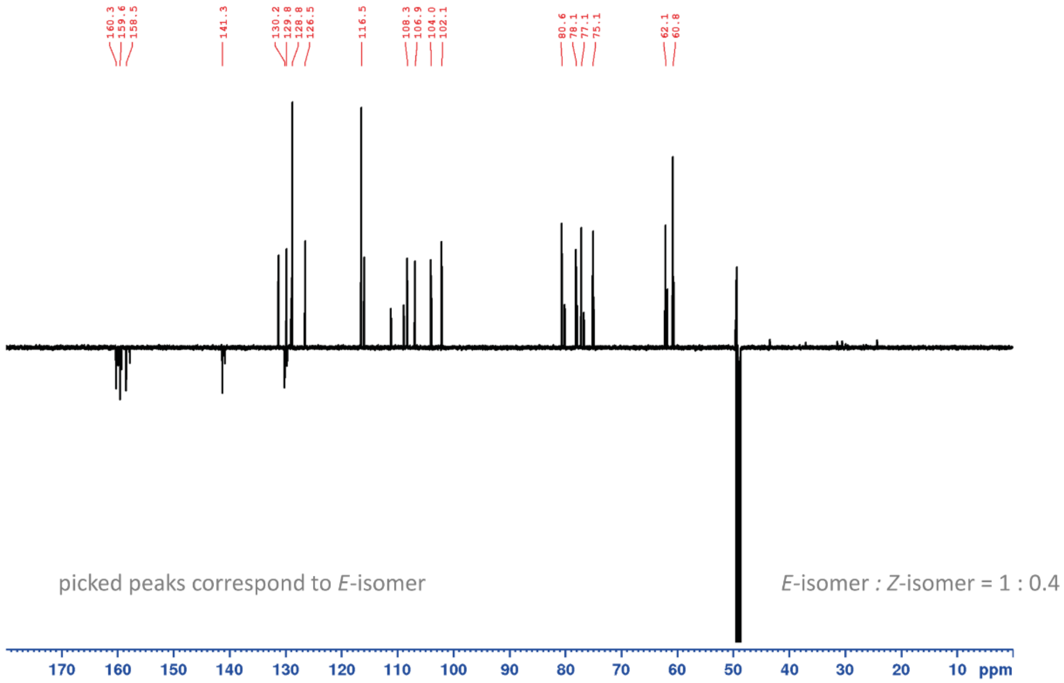

Fig. S3B NMR spectra for (*E*)-resveratrol-3-*O*-β-(4'-*O*-methyl)glucopyranoside (1c-*E*).

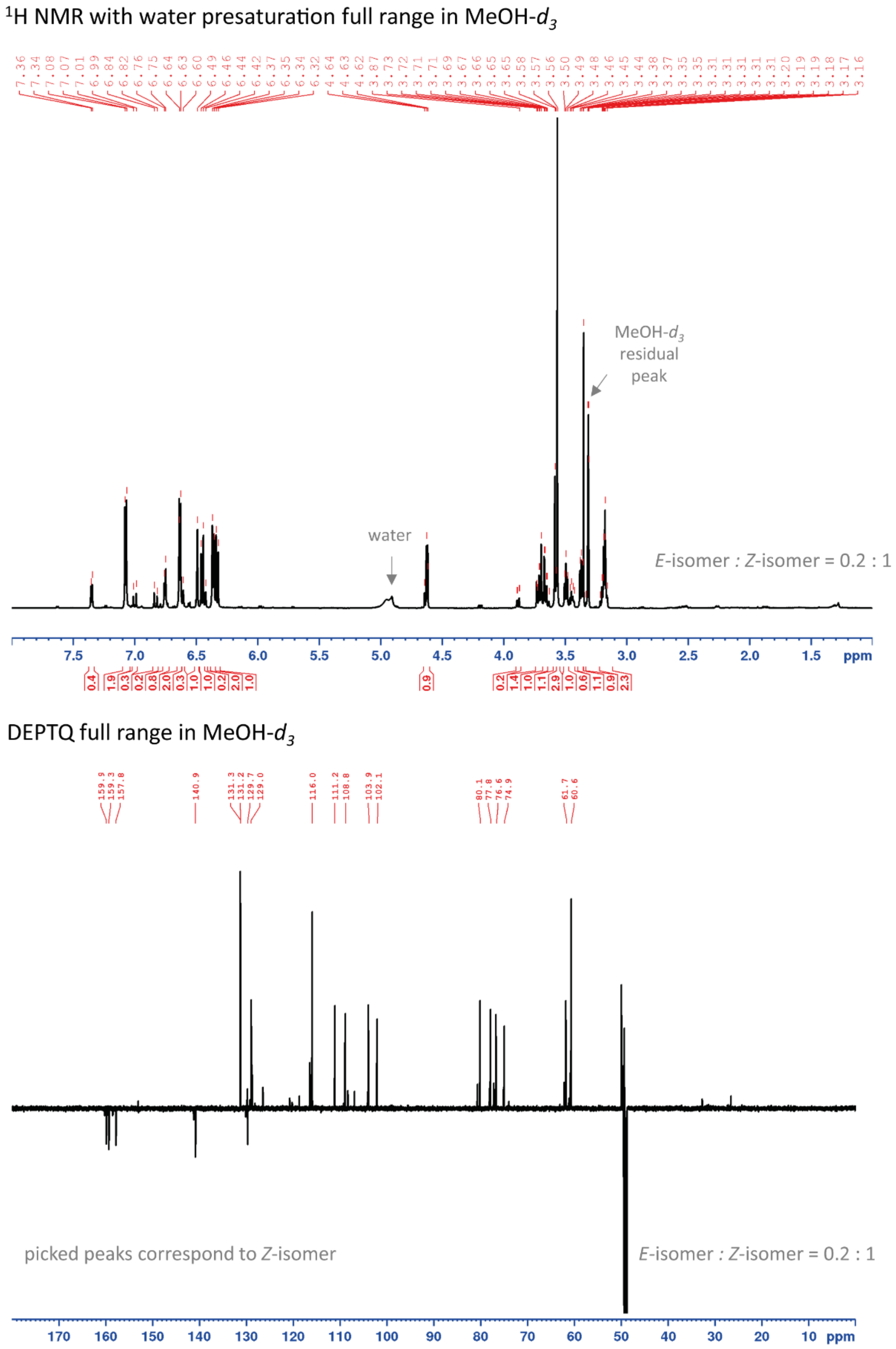

Fig. S3C NMR spectra for (*Z*)-resveratrol-3-*O*-β-(4'-*O*-methyl)glucopyranoside (1c-*Z*).

^{1}H NMR with water presaturation full range in MeOH-d_3

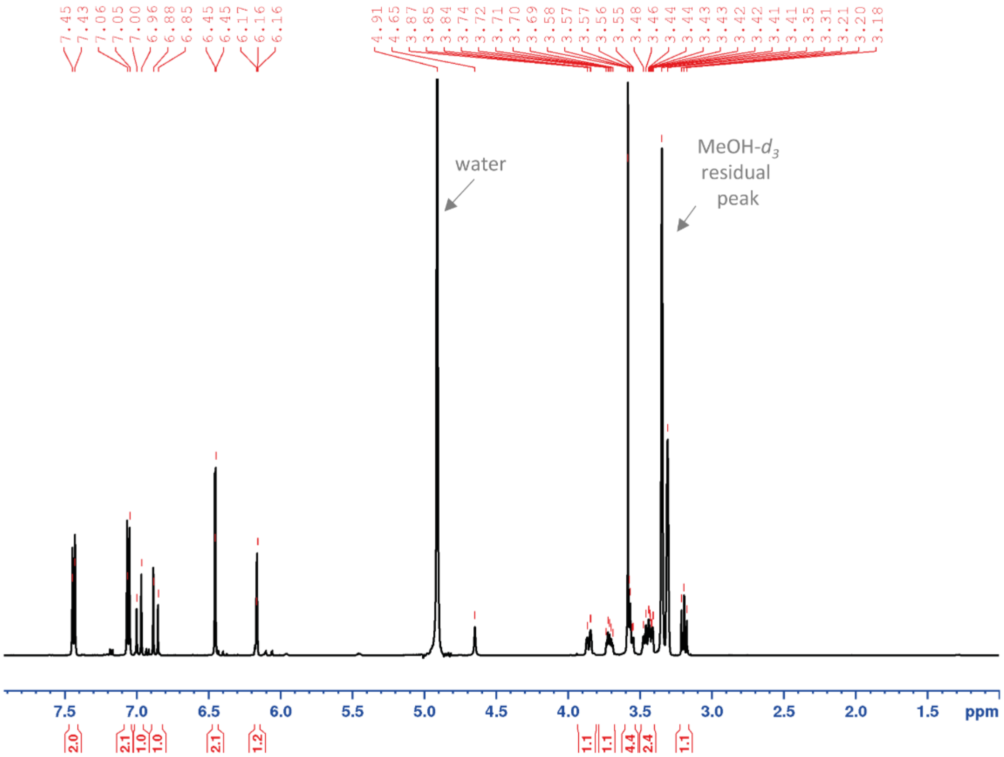

DEPTQ full range in MeOH-d_3

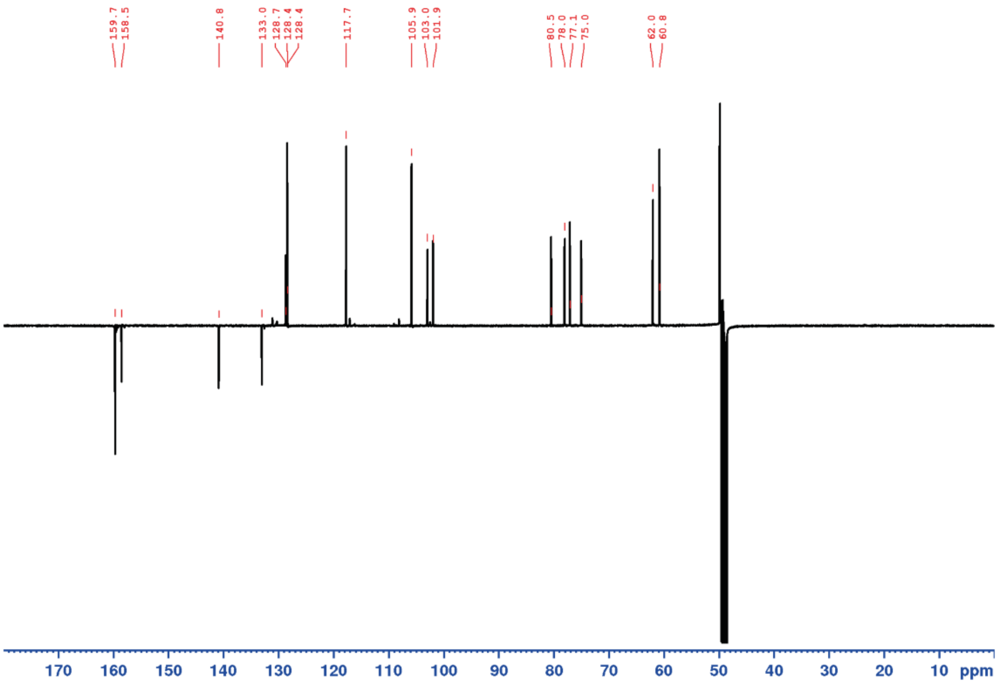

Fig. S3D NMR spectra for (*E*)-resveratrol 12-*O*-β-(4'-*O*-methyl)glucopyranoside (1d-*E*).

^{1}H NMR with water presaturation full range in MeOH-d_3

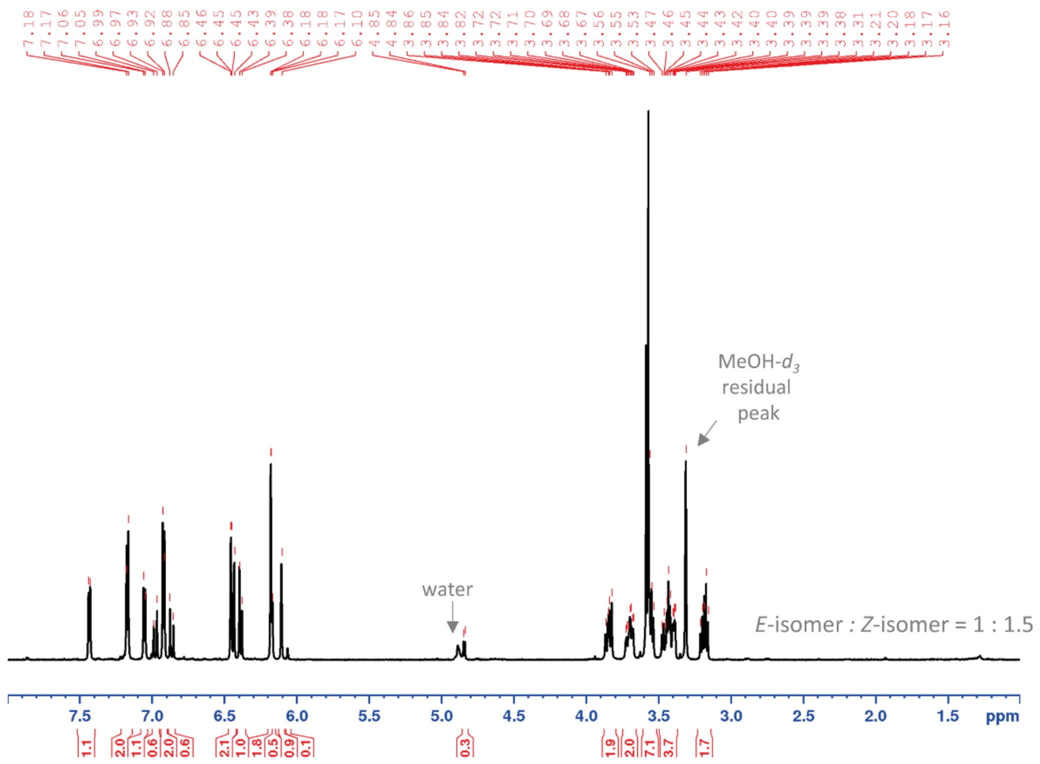

DEPTQ full range in MeOH-d_3

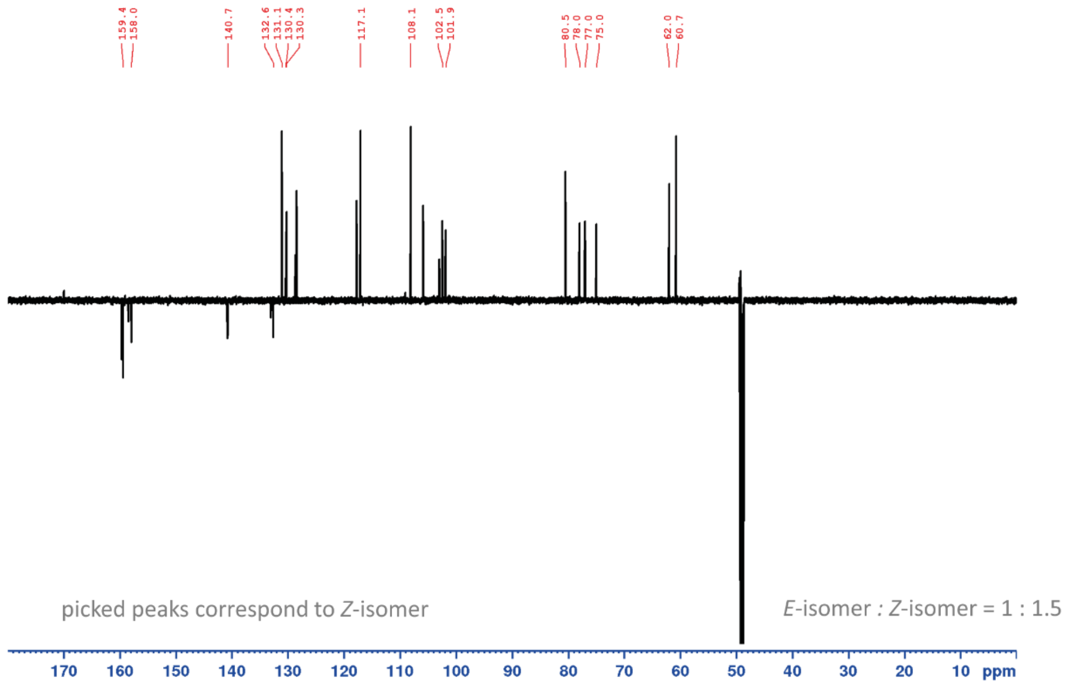

Fig. S3E NMR spectra for (*Z*)-resveratrol 12-*O*-β-(4'-*O*-methyl)glucopyranoside (1d-*Z*).

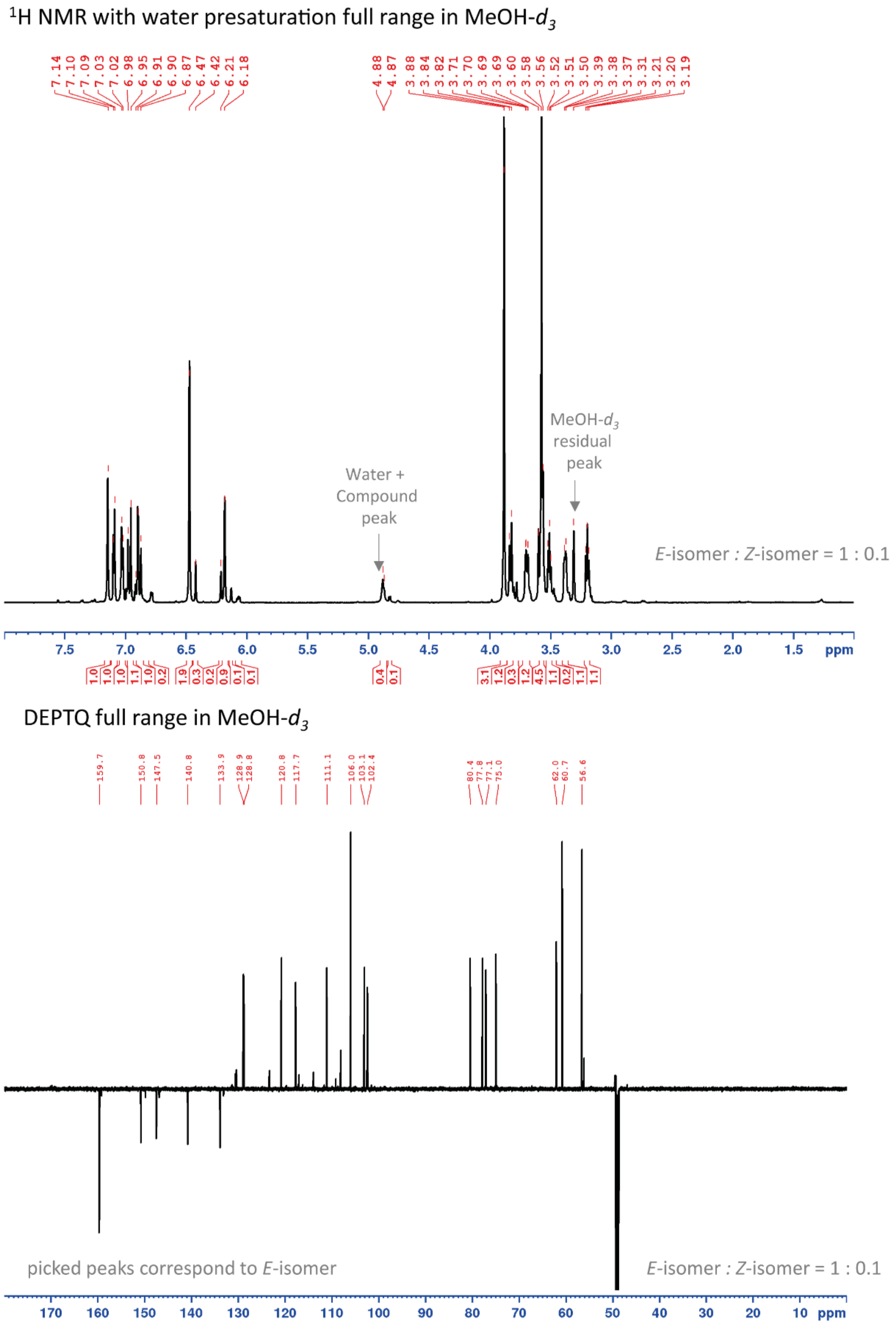

Fig. S3F NMR spectra for (*E*)-isorhapontigenin-12-*O*-β-(4'-*O*-methyl)glucopyranoside (2c-*E*).

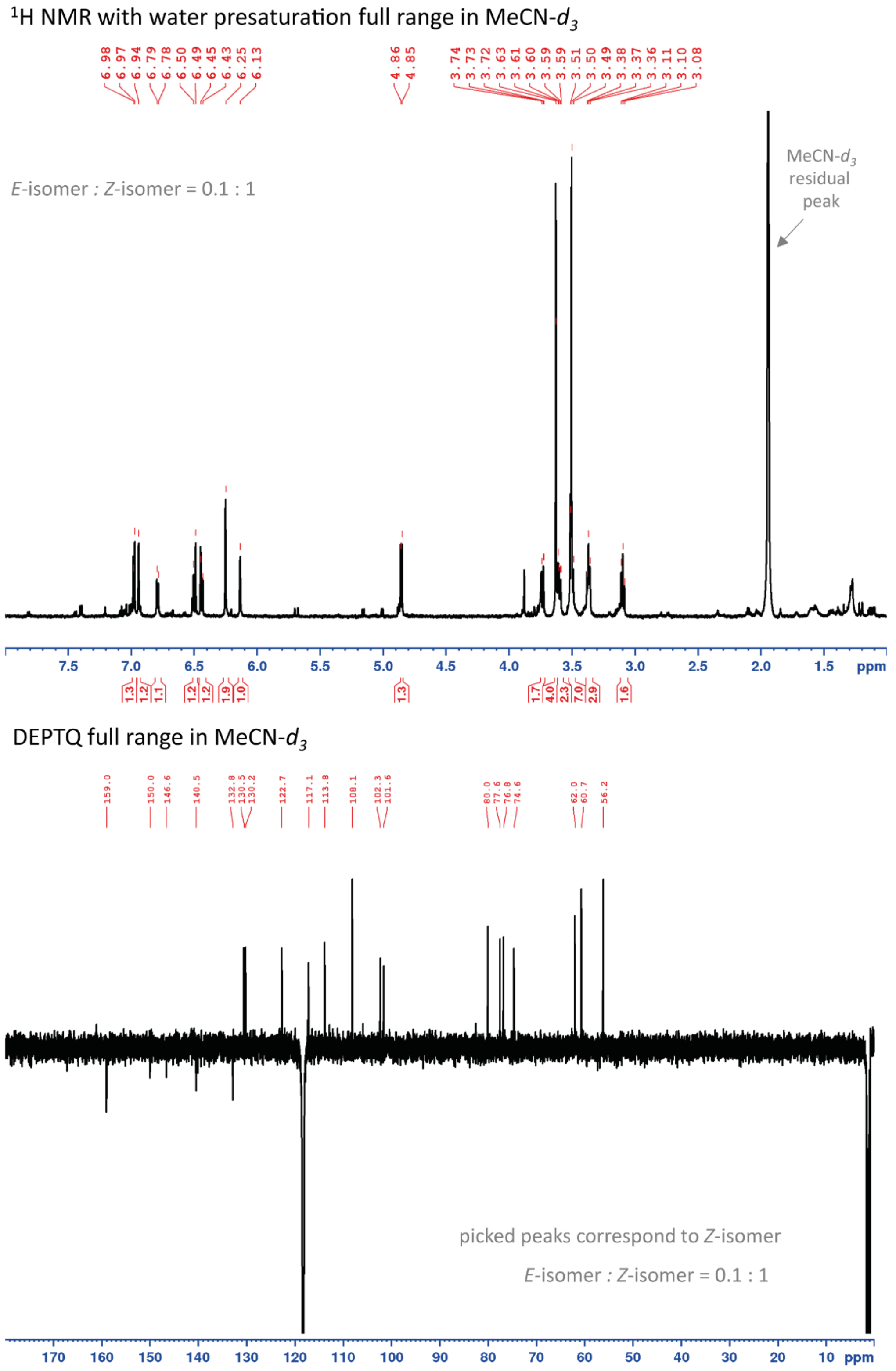

Fig. S3G NMR spectra for (*Z*)-isorhapontigenin-12-*O*-β-(4'-*O*-methyl)glucopyranoside (2c-*Z*).

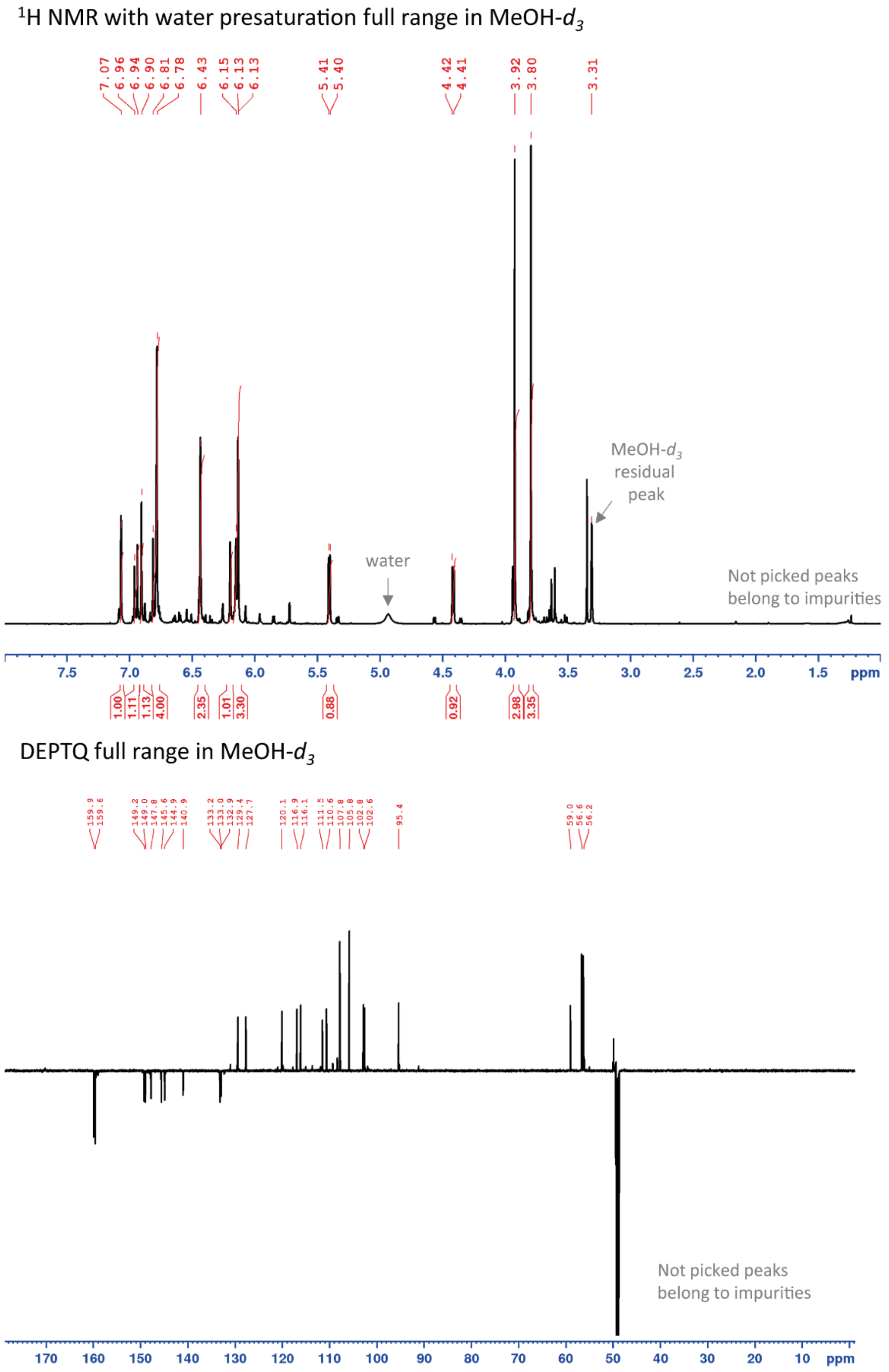

Fig. S3H NMR spectra for shegansu B (2d-*E*).

^{1}H NMR with water presaturation full range in MeOH-d_3

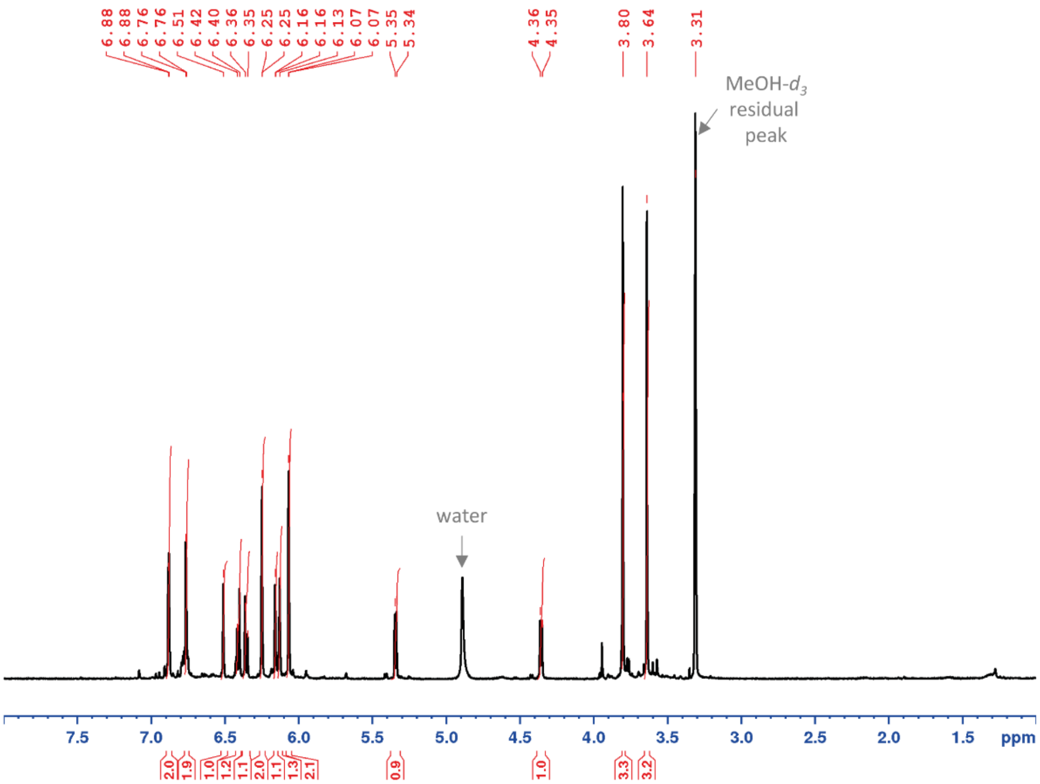

DEPTQ full range in MeOH-d_3

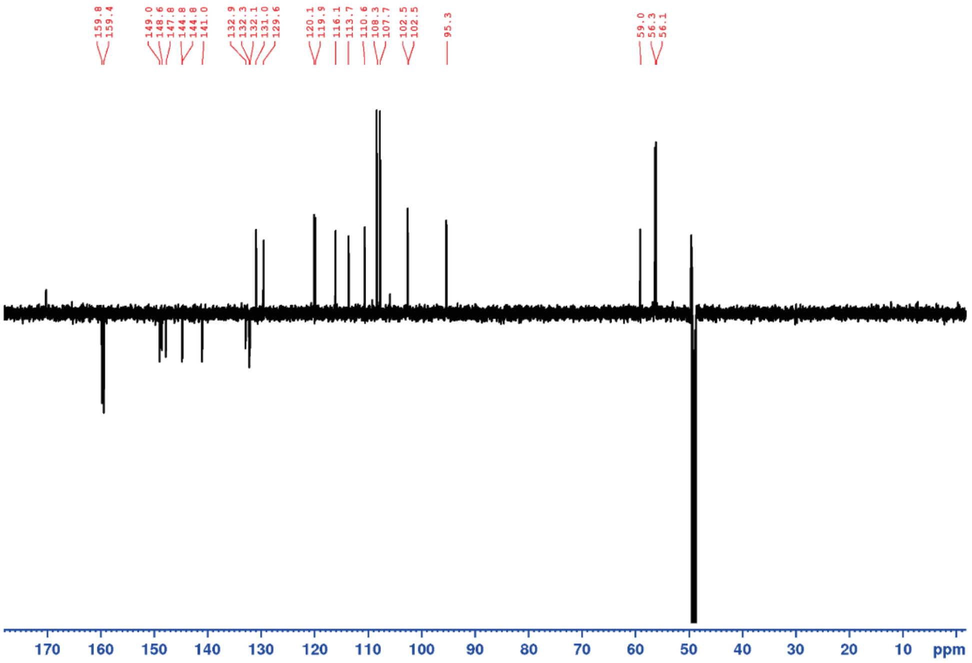

Fig. S3I NMR spectra for (*Z*)-shegansu B (2d-*Z*).

^1H NMR with water presaturation full range in MeOH-d_3

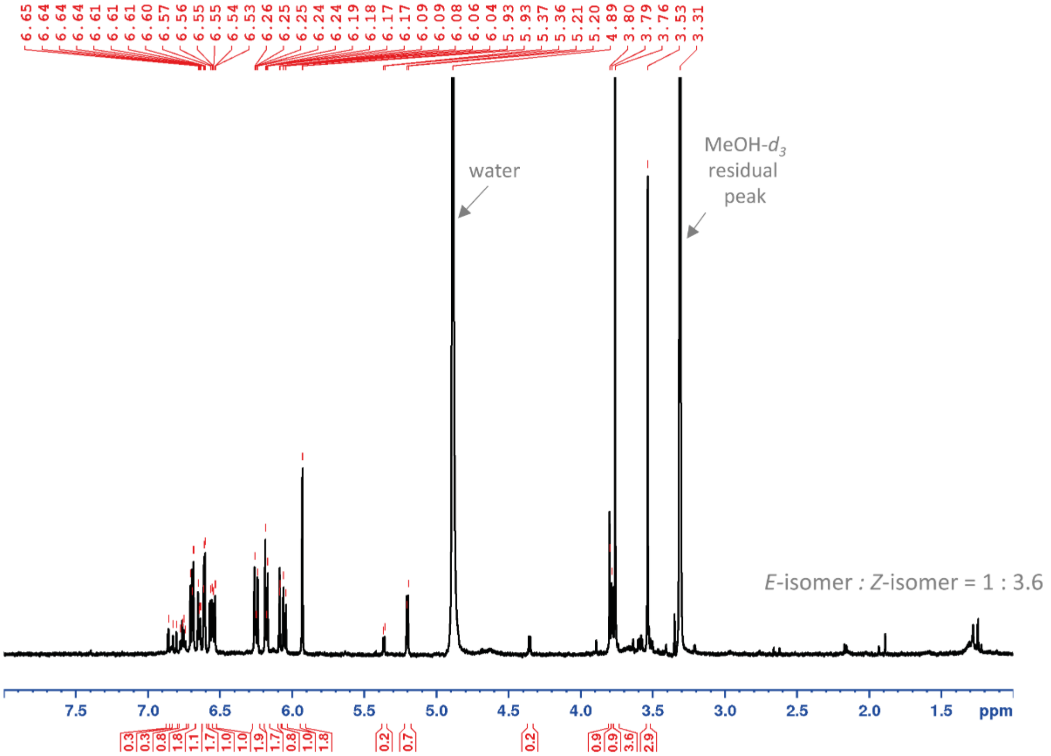

Phase sensitive HSQC (blue: CH, CH_3, green CH_2)/ HMBC (red) full range in MeOH-d_3

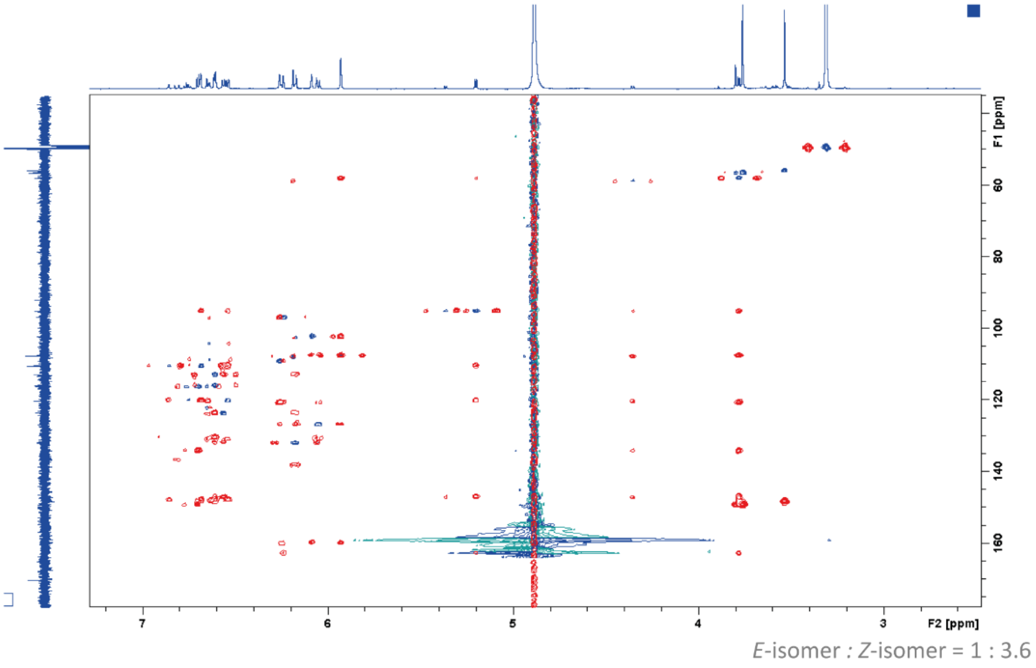

Fig. S3J NMR spectra for (*Z*)-bisisorhapontigenin A (2e-*Z*).

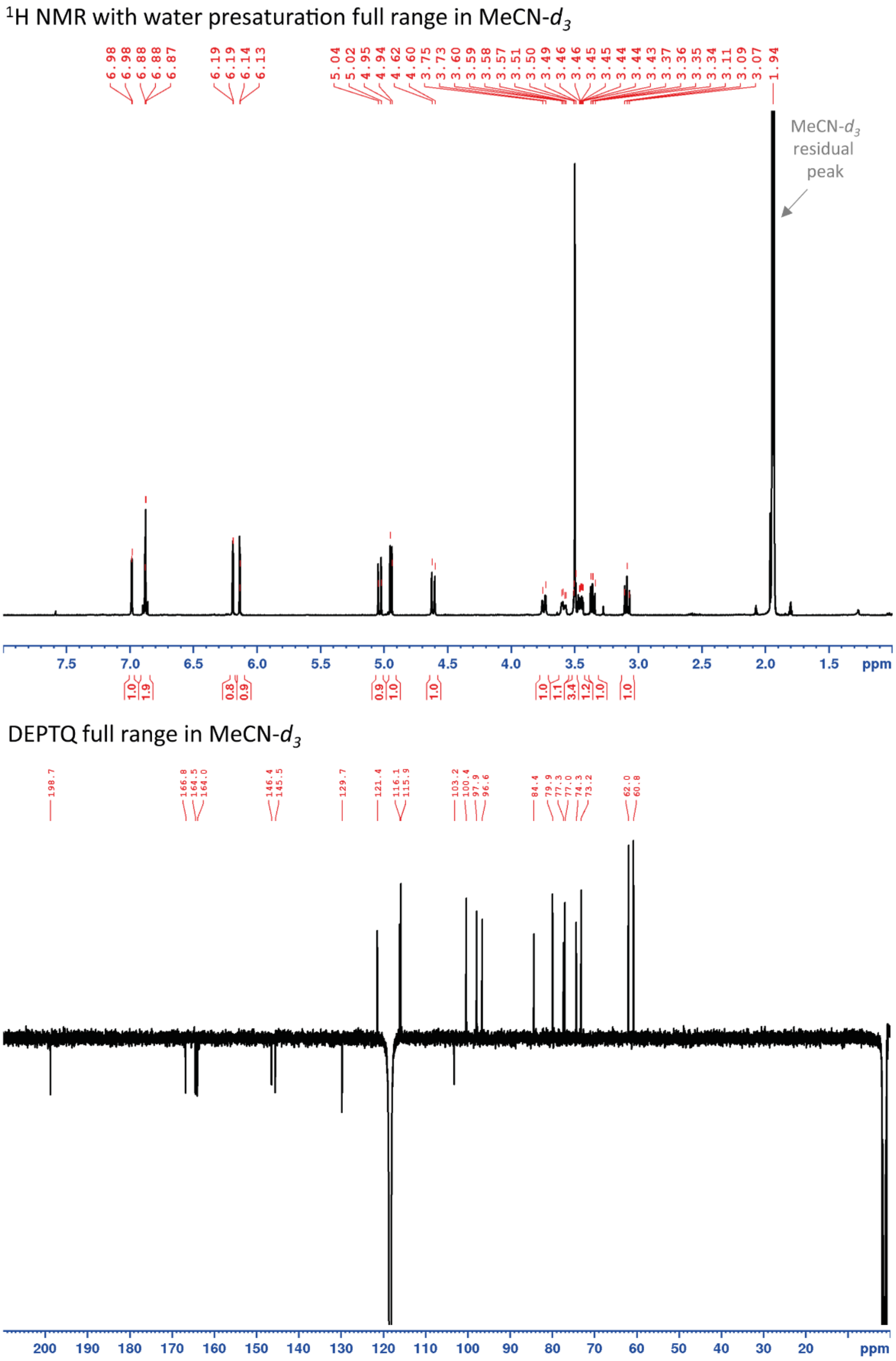

Fig. S3K NMR spectra for taxifolin-7-*O*-β-(4'-*O*-methyl)glucopyranoside (3c).

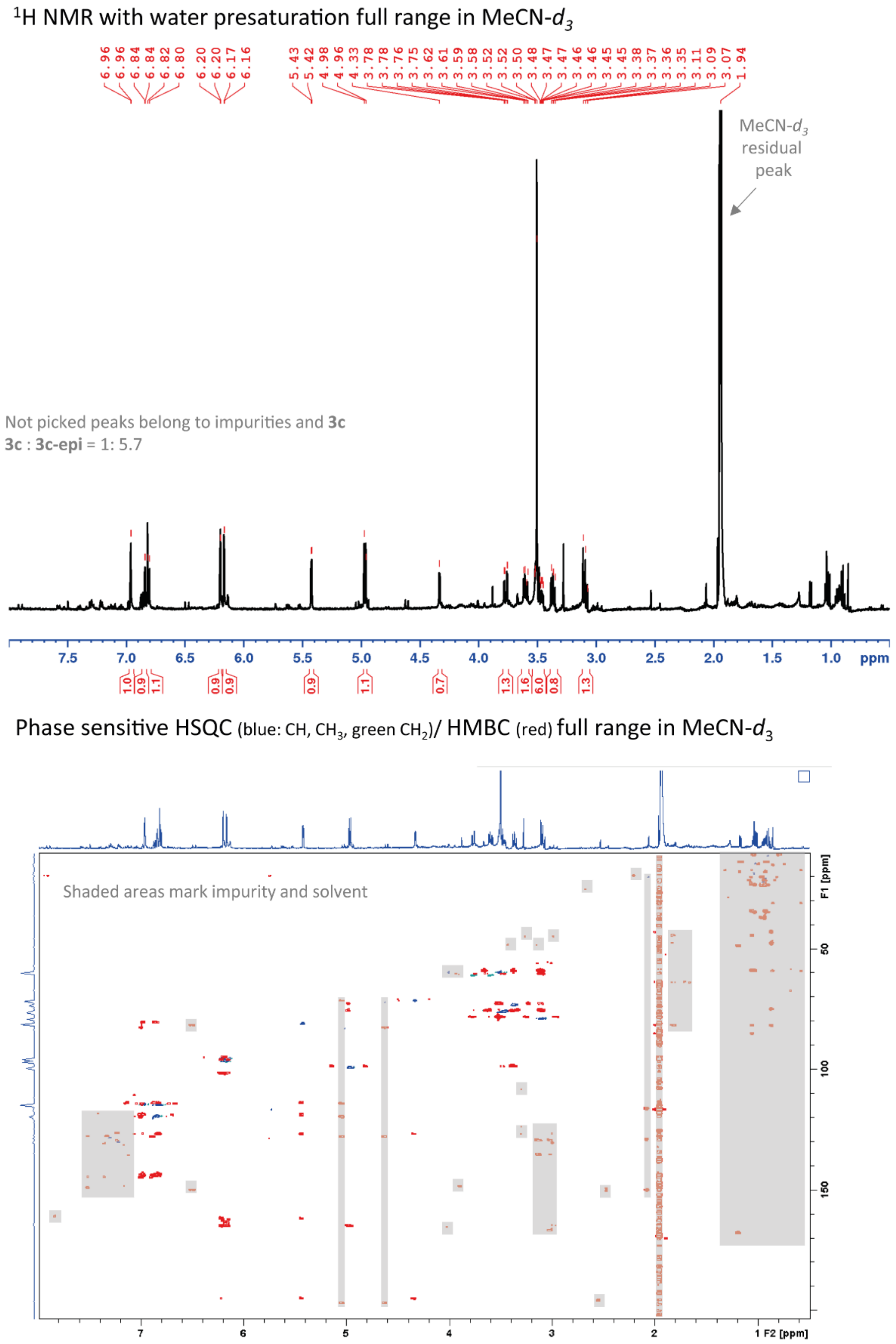

Fig. S3L NMR spectra for epitaxifolin-7-*O*-β-(4'-*O*-methyl)glucopyranoside (3c-epi).

^{1}H NMR with water presaturation full range in MeOH-d_3

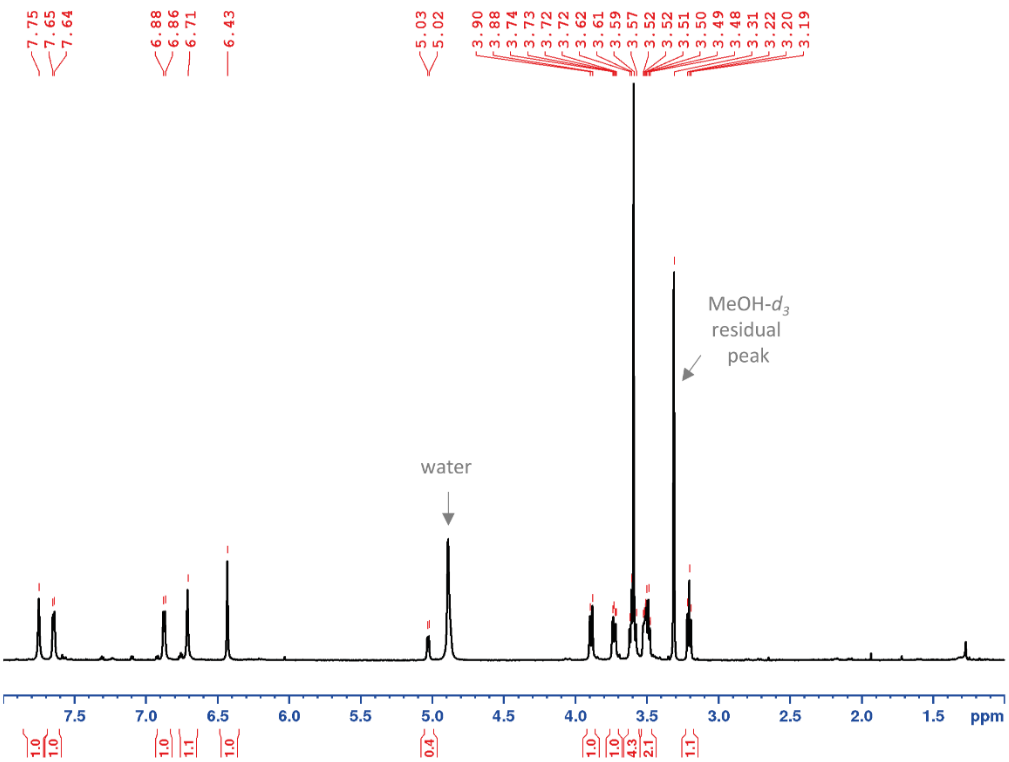

DEPTQ full range in MeOH-d_3

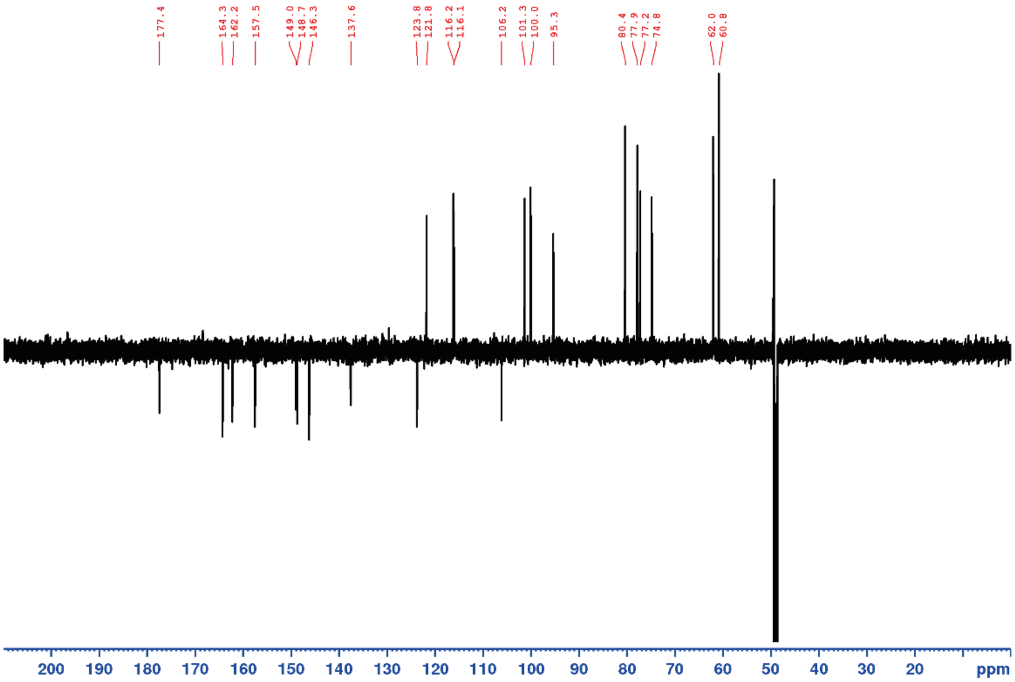

Fig. S3M NMR spectra for quercetin-7-*O*-β-(4'-*O*-methyl)glucopyranoside (3d).

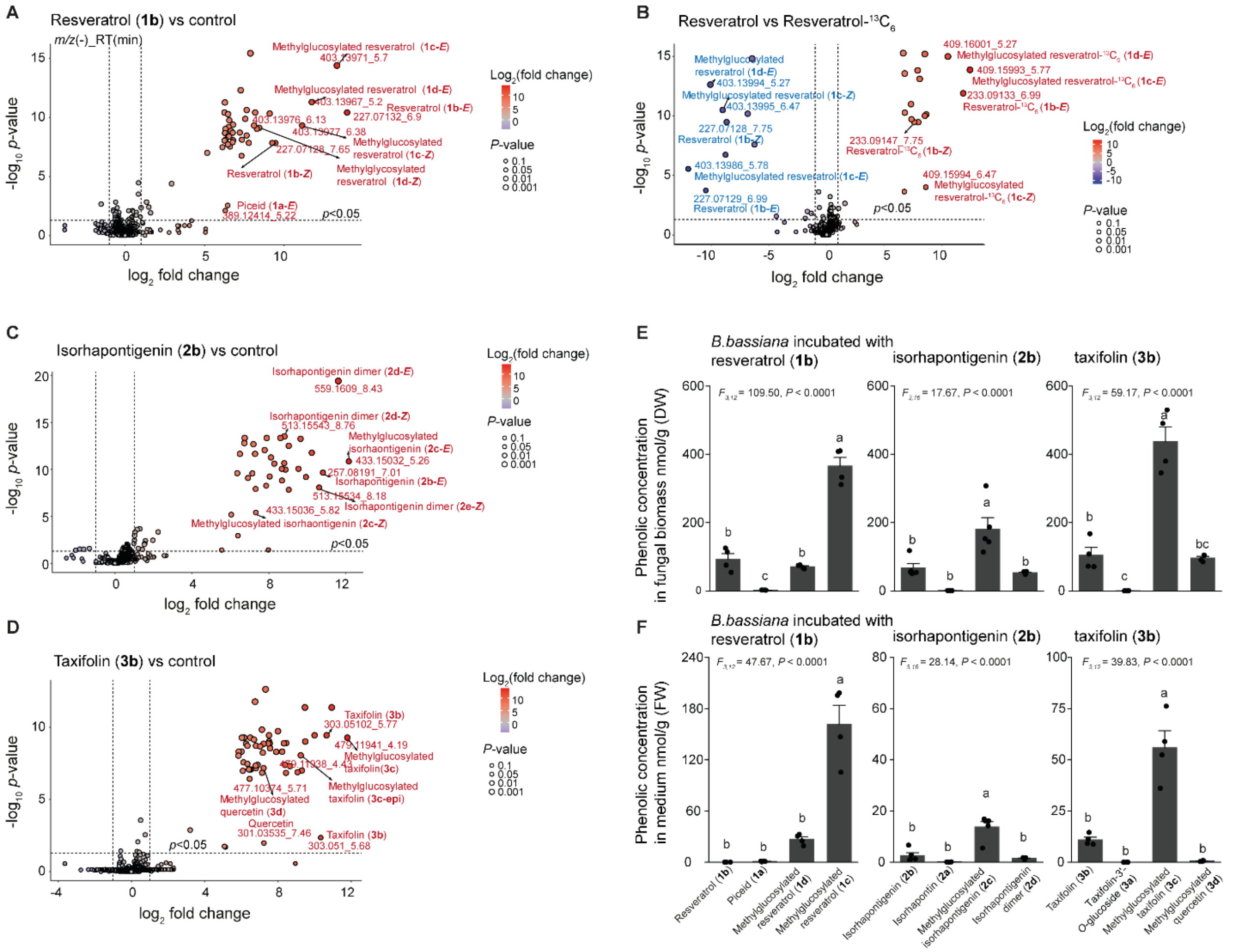

Fig. S4 *B. bassiana* metabolizes phenolic aglucones resulting in the formation of methylglucosylated phenolic products. (**A-D**) Volcano plots of metabolites of *B. bassiana* strain 01 incubated with resveratrol compared to control (**A**) and resveratrol-$^{13}C_6$ (**B**), as well as the volcano plots of metabolites of *B. bassiana* incubated with isorhapontigenin (**C**) and taxifolin (**D**) compared to a control (n = 4-5 per treatment). Data are based on peak intensity, normalized by sample weight and $\log_{10}$-transformed. Significant metabolites formed by *B. bassiana* metabolism of phenolic substances are listed in Supplementary Data file. (**E-F**) Targeted analyses of phenolic compounds and their derivative metabolites in fungal biomass (**E**) and the incubation medium (**F**) after incubating *B. bassiana* strain 02 with 200 µM resveratrol, isorhapontigenin, and taxifolin for 5 days (n = 4-5 per treatment). Statistically significant differences between means (±SE) were determined using two-sample t-tests in **A-D**, and one-way ANOVA followed by Tukey's HSD tests in **E** and **F**. Different lowercase letters denote statistically significant differences ($p < 0.05$).

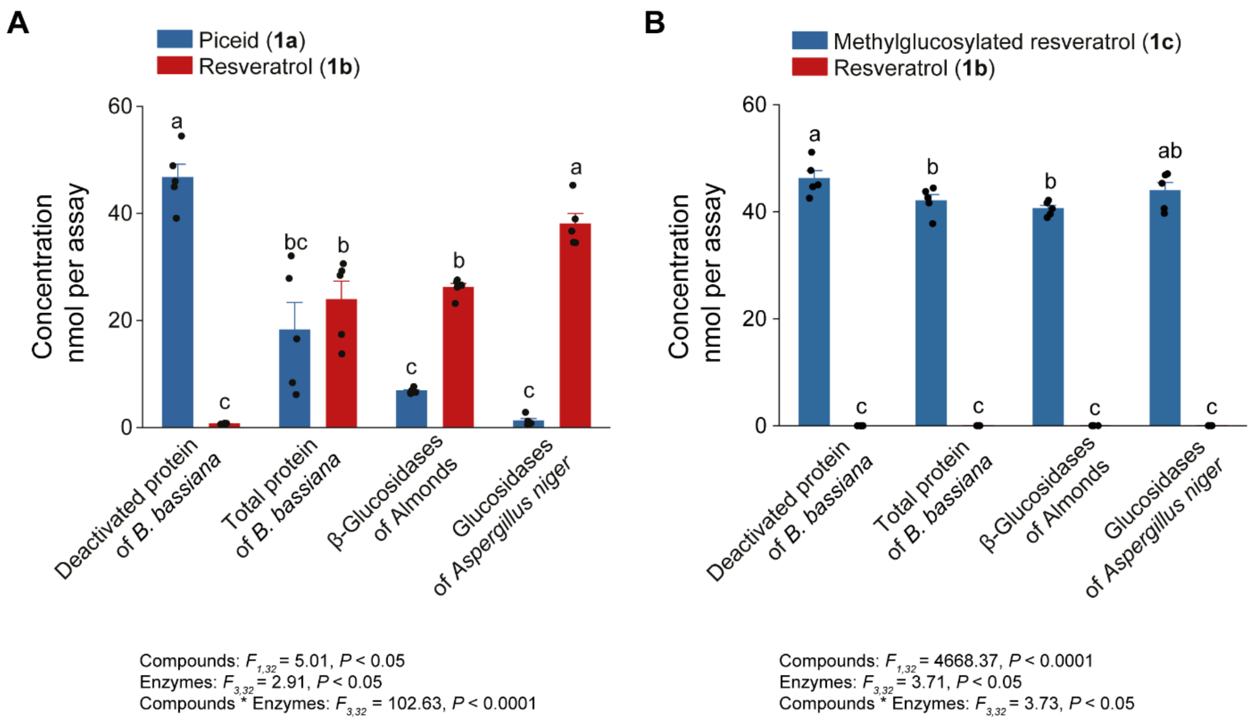

Fig. S5 Phenolic glucosides are hydrolyzed by β-glucosidase, but methylglucosylated phenolics are not. (**A-B**) In vitro enzyme assay using crude proteins from *B. bassiana* and commercial β-glucosidase from almonds or *Aspergillus niger* acting on piceid (**A**) and methylglucosylated resveratrol (**B**) (n = 5 per treatment). The remaining substrates piceid or methylglucosylated resveratrol and resveratrol formed were quantified. Significant differences between means (±SE) were determined using two-way ANOVA followed by Tukey's HSD tests. Different lowercase letters denote statistically significant differences ($p < 0.05$).

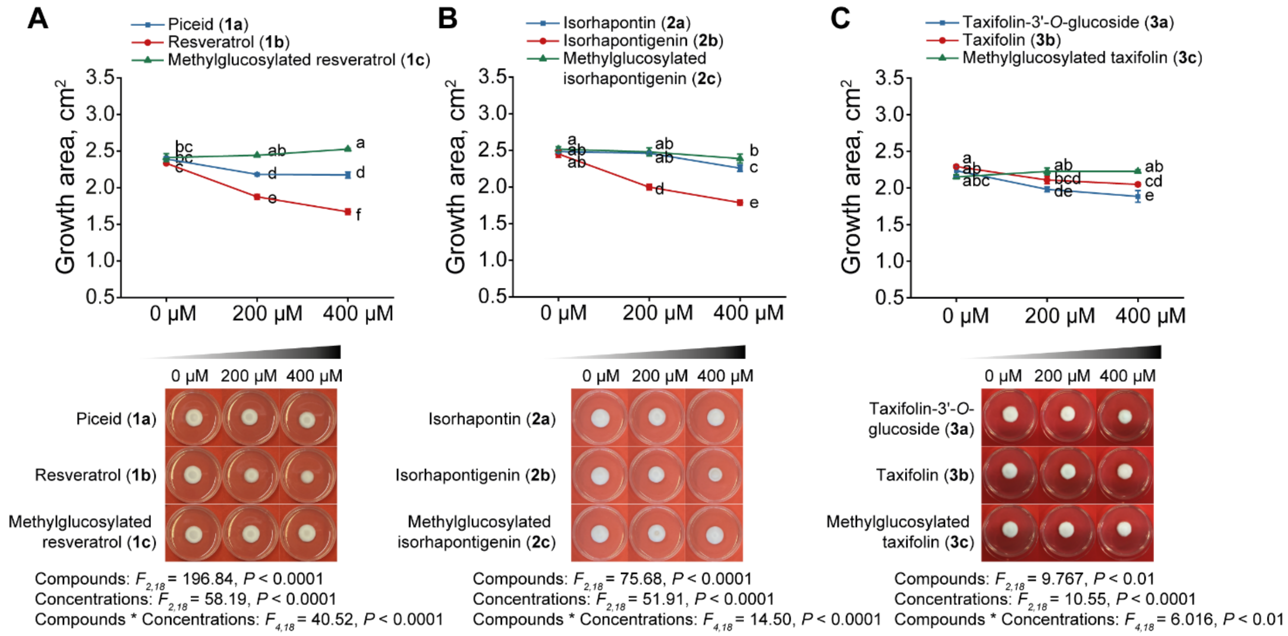

Fig. S6 The methylglucosylated phenolics formed in *B. bassiana* do not inhibit fungal growth. (**A**) Growth area of *B. bassiana* on PDA plates supplemented with increasing concentrations of piceid, resveratrol, and methylglucosylated resveratrol (n = 3). (**B**) Growth of *B. bassiana* on plates containing isorhapontin, isorhapontigenin, and methylglucosylated isorhapontigenin (n = 3). (**C**) Growth of *B. bassiana* on plates containing taxifolin-3'-*O*-glucoside, taxifolin, and methylglucosylated taxifolin (n = 3). Significant differences between means (±SE) were determined using two-way ANOVA followed by Fisher's tests. Different lowercase letters denote statistically significant differences ($p < 0.05$).

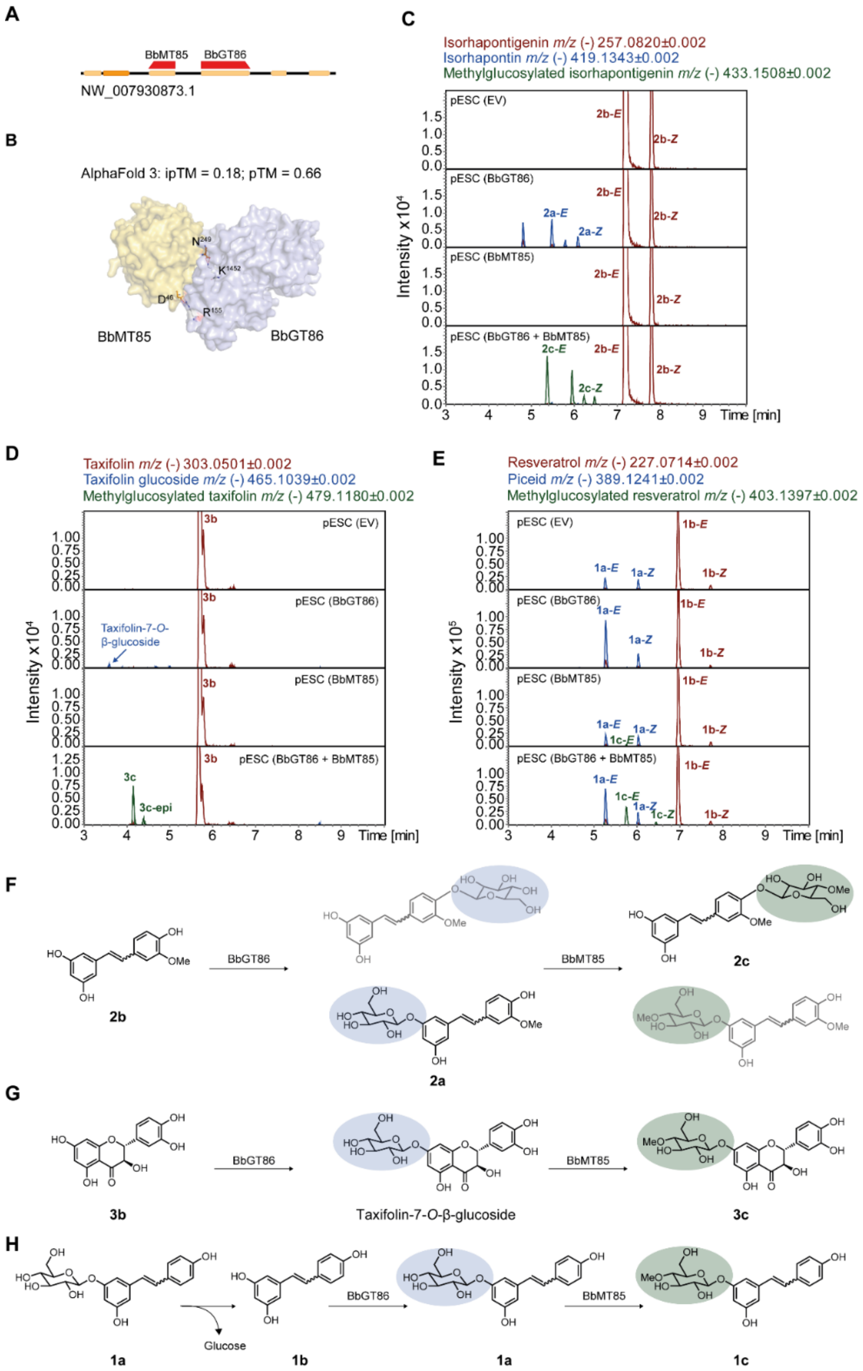

Fig. S7 Enzymatic activities of recombinant BbGT86 and BbMT85 proteins on phenolic substances. (**A**) The gene cluster of *B. bassiana gt86* and *mt85* that located in the genomic scaffold of NW_007930873.1 (*B. bassiana* ARSEF 2860 strain). (**B**) Protein model structure consisting of an overlay view of BbGT86 and BbMT85 using AlphaFold 3. The red dashed lines indicate the hydrogen bonds between BbGT86 and bMT85. (**C-E**) Extracted ion chromatograms in negative ionization mode measured by UHPLC-qTOF-MS showing the metabolites produced by yeast cells expressing empty vector (EV), recombinant BbGT86, BbMT85, or both BbGT86 and BbMT85 after reaction with isorhapontigenin (**C**), taxifolin (**D**), and piceid (**E**). Mass spectra of metabolite products and phenolic standards are available in Edmond. (**F-H**) Methylglucosylation pathways of *B. bassiana* on isorhapontigenin (**F**), taxifolin (**G**), and piceid (**H**) illustrate the metabolism of spruce tree derived phenolics by *B. bassiana*. The chemical structures in grey color hypothesized.

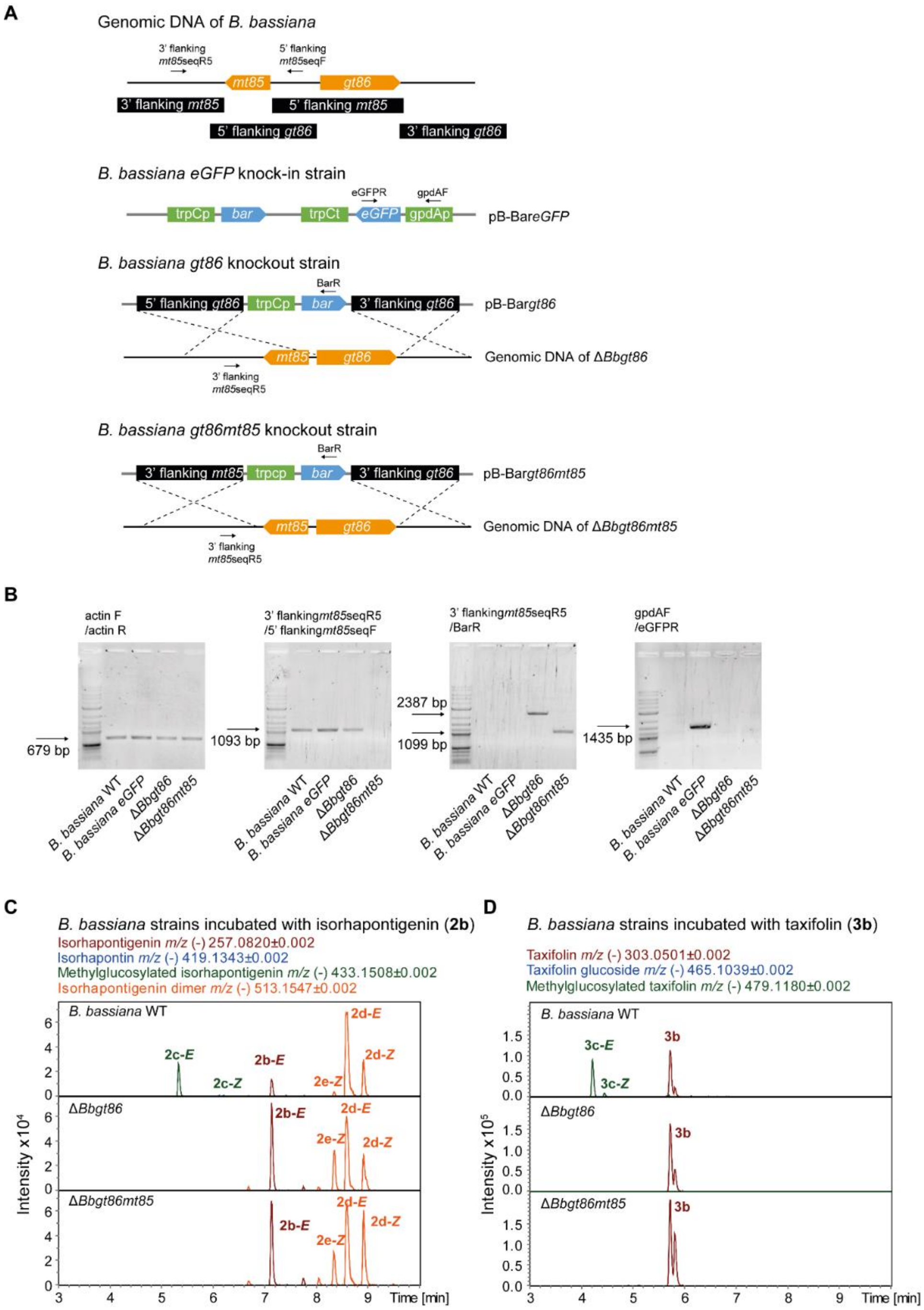

Fig. S8 *B. bassiana* gene knockouts via *Agrobacterium tumefaciens*-mediated transformation. (**A**) Schematic representation of constructed binary vectors used for *eGFP* gene knock-in, as well as those targeting knocking out of the *gt86* and both *gt86mt85* genes from *B. bassiana* genomic DNA via homologous recombination. Primer pairs were designed for genotyping to screen the *B. bassiana eGFP*, Δ*Bbgt86* and Δ*Bbgt86mt85* mutants from wild-type *B. bassiana*. (**B**) PCR results for the genotyping of *B. bassiana* WT, *B. bassiana eGFP*, Δ*Bbgt86*, and Δ*Bbgt86mt85* mutants. (**C-D**) Extracted ion chromatograms in negative ionization mode measured by UHPLC-qTOF-MS showing the metabolite products from *B. bassiana* WT, Δ*Bbgt86*, and Δ*Bbgt86mt85* mutants incubated with isorhapontigenin (**C**), and taxifolin (**D**). Mass spectra of metabolite products and phenolic standards are available in Edmond.

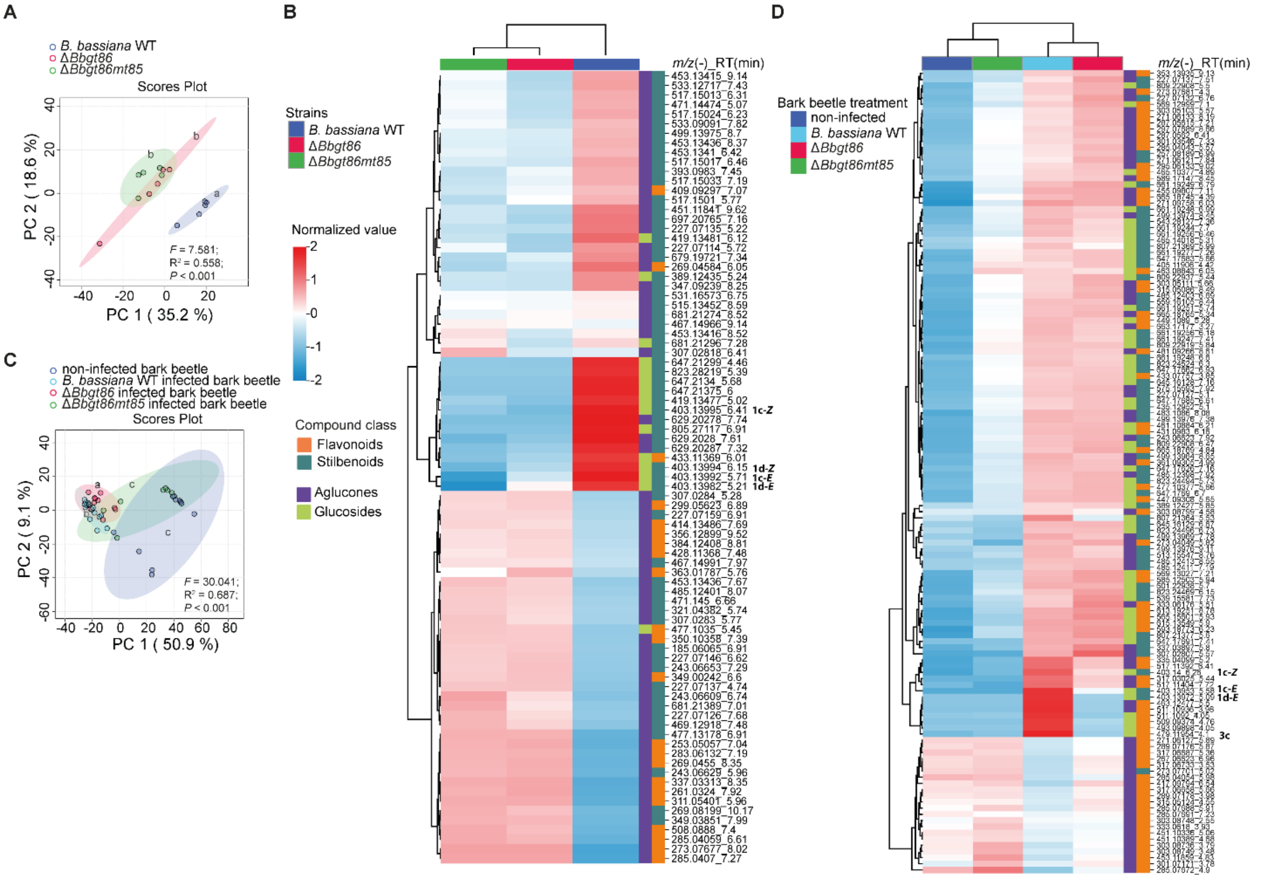

Fig. S9 Altered metabolic profiles in Δ*Bbgt86* and Δ*Bbgt86mt85* mutants relative to wild-type *B. bassiana*. (**A**) Principal component analysis (PCA) plots showing metabolite variations in *B. bassiana* WT, Δ*Bbgt86*, and Δ*Bbgt86mt85* mutants, incubated with 400 µM resveratrol (n = 5). (**B**) Heatmap displaying features of flavonoids and stilbenoids, derived from non-targeted UHPLC-qTOF-MS analyses (in negative mode) of *B. bassiana* WT, Δ*Bbgt86*, and Δ*Bbgt86mt85* mutants incubated with 400 µM resveratrol (n = 5). (**C**) PCA plot showing metabolite variations in bark beetle infected with *B. bassiana* WT, Δ*Bbgt86*, and Δ*Bbgt86mt85* mutants, as well as uninfected bark beetles. (**D**) Heatmap displaying features of flavonoids and stilbenoids, derived from non-targeted UHPLC-qTOF-MS analyses (in negative mode) of *B. bassiana* WT, Δ*Bbgt86*, and Δ*Bbgt86mt85* mutants infected bark beetles, and uninfected bark beetles (n = 15, 12, 8, and 10, respectively). Data are based on peak intensity, normalized by sample weight, and $\log_{10}$-transformed in (**B**) and (**D**). Detailed metabolomics results are listed in Supplementary Data files. Statistically significant differences between means (±SE) were determined using pairwise PERMANOVA in **A** and **C**, and one-way ANOVA followed by Fisher's LSD tests in **B** and **D**. Different lowercase letters denote statistically significant differences (p < 0.05).

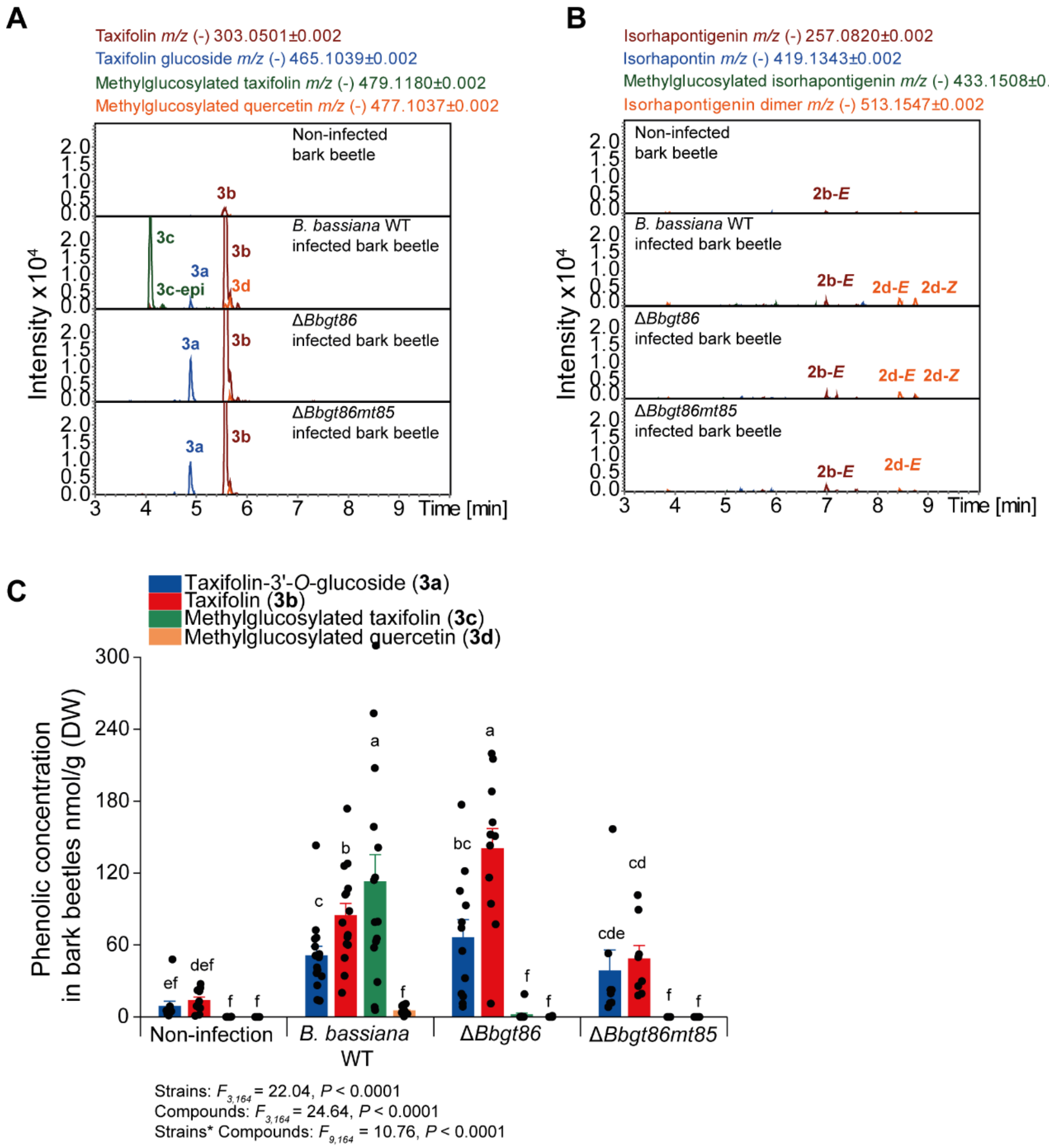

Fig. S10 Detoxification-related gene knockout blocked the methylglucosylated phenolics formation in *B. bassiana*-infected bark beetles. (**A-B**) Extracted ion chromatograms in negative ionization mode measured by UHPLC-qTOF-MS displaying the metabolite profiles of uninfected bark beetles, and *B. bassiana* WT, Δ*Bbgt86* and Δ*Bbgt86mt85* mutants infected bark beetles fed on the semi-artificial diet. The metabolites of taxifolin-3'-*O*-glucoside and isorhapontin in bark beetles are displayed in (**A**) and (**B**), respectively. Mass spectra of metabolite products and phenolic standards are available in Edmond. (**C**) Targeted analyses of metabolites of taxifolin-3'-*O*-glucoside in uninfected beetles and those infected with *B. bassiana* WT, Δ*Bbgt86*, and Δ*Bbgt86mt85* mutants, fed on the semi-artificial diet (n = 10, 15, 12, and 8, respectively). Statistically significant differences between means (±SE) were determined using two-way ANOVA followed by Fisher's LSD tests in **C.** Different lowercase letters denote statistically significant differences ($p < 0.05$).

Tables

Table S1 Phenolic content in the semi-artificial diet fed to bark beetle with or without *Beauveria bassiana* infection. Statistically significant differences were determined using Fisher's LSD tests with two-way ANOVA. N= 12, 10, and 15 in each treatment.

Treatment	Phenolic content in semi-artificial diet	Mean (nmol/g FW) ± SE	sig.
without bark beetle	Piceid (**1a**)	1227.59±145.88	b
	Resveratrol (**1b**)	262.05±52.47	f
	Methylglucosylated resveratrol (**1d**)	0.15±0.03	g
	Methylglucosylated resveratrol (**1c**)	0.04±0.01	g
	Isorhapontin (**2a**)	617.19±150.69	e
	Isorhapontigenin (**2b**)	17.80±4.48	g
	Methylglucosylated isorhapontigenin (**2c**)	0.00±0.00	g
	Isorhapontigenin dimer (**2d**)	0.54±0.12	g
	Taxifolin-3'-O-glucoside (**3a**)	1749.79±251.13	a
	Taxifolin (**3b**)	950.92±94.32	cd
	Methylglucosylated taxifolin (**3c**)	0.00±0.00	g
	Methylglucosylated quercetin (**3d**)	0.20±0.03	g
non-infected bark beetles	Piceid (**1a**)	923.90±158.13	cd
	Resveratrol (**1b**)	315.83±44.48	f
	Methylglucosylated resveratrol (**1d**)	0.18±0.04	g
	Methylglucosylated resveratrol (**1c**)	0.03±0.01	g
	Isorhapontin (**2a**)	324.63±36.46	f
	Isorhapontigenin (**2b**)	12.97±2.24	g
	Methylglucosylated isorhapontigenin (**2c**)	0.00±0.00	g
	Isorhapontigenin dimer (**2d**)	7.32±2.07	g
	Taxifolin-3'-O-glucoside (**3a**)	871.68±164.68	d
	Taxifolin (**3b**)	861.14±122.09	d
	Methylglucosylated taxifolin (**3c**)	0.00±0.00	g
	Methylglucosylated quercetin (**3d**)	0.26±0.05	g
B. bassiana WT infected bark beetle	Piceid (**1a**)	1119.52±104.32	bc
	Resveratrol (**1b**)	259.85±22.56	f
	Methylglucosylated resveratrol (**1d**)	0.47±0.26	g
	Methylglucosylated resveratrol (**1c**)	5.71±4.20	g
	Isorhapontin (**2a**)	339.69±37.79	f
	Isorhapontigenin (**2b**)	8.42±1.60	g
	Methylglucosylated isorhapontigenin (**2c**)	0.29±0.24	g
	Isorhapontigenin dimer (**2d**)	1.56±0.37	g
	Taxifolin-3'-O-glucoside (**3a**)	1025.73±125.53	bcd
	Taxifolin (**3b**)	916.70±77.99	d
	Methylglucosylated taxifolin (**3c**)	2.84±1.48	g
	Methylglucosylated quercetin (**3d**)	0.25±0.03	g

Table S2A NMR data for taxifolin-3'-*O*-β-glucopyranoside (3a).

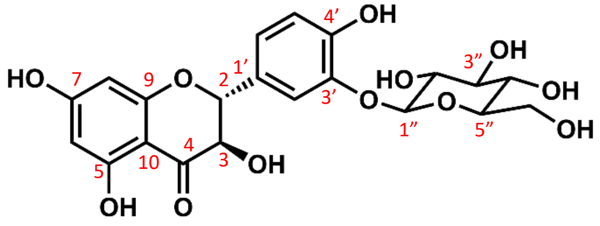

pos.	δ_H	mult.	J_{HH}	δ_C
2	4.97	*d*	11.6	84.8
3	4.57	*dd*	11.6/3.4	73.4
4	-	-	-	198.4
5	-	-	-	165.4
6	5.91	*d*	2.2	97.3
7	-	-	-	168.8
8	5.87	*d*	2.2	96.3
9	-	-	-	164.3
10	-	-	-	101.8
1'	-	-	-	129.9
2'	7.37	*d*	2.0	118.1
3'	-	-	-	146.5
4'	-	-	-	149.0
5'	6.89	*d*	8.3	116.8
6'	7.09	*dd*	8.3/2.0	124.5
1"	4.83	*d*	7.5	103.9
2"	3.52	*dd*	9.1/7.5	74.9
3"	3.47	*dd*	9.1/9.1	77.7
4"	3.36	*dd*	9.1/9.1	71.5
5"	3.43	*m*	-	78.3
6"a	3.90	*dd*	11.8/1.3	62.5
6"b	3.67	*dd*	11.8/5.7	62.5

500 MHz in MeOH-d_3

This compound was previously identified by Li S.H. *et al*. (2007) (12).

Table S2B NMR data for (*E*)-resveratrol-3-*O*-β-(4'-*O*-methyl)glucopyranoside (1c-*E*)

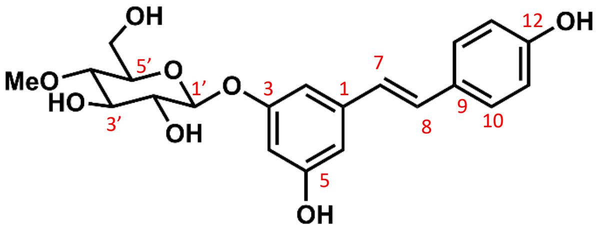

pos.	δ_H	mult.	J_{HH}	δ_C
1	-	-	-	141.3
2	6.75	*m*	-	106.9
3	-	-	-	160.3
4	6.42	*dd*	2.0/2.0	104
5	-	-	-	159.6
6	6.6	*m*	-	108.3
7	6.83	*d*	16.2	126.5
8	7	*d*	16.2	129.8
9	-	-	-	130.2
10	7.35	*bd**	8.5	128.8
11	6.75	*bd**	8.5	116.5
12	-	-	-	158.5
13	6.75	*bd**	8.5	116.5
14	7.35	*bd**	8.5	128.8
1'	4.86	*d*	7.8	102.1
2'	3.45	*dd*	8.7/7.8	75.1
3'	3.57	*dd*	9.2/8.7	78.1
4'	3.18	*dd*	9.2/9.2	80.6
5'	3.43	*m*	-	77.1
6'a	3.88	*dd*	12.1/1.7	62.1
6'b	3.72	*dd*	12.1/5.2	62.1
4'-OMe	3.58	*s*	-	60.8

*1,4-substituted aromatic ring, 700 MHz in MeOH-d_3

This compound was previously identified by Xie L. *et al* (2018) (13).

Table S2C NMR data for (*Z*)-resveratrol-3-*O*-β-(4'-*O*-methyl)glucopyranoside (1c-*Z*)

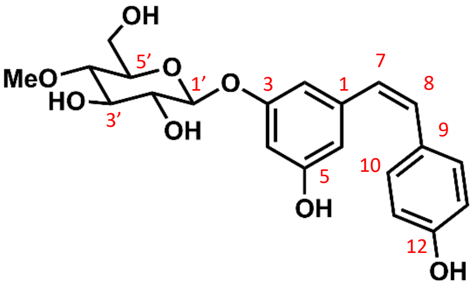

pos.	δ_H	mult.	J_{HH}	δ_C
1	-	-	-	140.9
2	6.49	*m*	-	108.8
3	-	-	-	159.9
4	6.35	*dd*	2.0/2.0	103.9
5	-	-	-	159.3
6	6.37	*m*	-	111.2
7	6.33	*d*	12.1	129.0
8	6.45	*d*	12.1	131.2
9	-	-	-	129.7
10	7.07	*bd**	8.5	131.3
11	6.63	*bd**	8.5	116.0
12	-	-	-	157.8
13	6.63	*bd**	8.5	116.0
14	7.07	*bd**	8.5	131.3
1'	4.62	*d*	7.8	102.1
2'	3.37	*dd*	8.5/7.8	74.9
3'	3.49	*dd*	8.5/8.5	77.8
4'	3.19	*m***	-	80.1
5'	3.17	*m***	-	76.6
6'a	3.70	*dd*	12.3/1.6	61.7
6'b	3.66	*dd*	12.3/3.4	61.7
4'-OMe	3.56	*s*	12.1/5.2	60.6

*1,4-substituted aromatic ring, ** overlapped signals J unresolved, 700 MHz in MeOH-d_3

Table S2D NMR data for (*E*)-resveratrol 12-*O*-β-(4'-*O*-methyl)glucopyranoside (1d-*E*)

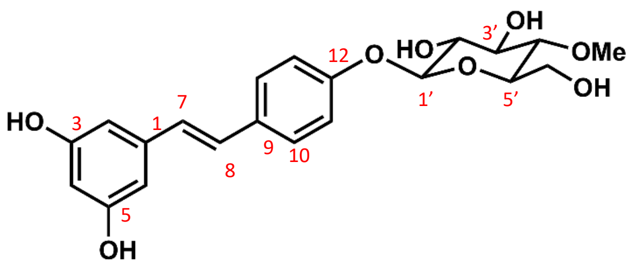

pos.	δ_H	mult.	J_{HH}	δ_C
1	-	-	-	140.8
2	6.45	*d*	2.1	105.9
3	-	-	-	159.7
4	6.16	*dd*	2.1/2.1	103.0
5	-	-	-	159.7
6	6.45	*d*	2.1	105.9
7	6.87	*d*	16.3	128.4
8	6.98	*d*	16.3	128.7
9	-	-	-	133.0
10	7.44	*bd**	8.9	128.4
11	7.06	*bd**	8.9	117.8
12	-	-	-	158.5
13	7.06	*bd**	8.9	117.8
14	7.44	*bd**	8.9	128.4
1'	4.90	*d*	7.9	101.9
2'	3.46	*dd*	8.4/7.9	75.0
3'	3.60	*m***	-	78.0
4'	3.20	*dd*	9.7/9.4	80.5
5'	3.43	*m***	-	77.1
6'a	3.86	*bd*	12.2	62.1
6'b	3.71	*m***	-	62.1
4'-OMe	3.58	*s*	-	60.8

*1,4-substituted aromatic ring, ** overlapped signals J unresolved, 500 MHz in MeOH-d_3

This compound was previously identified by Xie L. *et al* (2018) (13).

Table S2E NMR data for (*Z*)-resveratrol 12-*O*-β-(4'-*O*-methyl)glucopyranoside (1d-*Z*)

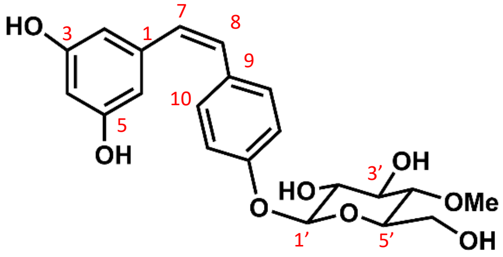

pos.	δ_H	mult.	J_{HH}	δ_C
1	-	-	-	140.7
2	6.18	*d*	1.8	108.1
3	-	-	-	159.4
4	6.10	*dd*	1.8/1.8	102.5
5	-	-	-	159.4
6	6.18	*d*	1.8	108.1
7	6.39	*d*	12.2	130.4
8	6.44	*d*	12.2	130.3
9	-	-	-	132.6
10	7.17	*bd**	8.6	131.1
11	6.92	*bd**	8.6	117.1
12	-	-	-	158.0
13	6.92	*bd**	8.6	117.1
14	7.17	*bd**	8.6	131.1
1'	4.84	*d*	7.8	101.86
2'	3.43	*dd*	9.0/7.8	75.0
3'	3.55	*dd*	9.0/9.0	78.0
4'	3.17	*dd*	9.1/9.0	80.5
5'	3.39	*ddd*	9.1/4.4/1.8	77.0
6'a	3.83	*dd*	12.2/1.8	62.0
6'b	3.68	*dd*	12.2/4.4	62.0
4'-OMe	3.57	*s*	-	60.7

*1,4-substituted aromatic ring, 700 MHz in MeOH-d_3

Table S2F NMR data for (*E*)-isorhapontigenin-12-*O*-β-(4'-*O*-methyl)glucopyranoside (2c-*E*)

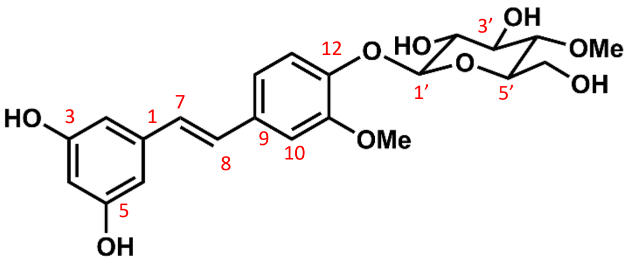

pos.	δ_H	mult.	J_{HH}	δ_C
1	-	-	-	140.8
2	6.47	*bs*	-	106.0
3	-	-	-	159.6
4	6.18	*bs*	-	103.1
5	-	-	-	159.7
6	6.47	*bs*	-	106.0
7	6.88	*d*	16.4	128.8
8	6.97	*d*	16.4	128.9
9	-	-	-	133.9
10	7.14	*bs*	-	111.1
11	-	-	-	150.8
12	-	-	-	147.5
13	7.09	*d*	8.4	117.7
14	7.02	*bd*	8.4	120.8
11-OMe	3.89	*s*	-	56.6
1'	4.87	*d*	7.9	102.4
2'	3.51	*dd*	8.3/7.9	75.0
3'	3.57	*m***	-	77.8
4'	3.20	*dd*	9.2/9.2	80.4
5'	3.38	*m*	-	77.1
6'a	3.83	*bd*	12.3	62.0
6'b	3.70	*dd*	12.3/4.0	62.0
4'-OMe	3.58	*s*	-	60.7

** overlapped signals J unresolved, 700 MHz in MeOH-d_3

Table S2G NMR data for (*Z*)-isorhapontigenin-12-*O*-β-(4'-*O*-methyl)glucopyranoside (2c-*Z*)

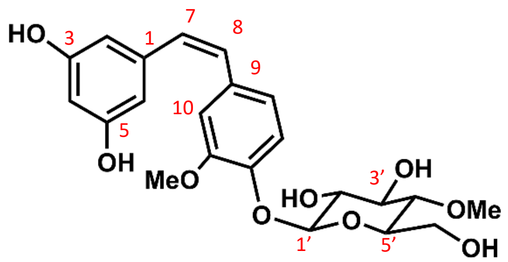

pos.	δ_H	mult.	J_{HH}	δ_C
1	-	-	-	140.5
2	6.25	*d*	1.8	108.2
3	-	-	-	159.0
4	6.13	*dd*	1.8/1.8	102.3
5	-	-	-	159.0
6	6.25	*d*	1.8	108.1
7	6.44	*d*	12.3	130.2
8	6.50	*d*	12.3	130.5
9	-	-	-	132.8
10	6.94	*d*	1.3	113.8
11	-	-	-	150.0
12	-	-	-	146.6
13	6.98	*d*	8.2	117.1
14	6.79	*dd*	8.2/1.3	122.7
11-OMe	3.63	*s*	-	56.2
1'	4.85	*d*	7.9	101.6
2'	3.37	*dd*	8.1/7.9	74.6
3'	3.50	*dd*	9.0/8.1	77.6
4'	3.10	*dd*	9.2/9.0	80.0
5'	3.36	*m*	-	76.8
6'a	3.73	*dd*	12.3/2.5	62.0
6'b	3.60	*dd*	12.3/5.3	62.0
4'-OMe	3.50	*s*	-	60.7

700 MHz in MeCN-d_3

Table S2H NMR data for shegansu B (2d-*E*)

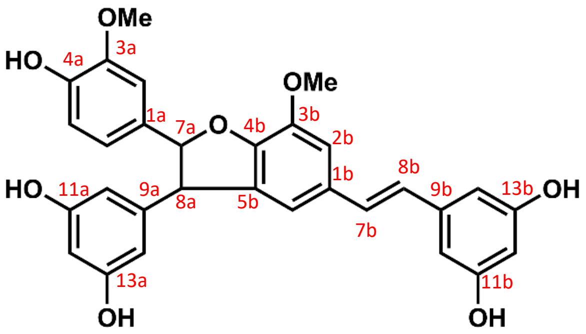

pos.	δ_H	mult.	J_{HH}	δ_C
1a	-	-	-	132.9
2a	6.90	*s*	-	110.6
3a	-	-	-	149.0
4a	-	-	-	147.8
5a	6.78	*m***	8.6	116.1
6a	6.78	*m***	8.6	120.1
7a	5.40	*d*	8.8	95.4
8a	4.42	*d*	8.8	59.0
9a	-	-	-	144.9
10a	6.13	*bd*	1.7	107.8
11a	-	-	-	159.9
12a	6.19	*dd*	1.7/1.7	102.6
13a	-	-	-	159.9
14a	6.13	*bd*	1.7	107.8
1b	-	-	-	133.2
2b	7.07	*bs*	-	111.5
3b	-	-	-	145.6
4b	-	-	-	149.2
5b	-	-	-	133.0
6b	6.79	*m***	-	116.9
7b	6.95	*d*	16.3	129.4
8b	6.80	*d*	16.3	127.7
9b	-	-	-	140.9
10b	6.43	*d*	1.7	105.8
11b	-	-	-	159.6
12b	6.15	*dd*	1.7/1.7	102.8
13b	-	-	-	159.6
14b	6.43	*d*	1.7	105.8
3a-OMe	3.80	*s*	-	56.2
3b-OMe	3.92	*s*	-	56.6

** overlapped signals J unresolved, 700 MHz in MeOH-d_3

This compound was previously identified by Li X.M.*et al* (2003) (14).

Table S2I NMR data for (*Z*)-shegansu B (2d-*Z*)

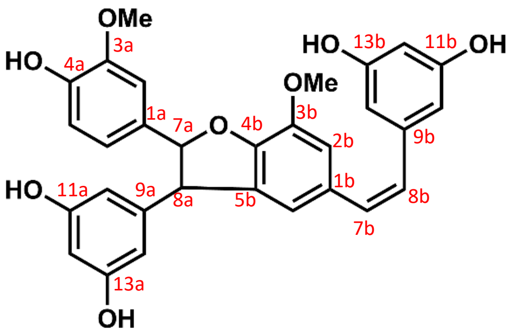

pos.	δ_H	mult.	J_{HH}	δ_C
1a	-	-	-	132.9
2a	6.88	*bs***	-	110.6
3a	-	-	-	149.0
4a	-	-	-	147.8
5a	6.76	*m***	-	116.1
6a	6.76	*m***	-	120.1
7a	5.34	*d*	8.8	95.3
8a	4.36	*d*	8.8	59.0
9a	-	-	-	144.76
10a	6.07	*d*	2.1	107.7
11a	-	-	-	159.8
12a	6.16	*dd*	2.1/2.1	102.5
13a	-	-	-	159.8
14a	6.07	*d*	2.1	107.7
1b	-	-	-	132.3
2b	6.88	*m***	-	113.7
3b	-	-	-	144.79
4b	-	-	-	148.6
5b	-	-	-	132.1
6b	6.51	*bs*	-	119.9
7b	6.41	*d*	12.2	131.0
8b	6.35	*d*	12.2	129.6
9b	-	-	-	140.0
10b	6.25	*d*	2.0	108.3
11b	-	-	-	159.4
12b	6.13	*dd*	2.0/2.0	102.54
13b	-	-	-	159.4
14b	6.25	*d*	2.0	108.3
3a-OMe	3.80	*s*	-	56.2
3b-OMe	3.64	*s*	-	56.1

** overlapped signals J unresolved, 700 MHz in MeOH-d_3

This compound was previously identified by Yao C.S. *et al* (2005) (15).

Table S2J NMR data for (*Z*)-bisisorhapontigenin A (2e-*Z*)

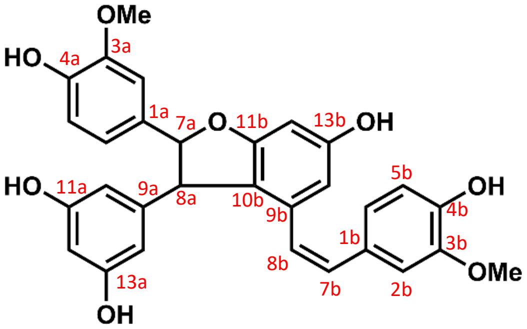

pos.	δ_H	mult.	J_{HH}	δ_C
1a	-	-	-	134.0
2a	6.69	*d*	1.6	110.2
3a	-	-	-	148.9
4a	-	-	-	147.6
5a	6.70	*d*	8.1	116.1
6a	6.54	*dd*	8.1/1.6	119.9
7a	5.20	*d*	7.0	94.9
8a	3.78	*d*	7.0	57.8
9a	-	-	-	146.8
10a	5.93	*d*	2.1	107.4
11a	-	-	-	159.5
12a	6.09	*dd*	2.1/2.1	101.9
13a	-	-	-	159.5
14a	5.93	*d*	2.1	107.4
1b	-	-	-	130.2
2b	6.61	*d*	1.5	112.7
3b	-	-	-	148.2
4b	-	-	-	146.9
5b	6.609	*d*	8.2	135.7
6b	6.56	*dd*	8.2/1.5	123.3
7b	6.18	*d*	12.1	131.7
8b	6.05	*d*	12.1	126.6
9b	-	-	-	137.9
10b	-	-	-	120.4
11b	-	-	-	162.7
12b	6.24	*d*	1.6	96.6
13b	-	-	-	154.8
14b	6.26	*d*	1.6	109.0
3a-OMe	3.76	*s*	-	56.2
3b-OMe	3.53	*s*	-	55.7

700 MHz in MeOH-d_3

Table S2K NMR data for taxifolin-7-*O*-β-(4'-*O*-methyl)glucopyranoside (3c)

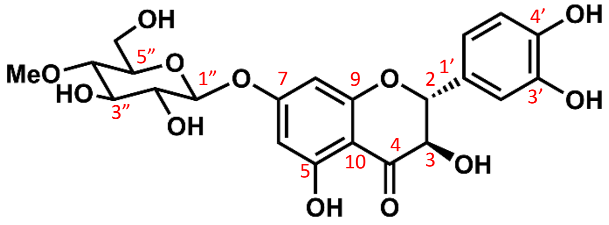

pos.	δ_H	mult.	J_{HH}	δ_C
2	5.03	*d*	11.9	84.4
3	4.61	*d*	11.9	73.2
4	-	-	-	198.7
5	-	-	-	164.5
6	6.19	*d*	2.2	97.9
7	-	-	-	166.8
8	6.13	*d*	2.2	96.6
9	-	-	-	164.0
10	-	-	-	103.2
1’	-	-	-	129.7
2’	6.98	*d*	1.5	115.9
3’	-	-	-	145.5
4’	-	-	-	146.4
5’	6.87	*d*	8.1	116.1
6’	6.89	*dd*	8.1/1.5	121.4
1”	4.95	*d*	7.8	100.4
2”	3.56	*dd*	9.2/7.8	74.3
3”	3.49	*dd*	9.2/9.0	77.3
4”	3.09	*dd*	9.7/9.0	79.9
5”	3.45	*ddd*	9.7/5.0/2.3	77.0
6”a	3.74	*dd*	11.9/2.3	62.0
6”b	3.58	*dd*	11.9/5.0	62.0
4'-OMe	3.50	*s*	-	60.8

500 MHz in MeCN-d_3

Table S2L NMR data for epitaxifolin-7-*O*-β-(4'-*O*-methyl)glucopyranoside (3c-epi)

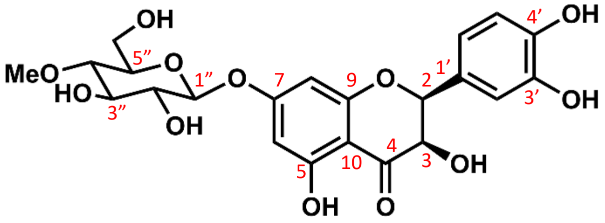

pos.	δ_H	mult.	J_{HH}	δ_C
2	5.42	*d*	3.1	81.0
3	4.33	*d*	3.1	71.6
4	-	-	-	195.8
5	-	-	-	164.0
6	6.17	*d*	2.2	96.7
7	-	-	-	165.5
8	6.20	*d*	2.2	95.6
9	-	-	-	162.5
10	-	-	-	102.2
1’	-	-	-	127.4
2’	6.96	*d*	1.8	114.6
3’	-	-	-	144.3
4’	-	-	-	144.8
5’	6.81	*d*	8.3	115.0
6’	6.85	*dd*	8.3/1.8	119.5
1”	4.97	*d*	7.8	99.3
2”	3.37	*dd*	9.1/7.8	73.3
3”	3.50	*m***	-	76.3
4”	3.09	*dd*	9.7/9.1	78.9
5”	3.47	*ddd*	9.7/5.3/2.3	76.1
6”a	3.77	*dd*	12.0/2.3	62.0
6”b	3.60	*dd*	12.0/5.3	62.0
4'-OMe	3.50	*s*	-	59.7

500 MHz in MeCN-d_3

Table S2M NMR data for quercetin-7-*O*-β-(4'-*O*-methyl)glucopyranoside (3d)

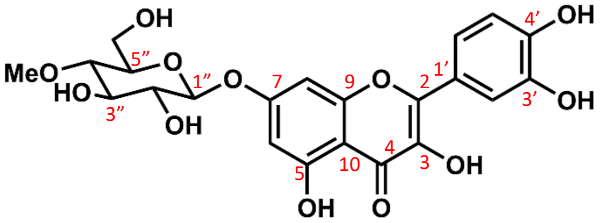

pos.	δ_H	mult.	J_{HH}	δ_C
2	-	-	-	148.7
3	-	-	-	137.6
4	-	-	-	177.4
5	-	-	-	162.2
6	6.43	*brs*	-	100.0
7	-	-	-	164.3
8	6.71	*brs*	-	95.3
9	-	-	-	157.5
10	-	-	-	106.2
1’	-	-	-	123.8
2’	7.75	*brs*	-	116.1
3’	-	-	-	146.3
4’	-	-	-	149.0
5’	6.87	*d*	8.1	116.2
6’	7.65	*brd*	8.1	121.8
1”	5.03	*d*	7.6	101.3
2”	3.49	*dd*	8.3/7.6	74.8
3”	3.61	*dd*	9.0/8.3	78.9
4”	3.20	*dd*	9.3/9.0	80.4
5”	3.51	*m*	-	77.2
6”a	3.89	*brd*	11.9	62.0
6”b	3.73	*dd*	11.9/4.6	62.0
4'-OMe	3.59	*s*	-	60.8

700 MHz in MeOH-d_3

This compound was previously identified by Zhan J. *et al* (2006) (16).

Table S3 Primers for gene cloning, qPCR, protein expression, and gene modification.

Name	Primer (5’-->3’)	Restriction site	Function
ITS4	TCCTCCGCTTATTGATATGC		internal transcribed spacer (ITS) region
ITS5	GGAAGTAAAAGTCGTAACAAGG		
IpsBbaactinQF	CCCAAGTCCAACCGTGAGAA		qPCR primer pairs of *Beauveria bassiana*
IpsBbaactinQR	GTGAGGAAGAGCGAAACCCT		
IpsBbaGT86QF	AAAGGGCTTCACTAGACGGC		
IpsBbaGT86QR	ACCTTGGGGTGGATATCGGA		
IpsBbaGT2-QF	AAATGGCCCGAGGACCTA		
IpsBbaGT2-QR	CAACTTGCTTGAGCGCAC		
IpsBbaGT3-QF	GCACGTGGCGTATCAGAA		
IpsBbaGT3-QR	TACGACGGAGCCTGACTT		
IpsBbaGT4QF	GGATTTCCCCCTCAAGAGCC		
IpsBbaGT4QR	TCAAAAGCAGCCCGAATCCT		
IpsBbaGT5QR	TCCATCCAAGCAACGTCCTC		
IpsBbaGT5QF	CACCGATGCCAAACGACTTG		
IpsBbaGT6QR	CTTCTGCGCTTCAGGTCGTA		
IpsBbaGT6QF	GTATCGGTGGCCTGATCCTG		
IpsBbaGT7QR	CGGTATCCTCTGCTCCGTTC		
IpsBbaGT7QF	ACGTTTCTGCCGTCGTACAT		
IpsBBaMT85-QR	AGGAAATCCGACAGCCGTTT		
IpsBbaMT85-QF	TCATAGTCGATGCTGGTGGC		
IpsBbaMT2-QR	GCCGAGCAGATCCTGAAACT		
IpsBbaMT2-QF	CTTTGGCGACCATTGCTACG		
IpsBbaMT3-QF	AAGGCGCTCGAATTGCAT		
IpsBbaMT3-QR	CAAGTGCCTTCCAGTCGA		
IpsBbaMT4-QF	TGCGCACCTTTTACCTCG		
IpsBbaMT4-QR	ACATCGATCACGGTCCAC		
IpsBbaMT3-2QR	CGCGCCGACAATCATTTG		
IpsBbaMT3-2QF	TTTTGCGCCGTTCATCAC		
IpsBbaMT3-3QR	TCGATGCTGTCAATGACGG		
IpsBbaMT3-3QF	ACCGCGACTTTAACGACG		
IpsBbaMT3-4QR	TCTTTGAGGGCCGCAATT		
IpsBbaMT3-4QF	TCAACACCGTCCTCAGCT		
IpsBbaMT3-5QR	ATCGCTTTCTCGGTTCGC		
IpsBbaMT3-5QF	TCAACATCGCATACCGCA		
IpsBbaMT3-6QF	ACATCCCGAAGCAACGTCTT		
IpsBbaMT3-6QR	CCACGCAAACAAAGCCATGA		
RPS3-a QF	GCCCCTTCAATGTTTGCCAC		qPCR primer pair of *Ips typographus*
RPS3-a QR	AAGTCCGCCAAACTAACCTCA		
IpsBbaGT86-FLF	ATGTCTCCGTCACGCAGC		open reading frame cloning
IpsBbaGT86-FLR	TCAATCCATAGCCGCCCAC		
IpsBbaMT85-FLF	ATGGCTCTCGTCGAAAAAATTCA		
IpsBbaMT85-FLR	TCAGTTTCTTTTTGCGATCAACATG		
pESCIpsBbaGT86vF	ATAATAGCGGCCGCAATGTCTCCGTCACGCAGC	NotI	fragment cloning for inserting into pESC-Leu vector
pESCIpsBbaGT86vR	GCGGCGTTAATTAATCAATCCATAGCCGCCCAC	PacI	
pESCIpsBbaMT85vF	GCGGGATCCATAATGGCTCTCGTCGAA	BamHI	
pESCIpsBbaMT85vR	GCGCCCGGGTCAGTTTCTTTTTGC	SamI	

Name	Primer (5’-->3’)	Function
5’ flanking MT85F	TCAATCCATAGCCGCCCAC	5' flanking region and 3' flanking region cloning from gDNA
5’ flanking MT85R	CTGCCCGCAAATCTAACT	
3’ flanking MT85F/5’ flanking GT86MT85R	GGGATGTCTATTTAGCCAATCA	
3’ flanking MT85R/5’ flanking GT86MT85F	AGCCCGCTGTTTCACATG	
5’ flanking GT86F	CAATAGACTCCAGTGAACCTT	
5’ flanking GT86R	TTGACTTTTGCGCCCACT	
3’ flanking GT86F/3’ flanking GT86MT85F	TCTACTAGGGCGGCAC	
3’ flanking GT86R/3’ flanking GT86MT85R	GCAAAGCTCGGGGGAAAA	
pBar-5’ flanking MT85vF	ACAGATTTTATGTTTAGATCCACTAGTTCAATCCATAGCCGCCCA	5’ flanking region and 3’ flanking region cloning for inserting into pBarGPE1 vector
pBar-5’ flanking MT85vR	CATGTAATGCATAGTACCGAGAACTGCCCGCAAATCTAACT	
pBar-3’ flanking MT85vF	CCTTCAATATCATCTTCTGTCGAGTTGACTTTTGCGCCCACTTG	
pBar-3’ flanking MT85vR	GAGCACAGAGGCCGCAGAATGTGCTCTAGAAGCCCGCTGTTTCACATG	
pBar-5’ flanking GT86vF	CACAGAGGCCGCAGAATGTGCTCTAGACAATAGACTCCAGTGAACCTT	
pBar-5’ flanking GT86vR	GTGCTCCTTCAATATCATCTTCTGTCGAGTCGCCAATCTCATTCGGGAG	
pBar-3’ flanking GT86vF	GTAATAATTAACATGTAATGCATAGTACCGAGAACATCGTCCTCGCGCTAAT	
pBar-3’ flanking GT86vR	GTTATTTTACAGATTTTATGTTTAGATCCACTAGTTACAGAGCGATCATGAAGC	
pBar-5’ flanking GT86MT85vF	CGCCAAGCTTGCATGCCTGCAGCAATAGACTCCAGTGAACCTT	
pBar-5’ flanking GT86MT85vR	TCCTTCAATATCATCTTCTGTCGAGGGGATGTCTAATTAGCCAATCA	
pBar-3’ flanking GT86MT85vF	CTGCCCGTCACCGAGATCTGATCTACTAGGGCGGCACAGATAACAAT	
pBar-3’ flanking GT86MT85vR	CGACGGCCAGTGAATTCGAGCTCAAGTACTTTACTGTCCCAGACGC	
pB-BarGT86MT85vF	CATGATTACGAATTCGAGCTCGGTACCCAAGTACTTTACTGTCCCAGACGC	inserting homologous recombination cassettes into pCAMBIAXmnI vector
pB-BarGT86MT85vR	CTGCAGGTCGACTCTAGAGGATCCCCCAATAGACTCCAGTGAACCTTGC	
pB-BarGT86vF	CATGATTACGAATTCGAGCTCGGTACCCAAGTACTTTACTGTCCCAGACGC	
pB-BarGT86vR	CTTGCATGCCTGCAGGTCGACTCTAGAGGATCCCCCTGAATATTTTCTGCCGC	
pB-BarMT85vF	CTATGACCATGATTACGAATTCGAGCTCGGTACCCTGGCCACTCATGTTCCCT	
pB-BarMT85vR	CTTGCATGCCTGCAGGTCGACTCTAGAGGATCCCCTCAATCCATAGCCGCCCAC	
pB-BareGFPvF	CATGATTACGAATTCGAGCTCGGTACCCTCGACAGAAGATGATATTGAAG	
pB-BareGFPvR	CTTGCATGCCTGCAGGTCGACTCTAGAGGATCCCCGCATGCGGAGAGACGGAC	
Bba-actinF	AGATTTGGCACCACACCT	genotyping
Bba-actinR	GCAAGACCAGGGTACATG	
3’ flankingMT85seqR5	CTCGGGCAATTGTGGTCA	
5’ flankingMT85seqF	AGTTAGATTTGCGGGCAG	
egfp R	ACTCCAGCAGGACCATGT	
BarR	TCCAGCTGCCAGAAACC	
gpdAF	GCATGCGGAGAGACGGAC	

Table S4 The accession numbers of genes

Species	Gene name	Type	Accession number	Isolate
Beauveria bassiana	Internal transcribed spacer (ITS)	rRNA	PV798168	*B. bassiana* MPICE01 (*B. bassiana* 01)
		rRNA	PV799956	*B. bassiana* MPICE02 (*B. bassiana* 02)
	UDP-glycosyltransferase 1 (GT86)	mRNA	XM_008603783.1	*B. bassiana* ARSEF 2860
		mRNA	PV799293	*B. bassiana* MPICE01 (*B. bassiana* 01)
		Protein	XP_008602005.1	*B. bassiana* ARSEF 2860
	O-methyltransferase 1 (MT85)	mRNA	XM_008603782.1	*B. bassiana* ARSEF 2860
		mRNA	PV799292	*B. bassiana* MPICE01 (*B. bassiana* 01)
		Protein	XP_008602004.1	*B. bassiana* ARSEF 2860
	actin	mRNA	XM_008599957.1	*B. bassiana* ARSEF 2860
	MT85-GT86 located genome	DNA	NW_007930873.1	
	GT2	mRNA	XM_008598680.1	
		Protein	XP_008596902.1	
	GT3	mRNA	XM_008598682.1-	
		Protein	XP_008596904.1	
	GT4	mRNA	XM_008596109.1	
		Protein	XP_008594331.1	
	GT5	mRNA	XM_008599438.1	
		Protein	XP_008597660.1	
	GT6	mRNA	XM_008596647.1	
		Protein	XP_008594869.1	
	GT7	mRNA	XM_008601439.1	
		Protein	XP_008599661.1	
	MT2	mRNA	XM_008598677.1	
		Protein	XP_008596899.1	
	MT3	mRNA	XM_008598679.1	
		Protein	XP_008596901.1	
	MT4	mRNA	XM_008604595.1	
		Protein	XP_008602817.1	
	MT3-2	mRNA	XM_008604978.1	
		Protein	XP_008603200.1	
	MT3-3	mRNA	XM_008596031.1	
		Protein	XP_008594253.1	
	MT3-4	mRNA	XM_008599053.1	
		Protein	XP_008597275.1	
	MT3-5	mRNA	XM_008600489.1	
		Protein	XP_008598711.1	
	MT3-6	mRNA	XM_008604841.1	
		Protein	XP_008603063.1	
Ips typographus	40S ribosomal protein S3-A *(RPS3-α)*	mRNA	Ityp04549 in Sellamuthu,G., *et al.*, (2022) (11)	

Table S5 HPLC-MS/MS parameters used for multiple reaction monitoring (MRM) analyses on the LC-MS/MS system in negative ionisation mode. Q1, selected *m/z* of the first quadrupole; Q3, selected *m/z* of the third quadrupole; DP, declustering potential; EP, entrance potential; CE, collision energy; and CXP, collision cell exit potential

Metabolites	Ionization mode	Q1 *m/z*	Q3 *m/z*	Retention time (min)	DP (V)	EP (V)	CE (V)	CXP (V)
Taxifolin (**3b**)	negative	303	125	4	-60	-8	-28	-10
Piceid (**1a**)		389	227	3.5	-60	-8	-38	-10
Isorhapontin (**2a**)		419	257	4.8	-60	-8	-18	-10
Taxifolin-3'-O-glucoside (**3a**)		465	285	3.3	-60	-8	-44	-10
Resveratrol (**1b**)		227	185	5.5	-60	-8	-28	-10
Isorhapontigenin (**2b**)		257	241	5.5	-60	-8	-28	-10
Methylglucosylated isorhapontigenin (**2c**)		433	257	4.3	-60	-8	-22	-10
Methylglucosylated resveratrol (**1c**)		403	227	4.3	-60	-8	-32	-10
Methylglucosylated resveratrol (**1d**)		403	227	3.9	-60	-8	-30	-10
Isorhapontigenin dimer (**2d**)		513	497	6.8	-60	-8	-30	-10
Methylglucosylated taxifolin (**3c**)		479	303	3.2	-60	-8	-20	-10
Methylglucosylated quercetin (**3d**)		477	301	4.5	-60	-8	-44	-10

Table S6 Chemical standards and compound information

Compounds	The other name	Supplier	CAS number
Piceid		TCI, Eschborn, Germany	27208-80-6
Resveratrol		TCI	501-36-0
Taxifolin		TCI	480-18-2
Isorhapontigenin		TCI	32507-66-7
Piceatannol		TCI	10083-24-6
Isorhapontin		Polyphenols AS, Sandnes, Norway	32727-29-0
Astringin		Polyphenols	29884-49-9
Quercetin		Sigma,Taufkirchen, Germany	117-39-5
Quercetin-3'-*O*-glucoside		MedChemExpress，Sollentuna, Sweden	19254-30-9
Taxifolin-7-*O*-glucoside		MedChemExpress	14292-40-1
Taxifolin-3'-*O*-glucoside	dihydroxyquercetin -3'-*O*-β-glucopyranoside	Isolated from spruce bark	
Methylglucosylated resveratrol (**1d-*E***)	(*E*)-resveratrol 12-*O*-β-(4'-*O*-methyl)glucopyranoside	Isolated from *B. bassiana* fungi on PDA plates with phenolic aglucones	
Methylglucosylated resveratrol (**1d-*Z***)	(*Z*)-resveratrol 12-*O*-β-(4'-*O*-methyl)glucopyranoside		
Methylglucosylated resveratrol (**1c-*E***)	(*E*)-resveratrol 3-*O*-β-(4'-*O*-methyl)glucopyranoside		
Methylglucosylated resveratrol (**1c-*Z***)	(*Z*)-resveratrol 3-*O*-β-(4'-*O*-methyl)glucopyranoside		
Methylglucosylated isorhapontigenin (**2c-*E***)	(*E*)-isorhapontigenin-12-*O*-β-(4'-*O*-methyl)glucopyranoside		
Methylglucosylated isorhapontigenin (**2c-*Z***)	(*Z*)-isorhapontigenin-12-*O*-β-(4'-*O*-methyl)glucopyranoside		
Methylglucosylated taxifolin (**3c**)			
Methylglucosylated taxifolin (**3c-epi**)			
Methylglucosylated quercetin (**3d**)			
Piceatannol dimer	Longusol C		
Isorhapontigenin dimer (**2e-Z**)	(*Z*)-bisisorhapontigenin A		
Isorhapontigenin dimer (**2d-*E***)	shegansu B		
Isorhapontigenin dimer (**2d-*Z***)	(*Z*)-shegansu B		
6*^{13}C label resveratrol	Resveratrol-(4-hydroxyphenyl-$^{13}C_6$)	Sigma,Taufkirchen, Germany	1185247-70-4
β-Glucosidase from Almonds		Sigma	9001-22-3
Glucosidase from *Aspergillus niger*		Sigma	9033-06-1
Yeast		Sigma	MDL number: MFCD00132595
Sodium benzoate		Carl Roth, Karlsruhe, Germany	532-32-1
Streptomycin	Streptomycin Sulfate	Duchefa, Haarlem, The Netherlands	3810-74-0
Yeast nitrogen base without amino acids, but with ammonium sulfate		Sigma	Y0626
Synthetic drop-out medium	Yeast Synthetic Drop-out Medium Supplements, without leucine	Sigma	Y1376
Adenine hemisulfate		Sigma	321-30-2
D-glucose		Carl Roth	50-99-7
Yeast extract		Carl Roth	8013-01-2

Compounds	The other name	Supplier	CAS number
Bacto peptone		Gibco,Rheinfelden, Germany	
Galactose		Carl Roth	59-23-4
Potato dextrose agar		Carl Roth	
K_2HPO_4	Dipotassium phosphate	Sigma	7758-11-4
KH_2PO_4	Potassium phosphate monobasic	Carl Roth	7778-77-0
NaCl	Sodium chloride	Carl Roth	7647-14-5
$MgSO_4$	Magnesium sulfate	Sigma	7487-88-9
$CaCl_2$	Calcium chloride	Sigma	10043-52-4
$FeSO_4$	Ferrous (II) sulfate	Sigma	7782-63-0
$(NH_4)_2SO_4$	Ammonium sulfate	Carl Roth	7783-20-2
2-[N-morpholino] ethanesulfonic acid	MES	Carl Roth	4432-31-9
Glycerol		Carl Roth	56-81-5
Acetosyringone		Carl Roth	2478-38-8
Czapek-Dox Agar		Merck,Darmstadt, Germany	
Czapek-Dox Broth		Merck	
Glufosinate ammonium		TCI	77182-82-2
Mefoxin	Cefoxitin	TCI	35607-66-0

SI References

1. D. Kandasamy *et al.*, Conifer-killing bark beetles locate fungal symbionts by detecting volatile fungal metabolites of host tree resin monoterpenes. *PLoS Biol.* **21**, e3001887 (2023).
2. M. Ludwig *et al.*, Database-independent molecular formula annotation using Gibbs sampling through ZODIAC. *Nat. Mach. Intell.* **2**, 629-641 (2020).
3. K. Dührkop, H. Shen, M. Meusel, J. Rousu, S. Böcker, Searching molecular structure databases with tandem mass spectra using CSI:FingerID. *Proc. Natl. Acad. Sci. U. S. A.* **112**, 12580-12585 (2015).
4. Y. Djoumbou Feunang *et al.*, ClassyFire: automated chemical classification with a comprehensive, computable taxonomy. *J. Cheminf.* **8**, 61 (2016).
5. H. W. Kim *et al.*, NPClassifier: a deep neural network-based structural classification tool for natural products. *J. Nat. Prod.* **84**, 2795-2807 (2021).
6. K. Dührkop *et al.*, Systematic classification of unknown metabolites using high-resolution fragmentation mass spectra. *Nat. Biotechnol.* **39**, 462-471 (2021).
7. R. Sun *et al.*, Metabolism of plant-derived toxins from its insect host increases the success of the entomopathogenic fungus *Beauveria bassiana*. *ISME J.* **17**, 1693–1704 (2023).
8. V. Casado-del Castillo, A. P. MacCabe, M. Orejas, *Agrobacterium tumefaciens*-mediated transformation of NHEJ mutant *Aspergillus nidulans* conidia: an efficient tool for targeted gene recombination using selectable nutritional markers. *J. Fungi* **7** (2021).
9. Y. S. Moon *et al.*, *Agrobacterium*-mediated disruption of a nonribosomal peptide synthetase gene in the invertebrate pathogen *Metarhizium anisopliae* reveals a peptide spore factor. *Appl Environ Microbiol* **74**, 4366-4380 (2008).
10. X. Jiang *et al.*, Ring-shaped odor coding in the antennal lobe of migratory locusts. *Cell* **187**, 3973-3991.e3924 (2024).
11. G. Sellamuthu, J. Bílý, M. R. Joga, J. Synek, A. Roy, Identifying optimal reference genes for gene expression studies in Eurasian spruce bark beetle, *Ips typographus* (Coleoptera: Curculionidae: Scolytinae). *Sci. Rep.* **12**, 4671 (2022).
12. S. H. Li, B. Schneider, J. Gershenzon, Microchemical analysis of laser-microdissected stone cells of Norway spruce by cryogenic nuclear magnetic resonance spectroscopy. *Planta* **225**, 771-779 (2007).
13. L. Xie *et al.*, Methylglucosylation of aromatic amino and phenolic moieties of drug-like biosynthons by combinatorial biosynthesis. *Proc. Nat. Acad. Sci. U. S. A.* **115**, E4980-E4989 (2018).
14. X. M. Li, L. Mao, Y.-H. and Wang, Stilbenoids from the lianas of *Gnetum pendulum*. *J. Asian Nat. Prod. Res.* **5**, 113-119 (2003).
15. C. S. Yao, M. and Lin, Bioactive stilbene dimers from *Gnetum cleistostachyum*. *Nat. Prod. Res.* **19**, 443-448 (2005).
16. J. Zhan, A. A. Leslie Gunatilaka, Selective 4′-*O*-methylglycosylation of the pentahydroxy-flavonoid quercetin by *Beauveria bassiana* ATCC 7159. *BioCat. BioTrans.* **24**, 396-399 (2006).
